# The Principal Genetic Determinants for Nasopharyngeal Carcinoma in China Involve the *HLA* Class I Antigen Recognition Groove

**DOI:** 10.1371/journal.pgen.1003103

**Published:** 2012-11-29

**Authors:** Minzhong Tang, James A. Lautenberger, Xiaojiang Gao, Efe Sezgin, Sher L. Hendrickson, Jennifer L. Troyer, Victor A. David, Li Guan, Carl E. Mcintosh, Xiuchan Guo, Yuming Zheng, Jian Liao, Hong Deng, Michael Malasky, Bailey Kessing, Cheryl A. Winkler, Mary Carrington, Guy dé The, Yi Zeng, Stephen J. O'Brien

**Affiliations:** 1 College of Life Science and Bio-Engineering, Beijing University of Technology, Beijing, China; 2 Laboratory of Genomic Diversity, National Cancer Institute, Frederick, Maryland, United States of America; 3 Wuzhou Health System Key Laboratory for Nasopharyngeal Carcinoma Etiology and Molecular Mechanism, Wuzhou Red Cross Hospital, Guangxi, China; 4 BSP-CCR Genetics Core, Frederick National Laboratory for Cancer Research, Frederick, Maryland, United States of America; 5 Cancer and Inflammation Program, Laboratory of Experimental Immunology, SAIC-Frederick, Frederick National Lab, Frederick, Maryland United States of America; 6 Ragon Institute of MGH, MIT, and Harvard, Boston, Massachusetts, United States of America; 7 Department of Biology, Shepherd University, Shepherdstown, West Virginia, United States of America; 8 Laboratory of Genomic Diversity, SAIC–Frederick, NCI–Frederick, Frederick, Maryland, United States of America; 9 State Key Laboratory for Infectious Diseases Prevention and Control, Institute for Viral Disease Control and Prevention, Chinese Center for Disease Control and Prevention, Beijing, China; 10 Department of Epidemiology, Cangwu Institute for Nasopharyngeal Carcinoma Control and Prevention, Guangxi, China; 11 Oncogenic Virus Epidemiology and Pathophysiology, Institute Pasteur, Paris, France; University of Washington, United States of America

## Abstract

Nasopharyngeal carcinoma (NPC) is an epithelial malignancy facilitated by Epstein-Barr Virus infection. Here we resolve the major genetic influences for NPC incidence using a genome-wide association study (GWAS), independent cohort replication, and high-resolution molecular HLA class I gene typing including 4,055 study participants from the Guangxi Zhuang Autonomous Region and Guangdong province of southern China. We detect and replicate strong association signals involving SNPs, HLA alleles, and amino acid (aa) variants across the major histocompatibility complex-HLA-A, HLA –B, and HLA -C class I genes (P_HLA-A-aa-site-62_ = 7.4×10^−29^; P _HLA-B-aa-site-116_ = 6.5×10^−19^; P _HLA-C-aa-site-156_ = 6.8×10^−8^ respectively). Over 250 NPC-HLA associated variants within HLA were analyzed in concert to resolve separate and largely independent HLA-A, -B, and -C gene influences. Multivariate logistical regression analysis collapsed significant associations in adjacent genes spanning 500 kb (OR2H1, GABBR1, HLA-F, and HCG9) as proxies for peptide binding motifs carried by HLA- A*11:01. A similar analysis resolved an independent association signal driven by HLA-B*13:01, B*38:02, and B*55:02 alleles together. NPC resistance alleles carrying the strongly associated amino acid variants implicate specific class I peptide recognition motifs in HLA-A and -B peptide binding groove as conferring strong genetic influence on the development of NPC in China.

## Author Summary

NPC is a deadly throat cancer in China that is dependent on EBV infection. Here, we performed a 1 M SNP genome-wide association study using a large cohort of Chinese study participants at risk for NPC. Although several putative gene regions show significant associations, the strongest statistical signals involved scores of variants within the HLA region on chromosome 6. HLA poses a formidable association-genetics challenge because of extensive linkage disequilibrium, rather low allele frequencies, and multiple physically close interacting genes of diverse function. We examined over 250 NPC-HLA associated variants detected with sequence-based nucleotide alleles and amino acid variants. The multiple associations were collapsed to implicate causal signals by multivariate logistical regression to resolve allele association interaction. One operative variant was identified as the HLA-A*11:01 allele motif, specifically in the peptide binding groove, which recognizes invading antigens; a second involved two aa sites with HLA-B tracking B*13:01 and B*55:02 alleles. We synthesize these new and previous discoveries to help resolve the important gene influences on this disease.

## Introduction

Nasopharyngeal carcinoma (NPC) is an epithelial malignancy with highly variable incidence rates around the world. An estimated 84,400 incident cases of NPC and 51,600 deaths occurred in 2008 with the highest incidence in South-Eastern Asia, relative to the Americas, Europe, Africa, and Central and Eastern Asia [1]. An early indicator of NPC development is the occurrence of immunoglobulin (Ig)A antibodies to Epstein-Barr virus (EBV) capsid antigens (EBV-IgA/VCA). [2], [3] NPC incidence for individuals expressing IgA/VCA antibodies were 31.7 times higher than the incidence in the age matched general population. [4] Linkage and family studies indicated that genetic predisposition also plays an important role in NPC onset and susceptibility. [5], [6] Among host genetic markers implicated as associated with NPC, the highly variable class I human leukocyte antigen (*HLA*) genes on chromosome 6 (6p21.3) have shown a strong and consistent association with NPC risk. [7], [8], [9], [10], [11], [12], [13]_ENREF_7 HLA class I association studies across mainland China [11], [13], Taiwan [10], [12], and Singapore [8] have consistently demonstrated that HLA-A*11 and B*13 are associated with NPC resistance, while A*02 (A*02:07), A*33, B*46 and B*58 are associated with increased NPC susceptibility (Table S1). Genome-wide association studies (GWAS) have been applied to numerous complex diseases to implicate common risk variants through well powered genetic studies [14], [15], [16]. Recent GWAS also affirmed a strong HLA influence on NPC incidence and implicated four additional non-HLA genes, however extensive linkage disequilibrium across the gene dense HLA region have confounded identification of the causal association gene(s). [17], [18], [19]

To refine and extend these reports, we explore here the operative factors of genetic association for this disease in a comprehensive four step study utilizing: 1) A GWAS utilizing 591,458 SNPs resolved by Affymetrix 6.0 genotyping platform to identify gene regions associated with NPC; 2) SNP genotyping to replicate the top signals in a second independent NPC cohort; 3) High resolution *HLA* molecular genotyping to identify specific alleles and haplotypes associated with NPC; and 4) Amino acid variant analysis to fine map the major genetic determinants associated with this disease. The analyses demonstrates that two independent association signals, specifying peptide binding grove motifs in HLA-A and in HLA –B drive the signals tracked by scores of SNP and amino acid variants that are association proxies for the HLA class I NPC association.

## Results

**Figure 1:**
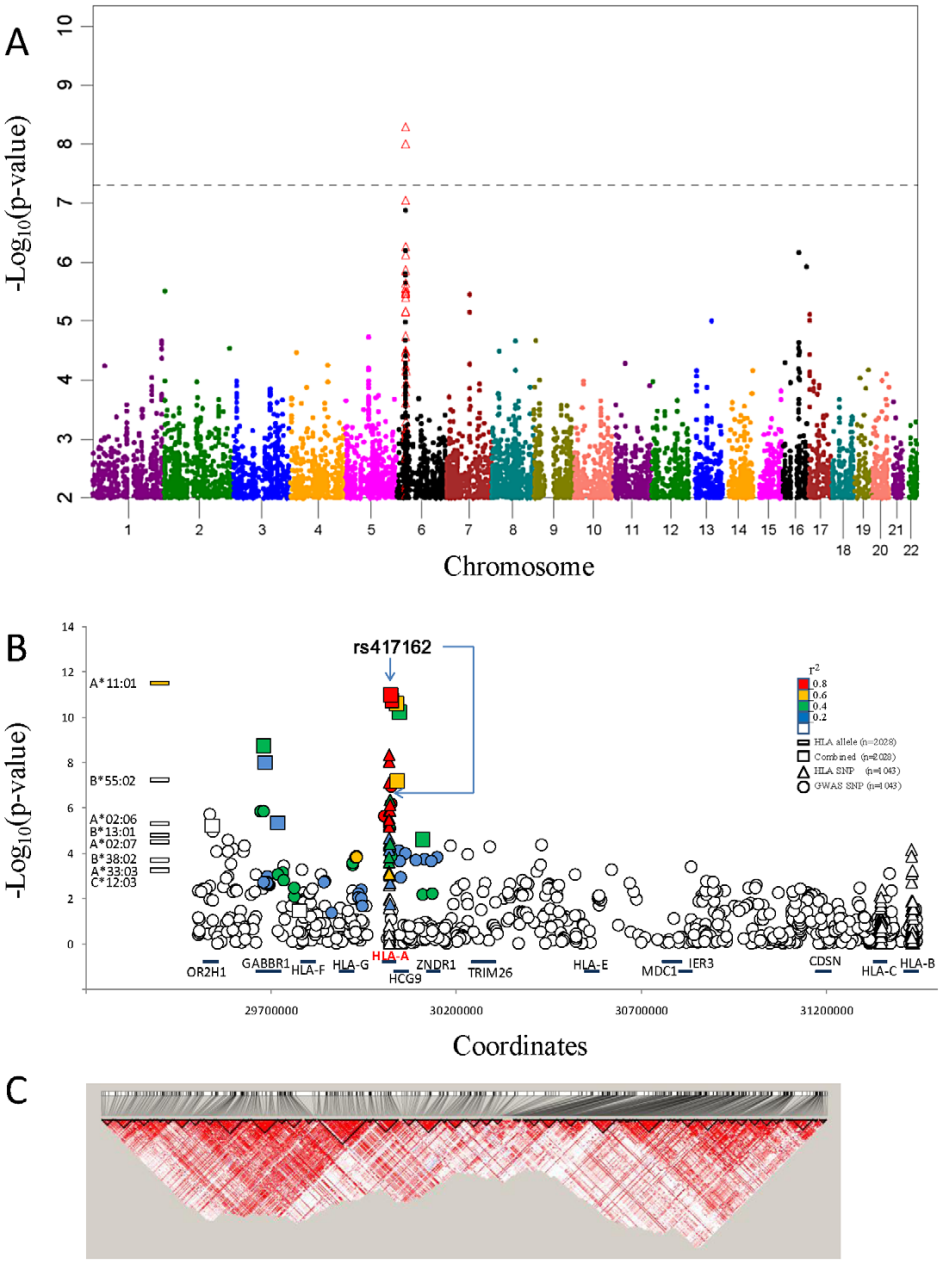
NPC associations of GWAS and Taqman validation. A.) Manhattan plot of GWAS P value association results of 591,458 SNP allele genotypes versus chromosome coordinate position (*N* = 1043 study participants; Row I in Table S3). Association p-values (-log_10_ transformed) are calculated by logistic regression in additive logistic model and plotted by genomic position. Association p-values for *HLA* SNP that were assessed by *HLA* sequence base typing for the same 1043 individuals are indicated by open red triangles (see text). B.) NPC association signal for significant HLA alleles (left) and included SNP variants on Chromosome 6.The –log_10_ p values, calculated with the logistic regression model, in GWAS and combined association tests are shown, SNPs are ordered according to the location on chromosome 6 *HLA-A* region. Color coded indicate the LD value (r^2^) of each variant with the most significant SNP rs417162. C.) Disequilibrium coefficient values for SNPs genotyped in the HLA region for NPC GWAS (N = 1043), generated with the use of Haploview software.

We performed a GWAS with 1104 southern Chinese individuals from NPC-phase II study cohorts [20], [21] (See Materials and Methods) using the Affymetrix Genome-Wide SNP 6.0 genotyping platform. After SNP- and sample-base quality control (Table S2), 591,458 SNPs genotyped in 1043 study participants (567 cases and 476 controls; Table S3, line I). Principal components analysis confirmed that all samples came from individuals of Southern Chinese ancestry (Figures S1, S2, S3, S4, S5). A quantile-quantile plot of the observed p-values showed a clear deviation from the null distribution which suggested that the most significant lower p-values are smaller than those expected by chance and likely reflect genetic association (Figure S6). The GWAS allele associations suggested a strong influence in the *HLA-A* region of chromosome 6 and weaker signals on chromosome 16 and 17 (Figure 1A).

Twenty-four SNPs (Table S4) with p-values less than 5×10^−5^ in 16 association tests (Table S5) and sixteen previous GWAS reported NPC associated SNPs (Table S6) and were selected for replication. Replication SNP genotyping was conducted in an independent Chinese cohort that included 356 NPC cases and 629 controls (Table S3, line II). Six of 40 SNPs that showed genome-wide significant NPC association and replication were within 500 kb of each other in the *MHC* region of chromosome 6 (Table 1, Table S7). The most significant SNP rs417162 (*P_combined_* = 1.1×10^−11^, OR = 0.61) is located within the *HLA-A* locus, while four additional replicated SNPs were within adjacent genes, GABBR1 and HCG9.

**Table 1:** GWAS and validation of SNPs association data in two independent NPC cohorts.

				GWAS (*N* = 1,043)			Validation (*N* = 985)			Combined (*N* = 2,028)	
SNP name	Gene	Chr.	MA†	MAF‡	*P*-value	OR(95% CI)	MAF	*P*-value	OR(95% CI)	*P-*value	OR (95% CI)
rs417162	*HLA-A*	6	C	0.26/0.37	1.13E-07	0.58(0.48–0.71)	0.26/0.35	3.75E-05	0.63(0.50–0.78)	1.05E-11	0.63(0.53–0.75)
rs2517713*	*HLA-A*	6	G	0.26/0.37	3.03E-07	0.57(0.46–0.71)	0.26/0.35	2.61E-05	0.62(0.50–0.78)	1.63E-11	0.60(0.52–0.70)
rs9260734*	*HCG9*	6	A	0.22/0.32	5.90E-07	0.57(0.45–0.71)	0.21/0.32	1.32E-05	0.59(0.47–0.75)	2.63E-11	0.59(0.50–0.69)
rs5009448*	*HCG9*	6	T	0.26/0.35	1.09E-05	0.63(0.51–0.77)	0.24/0.35	4.43E-07	0.56(0.45–0.70)	6.40E-11	0.61(0.53–0.71)
rs2267633	*GABBR1*	6	G	0.17/0.26	1.43E-06	0.58(0.47–0.73)	0.17/0.24	2.77E-04	0.63(0.49–0.81)	1.89E-09	0.61(0.52–0.72)
rs29230*	*GABBR1*	6	C	0.17/0.25	2.37E-05	0.60(0.47–0.76)	0.17/0.24	6.02E-04	0.65(0.50–0.83)	9.48E-09	0.61(0.52–0.72)

* : Replication SNPs that not included in Affymetrix Genome-Wide SNP Array;

† : MA, Minor allele;

‡ : MAF, Minor allele frequencies;

A fine-grain view of the pattern of GWAS SNPs around the *HLA-A* locus is illustrated in Figure 1B. An extensive cluster of associated *HLA-A* region SNPs that approach or exceed genome-wide association threshold p-values (p<10^−8^) is apparent within 500 kb including associations in the adjacent *HCG9* and *GABBR1* genes (Figure 1C). The strong linkage disequilibrium (LD) across the HLA region is well known, which raises the question whether these *HLA* region associations represent single, multiple independent, or LD-proxy driven associations.

To characterize the *HLA* association with NPC in finer detail, high-resolution molecular *HLA* genotyping was performed on 4055 study participants; 1043 subjects in the discovery cohort, 985 subjects in the replication cohort, and 2027 subjects that comprise the remainder of cohort (Table S3). [20], [21] NPC cases, controls with EBV-IgA/VCA positive, and controls with EBV-IgA/VCA negative were examined for association of *HLA* alleles and *HLA* haplotypes with NPC risk and EBV IgA/VCA antibody status. Three *HLA* alleles, A*11:01, B*38:02 and B*55:02 showed the most significant association with NPC (*P* = 1.7×10^−19^, *P* = 7.0×10^−11^, and *P* = 1.6×10^−10^ respectively; Table 2). In addition to the strong *HLA-A* and *-B* associations, there was a moderate association of HLA-C*12:02 allele as well (*P* = 4.3×10^−5^; Table 2). NPC associated *HLA-A-B-C* haplotypes which included HLA allele combinations from Table 2 were also apparent (Table S8).

**Table 2:** Gene frequencies (%) of the HLA-A, -B, and -C alleles detected and association analysis (*N* = 4,055).#

		Allele Frequencies			NPC vs. Control		NPC VS. EP. Controls		NPC VS. EN. Controls	
Allele Name	Amino Acid Binding Motifs*	NPC Patients (*N* = 1405)	EP. Controls† (*N* = 1288)	EN. Controls‡ (*N* = 1362)	OR (95% CI.)	P Value	OR (95% CI.)	P Value	OR (95% CI.)	P Value
***HLA-A***										
*02:03* §	.[LV]……[LI]	15.80(444)	10.87(280)	15.01(409)		Ns	1.52(1.29–1.79)	4.49E-07		Ns
*02:06*	.[VQ]……[VS]	2.62(139)	2.21(57)	3.01(82)	1.65(1.28–2.13)	1.15E-04	1.91(1.38–2.65)	1.00E-04		Ns
*02:07*	.[L-]……[VL]	16.48(463)	13.32(343)	11.56(315)	1.38(1.21–1.57)	1.52E-06		Ns	1.49(1.28–1.74)	3.98E-07
*11:01*	.[YT]……[K-]	20.25(569)	30.12(776)	29.11(793)	0.59(0.53–0.66)	1.72E-19	0.57(0.50–0.65)	1.43E-16	0.61(0.53–0.69)	3.13E-14
*33:03*	[DE]I……[R-]	17.69(497)	15.76(406)	12.37(337)	1.36(1.19–1.54)	3.14E-06		Ns	1.57(1.35–1.84)	1.01E-08
***HLA-B***										
*13:01*	.[–]……[–]	8.04(226)	12.27(316)	10.87(296)	0.66(0.56–0.77)	4.52E-07	0.62(0.51–0.74)	3.15E-07	0.71(0.59–0.86)	3.16E-04
*27:04*	R[R-]……[LF]	0.53(15)	0.97(25)	1.69(46)		Ns		Ns	0.31(0.17–0.55)	8.56E-05
*38:02*	.[–]……[–]	12.21(343)	6.83(176)	8.48(231)	1.67(1.43–1.94)	6.96E-11	1.88(1.55–2.28)	1.96E-10	1.51(1.26–1.80)	5.90E-06
*46:01*	.[MI]……[YF]	19.18(539)	17.97(463)	14.46(394)		Ns		Ns	1.39(1.21–1.60)	4.77E-06
*55:02*	.[P-]……[AV]	1.00(28)	3.38(75)	3.67(100)	0.27(0.18–0.40)	1.57E-10	0.28(0.18–0.43)	7.38E-09	0.26(0.17–0.40)	5.02E-10
*58:01*	.[TS]……[WF]	16.65(468)	15.49(399)	12.22(333)	1.47(1.26–1.71)	2.38E-04		Ns	1.43(1.23–1.67)	1.47E-06
***HLA-C***										
*01:02*		22.03(619)	21.35(550)	17.99(490)		Ns		Ns	1.28(1.12–1.46)	2.16E-04
*03:02*		16.37(460)	15.22(392)	11.97(326)	1.28(1.12–1.46)	2.05E-04		Ns	1.48(1.26–1.73)	1.30E-06
*07:02*		21.64(608)	16.89(435)	19.38(528)	1.25(1.11–1.40)	1.84E-04	1.37(1.19–1.58)	1.65E-05		Ns
*12:02*		0.93(26)	1.59(41)	2.83(77)	0.41(0.27–0.63)	4.28E-05		Ns	0.31(0.20–0.49)	4.14E-07
*12:03*		0.78(22)	2.14(55)	1.76(48)	0.41(0.25–0.65)	1.47E-04	0.37(0.22–0.62)	1.37E-04		Ns

* : Amino acid motifs from Lund et al., 2004.

† : EP. controls, the controls of NPC free and EBV IgA/VCA antibody positive.

‡ : EN. controls, the controls of NPC free and EBV IgA/VCA antibody negative.

§ : Allele also showed significant in the comparison of EP. controls VS. EN. controls, p = 7.81E-06, OR = 0.69(0.59–0.81).

# A preliminary analysis of HLA association in phase I was previously reported (Tang et al., 2010). Now, HLA associations with phase I and phase II (See Materials and methods) are presented separately in Table S12.

**Figure 2:**
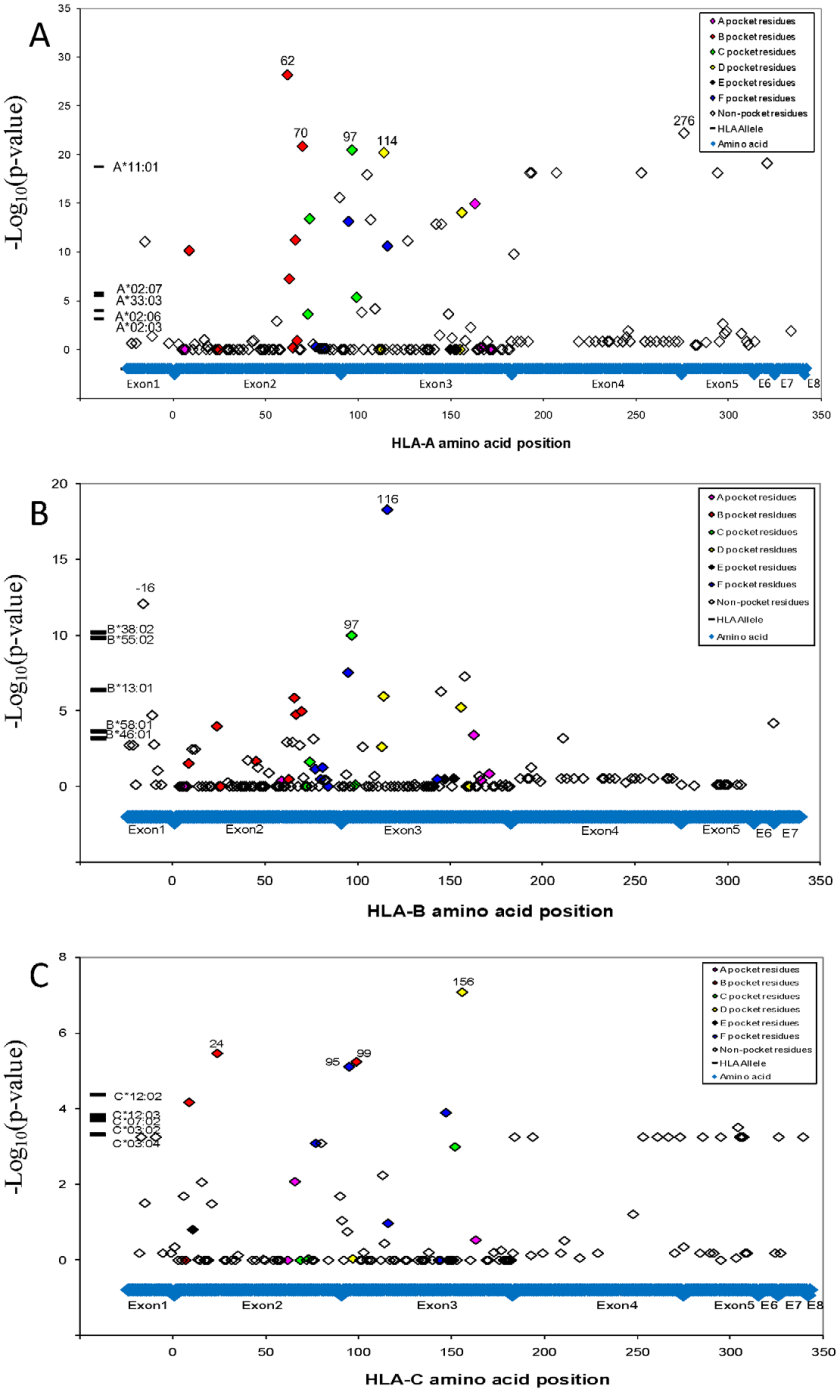
NPC associations of HLA alleles and amino acid variants. A.) NPC associations of alleles and amino acid variants at HLA-A locus; B.) NPC associations of alleles and amino acid variants at HLA-B locus; C.) NPC associations of alleles and amino acid variants at HLA-C locus. Genetic association of HLA alleles and amino acid sites were calculated (*N* = 4055 study participants; Line V in Table S3). For amino acid positions with more than two alleles, p-value for the omnibus test that tests all amino acid alleles simultaneously (with >1 degrees of freedom) for association to control.

Functional variation of different MHC molecules to bind peptides and activate effector cells in the immune system underlies their association with disease. [22], [23]_ENREF_18 To identify the specific site driving the NPC *HLA* associations, HLA gene sequence was translated to identify amino acid variants using a web-based software of the Immunology Database and Analysis Portal (ImmPort) system [24]. Genetic association of 284 detected *HLA* amino acid variant within the three *HLA* class I genes implicated the most significant NPC association of glutamine (Gln, Q) at amino acid position 62 of *HLA-A* gene (*P* = 1.2×10^−24^, OR = 0.59; Figure 2A; Tables S9 and S10) which marks HLA-A*11, however there are 25 additional amino acid sites I in *HLA-A* that also show exceed genome wide significance (*P*<_ENREF_1810^−8^; Figure 2A). The *HLA-B* signal centered on amino acid Leucine (Leu, L) at amino acid position −16 and 116 (*P* = 1.7×10^−13^ and 2.4×10^−13^, OR = 0.65 and 0.63; Table S10), which marks B*13:01 and B*55:02. A far less significant association was observed for the amino acid residue Tryptophan (Trp, W) at amino acid position 156 for *HLA-C* (*P* = 1.4×10^−9^, OR = 0.47;). Amino acid residues that correspond to the antigenic peptide binding groove residues showed the strongest association (See color code in Figure 2A–2C) suggesting that the peptide binding groove and function are major genetic factors for NPC risk.

**Figure 3:**
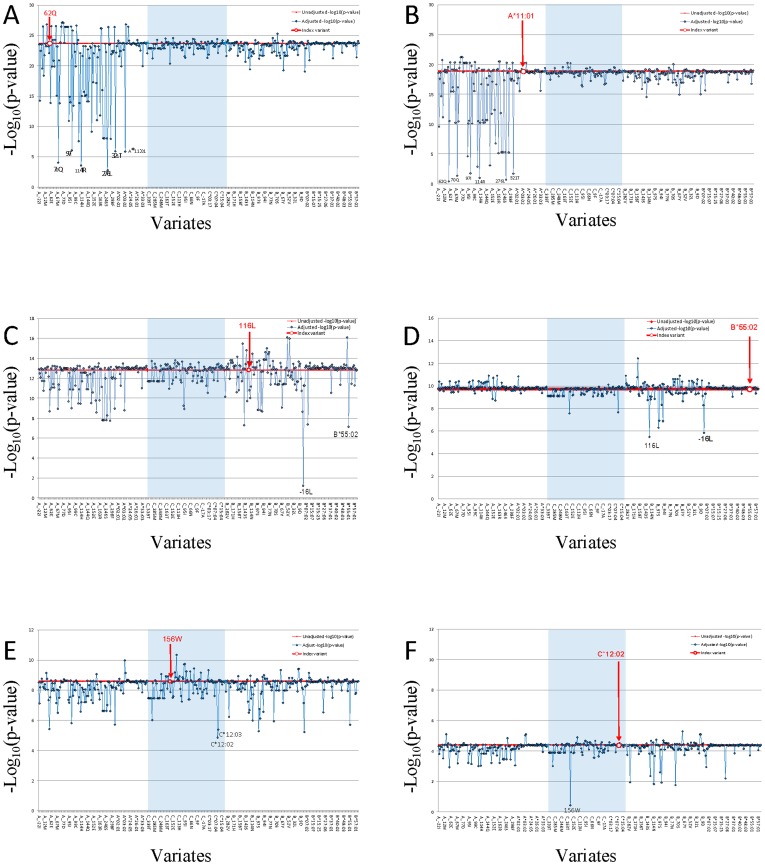
Proxy variant analysis for the strongest aa-variants or HLA allele in HLA-A, in HLA-B, and in HLA-C. Genetic association of each HLA amino acid variant was calculated. Study participants in both phase I and phase II (N = 4055 study participants; Line V in Table S3). Multivariate conditional logistic regression analysis was performed to compute amino acid variants (A, C, and E) or HLA alleles (B, D, and F) association p-value. The *HLA* typing data set (*N* = 4055 study participants; Line V in Table S3) were used PLINK to examine the residual effect of index amino acid variant or HLA allele while using other amino acid variant or HLA allele as a covariate, and we adjusted the results for age and gender. The index amino acid variant or HLA allele was marked with bold red font. The red line indicated unadjusted –log_10_ p of index. Three HLA class I genes regions were separated by light blue block of HLA-C gene between HLA-A and HLA-B. The X-axis is amino acid or HLA allele covariate, ranking by their coordinate. HLA alleles were group together ranking by allele names in each HLA class I gene locus. The Y-axis is the –log_10_p of index variant adjust by covariate. Independent variants should not change the adjusted p-values from the strong unadjusted values of the index variant, while LD-proxies would reduce their p-values appreciably depending upon the strength of LD.

Given the plethora and complexity of *HLA* genetic associations plus the extensive LD within *HLA*, we attempted to resolve which *HLA* region SNPs and aa-variants represent proxy variants for one or more functional sites (i.e. they were tracking by LD) and which represent independent (non-LD) association signals using a multivariate logistic regression analyses [25]. Strongly associated aa-variants (Figure 2 and in Tables S9 and S10; e.g *HLA-A*-62Gln) were analyzed in a multivariate logistical regression analysis adjusting statistically for non-random influence of each of the adjacent aa-variants (Figure 3; also in Table S11). A dramatic reduction of association p-value significance for the strongest HLA-A aa variant, *HLA-A*-62Gln, is observed when this model is adjusted for adjacent aa-variants within and about the HLA-A gene but are not diminished by adjusting for variants in HLA-B or HLA-C. Thus, we conclude that there is a single association signal in *HLA-A* tracked by several dozen proxy aa/SNP variants within the *HLA-A* region. When HLA-A*11:01 is the index allele, the extreme NPC association signal is diminished to 0.1–0.01 by *HLA-A* aa variants as well as each SNP in the genes adjacent to *HLA-A* locus (See Figure 3B and HLA-A*11:01 column in Table S11). This multivariate dependence plus the knowledge that HLA-A*11:01 carries the five strongly significant associated aa variants (62Gln, 276Leu, 114Arg, 70Gln, and 97Ile) in the peptide binding groove and reaches the highest significance in allele level would support the conclusion that the causal association is driven by the HLA-A*11:01 allele (Table 2, Table S10, Figure S7).

A multivariate logistical regression analysis for *HLA-B* variants indicates that HLA-B associations are independent from the *HLA-A* signals and driven by two amino acid sites in strong LD with each other (*HLA-B*: -16Leu and 116Leu; *P* = 1.7×10^−13^ and 2.4×10^−13^) (Figure 3C and Table S9). The most significant HLA-B signal is located at amino acid position 116 (Figure 2B). The amino acid variant HLA-B-116Leu is present in the two strongly associated protective *HLA-B* alleles B*13:01 and B*55:02, but the encoded amino acid in the associated suscetible allele HLA-B*38:02 is Phenylalanine (Table S9). It is also relevant that the same location of *HLA-B* amino acid position 116 has also been definitively implicated as the single aa site that drives high susceptibility of the HLA-B*35 association with very rapid AIDS progression in HIV-1 infected European Americans. [26], [27] It seems that this variant influences *HLA* peptide repertoire recognition and/or presentation for both HIV and EBV infections. The amino acid substitution in the heavy chain at position 116 could abolish the ability of P9 picket of HLA-B*35:01 to bind tyrosine but preferentially accommodate smaller hydrophobic residues such as methionine, valine, or leucine at the carboxy-terminal anchor had been shown by peptide-binding assays. [28]

The *HLA-C* signal is ten logs weaker than HLA-A or HLA-B and is diminished slightly by adjusting for *HLA-A* or *HLA-B* variants (Figure 3E, Table S11). Further, the most significant HLA-C alleles (HLA-C*03:02 and -C*12:02) track HLA-A and -B alleles in the haplotype analyses (Table S8), suggesting the HLA-C association are likely proxies of the stronger HLA-B and -A associations. We interpret these cumulative data as suggestive that there are two robust independent *HLA* association signals with NPC development: *HLA-A* including at least five amino acid position in 62Gln, 70Gln, 97Ile, 114Arg and 276Leu carried by HLA-A*11:01 and *HLA-B* including the -16Leu and 116Leu-bearing alleles.

Our GWAS analysis also provided an opportunity to inspect regions of the genome outside *HLA* that had been implicated in previous NPC studies. The results (Table S6 and Table 1) offer strong supportive confirmation of SNPs in the *HLA-A* gene region (including the adjacent *HCG9*, and *GABBR1* genes) as suggested by previous GWAS. [18], [19] However, our SNP replication (Table S6) and multivariate logistical regression analysis (Figure 3; Table S11) indicate that all these associations are by and large proxies for the primary functional aa variants association in our cohort. We also replicated the *TNFRSF19*, *MDS1-EVI1*, *CDNK2A/2B* gene associations [19] in our cohort (p = 1.5×10^−5^; 5.0×10^−5^ and 5.6×10^−3^ respectively) although these genes did not achieved genome wide significance (Table S6). The ITGA9 association reported by Ng et al^14^ was not replicated in our cohort (Table S6).

## Discussion

We present and interpret a 1 M SNP GWAS, in subjects from Guangxi Zhuang Autonomous Region and Guangdong province of southern China, where perhaps the highest recorded NPC incidence has been found. [3], [4], [20], [21] Multiple genome wide significant association signals were evident with the HLA gene region and in a few other chromosomal regions (Figure 1A). Because the HLA region is complex and displays extensive LD, we sought to resolve the causal association signals with several different approaches. These included replication in an independent cohort from the same area, sequence based gene typing of the HLA-A, -B and -C genes, and analysis of sequence based nucleotide alleles as well as 284 amino acid site variants across the HLA genes (Figure 2). We compared association signals of SNPs, aa variants, HLA- alleles defined by molecular typing and associated HLA –A, -B and C haplotypes. To resolve the operative variants from proxies that track signals by LD, we enlisted a multivariate logistical regression of alleles and site variants with the strongest signals (Figure 3, Table S11). Finally we revisited and attempted replication in our cohort reports from other NPC gene associations including GWAS recently published, [17], [18], [19] (TNFRSF19 -CHR 13, MDS1-EVI1-CHR 3, and CDNK2A/2B- CHR 9; Table S6) affirming gene influence that are important in this disease.

In the present study, two independent powerful association signals within the HLA region were resolved for NPC, amidst a background of scores of adjacent associated LD-proxy variants. The first influence involved the HLA-A*11:01 allele sequence and function, specifically in the peptide binding groove, which recognizes invading antigens. This conclusion derives from several lines of evidence: 1.) HLA-A*11:01 is a common allele in the populations (*F* = 0.25) and is the only “protective” allele with genome wide significant *HLA-A* signal (OR = 0.59; *P* = 1.7×10^−19^; Table 1) the strongest of all HLA alleles. 2.) HLA-A*11:01 is included in the significantly associated protective HLA haplotypes (Table S8); 3.) 100% of associated SNPs and aa variants about HLA-A, including those in adjacent genes, namely the *HCG9*, and *GABBR1* loci, are proxies HLA-A*11:01(Figure 3; Table S11); 4.) HLA-A*11:01 carries five strongly significant associated aa variants (62Gln, 276Leu, 114Arg, 70Gln, and 97Ile) in its peptide binding groove (Table S10). Taken together, the HLA-A association is centered on HLA-A*11:01 allele function and tracked by internal and closely linked proxy aa and SNP variants.

It may also be relevant that the sequence of HLA-A*11:01 allele (*F* = 0.25 in this population) differs by only one amino acid residue (Lys19Glu) from that of the HLA-A*11:02 allele (*F* = 0.04), yet HLA-A *11:02 shows no apparent association with NPC onset. Both HLA-A*11:01 and HLA-A*11:02 alleles share a unique peptide binding motif signature of “.[YT]……[K-]” (Table 2) and an identical sequence within the defined residues of the antigen recognition site. [23] Since the only Lys19Glu residue difference between the two HLA-A*11 alleles is outside the peptide binding region, the possibility of an alternative mechanisms for NPC pathogenesis, e.g. *HLA-A/KIR* innate immunity involvement [29] or dendritic cell interaction, [30] should be considered and explored in future studies.

We further demonstrate an independent *HLA-B* signal derived from three representive alleles, two protective alleles (B*13:01 and B*55:02) and a suscetible allele B*38:02. Both *HLA-A* and -*B* associations involve functional variants in the antigenic recognition site. The strongest *HLA-B* aa site implicated is identical to the single aa site that mediates HLA-B*35 rapid AIDS progression reported previously. [26], [27], [30] All the NPC associations were genome wide significant in one or more analyses, replicated internally in independent Guangxi cohorts and externally in other genetic association studies in Asia. Our study demonstrates a powerful genetic influence on NPC onset in Chinese people, implicates explicit *HLA* alleles, peptide recognition motifs, and aa variants that confer strong genetic influence on the development of NPC in China. *HLA* disease associations are likely to involve multiple mechanisms. A recent study in HIV disease showed that allelic diversity of HLA-C can cause variation in the level of surface expression of the HLA-C molecule, which in turn affects viral load control and disease progression [31], perhaps through both HLA-restricted CTL responses and HLA/KIR-mediated NK cell activities. The functional basis for HLA associations with NPC should be explored fully, now that the genetic basis of this disease is well-characterized, in hopes of explaining the complex HLA association with NPC in the Chinese population.

## Materials and Methods

### Ethics statement

This study were approved by institutional ethics review committees at the relevant organizations, and conducted with the IRB approval (NIH IRB -02-C-N056). Informed consent was obtained from all study participants.

### Study cohorts

A total of 4055 study subjects (1405 NPC cases and 2650 controls, Table S3) were recruited in two independent collection phases: phase I -April 2000 to June 2001 and phase II-November 2004 to October 2005, from the Guangxi autonomous region and Guangdong province of southern China. [20] All study subjects were of Han ethnic origin and reside in the catchment area of the Xijiang River. IgA antibodies to EBV capsid antigen (EBV-IgA/VCA) were confirmed by serologic testing for all the subjects at the time of study enrollment. In phase I, the case group included 356 unrelated patients with biopsy-confirmed NPC. The mean age was 50.1 years (range 19–80), 95.5% of them were EBV-IgA/VCA antibody positive. Controls included case spouses or geographically matched residents who were NPC free at the time of study enrollment. An additional 422 adult children of the study subjects were enrolled for haplotype inference and for quality control assessment, but they were excluded in association analyses. In phase II, the case group included 1049 unrelated patients with biopsy-confirmed NPC. The mean age was 46.3 years (range 10–77), 96.3% of them were EBV-IgA/VCA antibody positive. Two distinct NPC-free control groups were included; one group was positive (N = 1001) and the other negative (N = 1020) for the EBV-IgA/VCA antibody. The mean ages were 46.1 and 46.6, for the antibody positive and negative controls groups. All study subjects were self-reported Guangxi or Guangdong provincial ancestry for either maternal or paternal ancestry for at least three generations.

### Genome-wide SNPs genotyping

A total of 598 NPC cases and 506 controls were randomly selected from phase II enrollment cohort for GWAS analysis. DNA was extracted from whole blood by traditional phenol/chloroform method with phase Lock Gel tube (Qiagen, MaXtract High Density, catalog # 129065). The genome-wide genotyping experiments were conducted by using the Affymetrix Genome-Wide SNP Array 6.0 genotyping platform. 325 nanograms of DNA per sample were prepared for both Sty1 and Nsp1 restriction enzyme digestion for this assay, genotyping in according to the manufacturer's instructions.

### Quality control

#### Patient DNA

Genotyping analysis of GWAS samples was performed in Genotyping Console 3.0.2 for first-pass quality control. The contrast quality control (CQC) metrics were computed by the Affymetrix software. We attempted to ascertain genotypes for 1,104 NPC study subjects. 11 samples failed genotyping or were removed because of failing CQC (<0.4) or call rate (<90%). An additional 17 samples failed to meet further QC filters which included heterozygosity < = 25% and at least one enzyme specific CQC value (Nsp1 or Sty1) >1. 24 samples were removed because the genotypes determined in the GWAS were discordant with genotypes previously determined by the Laboratory of Genomic Diversity for candidate gene studies. The gender of the samples was determined from the heterozygosity of X chromosome SNPs (Affymetrix and PLINK software) and by the ratio of the mean intensity of the copy number probes on the Y chromosome to the mean intensity of a subset of copy number probes on the X chromosome (Affymetrix software). Four samples were removed because the gender determined from the genotypes was discordant with the gender provided by the cohort. Identity by descent (IBD) statistics computed using PLINK software were used to detect cryptic familial relationships. Four first degree relationships were observed (3 full sib pairs and 1 parent-offspring pair). For each of these pairs, the sample with the lowest call rate was removed. A fifth sample was removed because the IBD statistics were consistent with that individual having a first cousin relationship with five other subjects. After sample filtering, a number of 1043 subjects were remained for further analyses (Table S2).

#### SNPs

Genotypes were ascertained for the 934,968 SNPs on the Genome-wide SNP 6.0 platform using the command line option of Affymetrix software. NetAffx version 30 was used for SNP annotation. This data set uses map positions based on the NCBI Build 36.1/UCSC hg18 human genome assembly. Unsupported SNPs, QC SNPs, non-autosomal SNPs, and remaining redundant SNPs were identified from the annotation data set and removed. Genotypes from 8 CEPH and 10 NPC mother/father/offspring trios were checked for errors in Mendelian inheritance using PLINK software. SNPs having 2 or more errors in either group of samples were rejected. Per-SNP call rate, Hardy-Weinberg test statistics, and minor allele frequencies were computed for 1,043 NPC study samples (see Table S2A) using PLINK software. SNPs not meeting the criteria shown in the table were removed (Table S2B).

### Replication genotyping

Validation and replication genotyping of significant SNPs from our GWAS and from other studies was performed using the ABI Taqman genotyping assays by design in accordance with the manufacturer's instructions. The sequence detection software (SDS2.2, Applied Biosystems, Foster City, CA, USA) was used for allelic discrimination and confirmed the good quality of genotyping.

### HLA typing

High resolution HLA molecular typing was performed for all 1,405 unrelated NPC cases and 2,650 unrelated controls from both enrollment cohorts. HLA class I alleles were characterized using a PCR-SSOP (sequence-specific oligonucleotide probe) typing protocol developed by the 13th International Histocompatibility Workshop [32] for the first enrollment study cohort (N = 985), and using a DNA sequence-based typing (SBT) protocol in the second enrollment study cohort (N = 3070). The sequencing analysis was performed using the ABI Big Dye Terminator Cycle Sequencing Kit and the ABI3730xl DNA analyzer (Applied Biosystems, Foster City, CA). HLA alleles were assigned on the basis of the sequence database of known alleles with the help of the ASSIGN software developed by Conexio Genomics (Conexio Genomics, Western Australia, Australia). Ambiguous heterozygous genotypes were resolved by additional PCR and sequencing procedures using allele-specific PCR primers to selectively amplify only one of the two alleles.

Haplotype of HLA-A, HLA-B and HLA-C allelic combinations were assessed using 422 children of the phase I study subjects in 179 patients and 379 controls. Based on expectation maximization algorithm to generate maximum likelihood estimation haplotype, we observed 90% accuracy on HLA-A-B-C haplotypes, 91% on HLA-A-B and HLA-A-C haplotypes, and 99% on HLA-B-C haplotypes. For the remaining NPC cases and controls, HLA haplotypes were assigned by population-based estimation methods of PROC HAPLOTYPE in SAS/Genetics package.

### HLA amino acid variant definition

Because the most significant NPC associated SNPs is located on the HLA class I region (see Results), an amino acid analysis was carried out to evaluate the role of functional relative amino acid residues in HLA associations. From our high resolution HLA genotyping results, we were able to define corresponding amino acid sequences for all study subjects. The amino acid variants in HLA class I genes were defined by using web-based software of the Immunology Database and Analysis Portal (ImmPort) system [24].

### Proxy SNP–variant analysis

We have used the method of testing each variant for reduced-p-values in multi-variants models resulting from co-linearity of variants to recognize LD and independence of signals in the association of NPC with HLA-A, -B and -C. Multicollinearity in logistic regression models is a result of strong correlations between variables. The existence of multicollinearity (high r^2^) between variants inflates the variances of the parameter estimates. That will likely result in lowered p-values for a given SNP that was determined to be in significant association with NPC when tested signally. We used a reduction in significance as an indicator that two variants were in strong LD, and therefore not independent signals as has been done by recent authors [18]. This gives us a general idea about independence of the signals within HLA and adjacent genes within the context of the disease association. However, we recognize that although multicollinearity may lower magnitudes of regression coefficient estimates and resulting p-value significance in cases of LD, this method may be subject to error such as when a rare SNP on a haplotype does not have a large effect on the model. These methods are provided as an indicator of independence, but not as a definitive measure in our understanding of the disease.

### Statistical analysis

We performed logistic regression model analysis for all SNPs passing the quality control filters, using a Cochran-Armitage trend, co-dominant, dominant, recessive, and allelic model taking the number of copies of the rare allele 0, 1 or 2, as the explanatory variable. The comparisons were conducted between NPC cases and NPC free controls, NPC cases and NPC-free but EBV-IgA/VCA antibody positive controls (EP controls), NPC cases and NPC-free but EBV-IgA/VCA-antibody negative controls (EN controls), EP controls and EN controls respectively. Population structure/stratification was assessed using the Principal Components Analysis (PCA) module of Eigensoft software [33]. Study samples were first run together with HapMap individuals of European, African, and Asian descents to identify any potential admixed individuals. Later, PCA analyses of only the study samples were conducted. Initially all autosomal SNPs that passed the quality control filters were used to estimate the contribution of each SNP to the top ten eigenvectors. Previously reported correlated genome regions [19] (such as on chromosomes 6, 8, and 11) were observed and excluded from the following PCA analyses. Moreover, to avoid any confounders due to LD among the SNPs, the genotype data was pruned to 90 K independent SNPs distributed throughout the genome by PLINK prior to the PCA analyses. The logistic regression analysis performed using PLINK [25], controlling for gender, age and the first three eigenvectors; the significance was evaluated using the log likelihood test. SNPs were sorted according to the lowest P-value in a combined set of samples in one of these models. The chi-square tests were used for testing case-control association for allele effects. HLA allele frequencies were calculated based on observed genotypes; HLA-A, -B and -C haplotype were assigned based on maximum likelihood estimation using the SAS/Genetics HAPLOTYPE procedure. The effect of HLA alleles on the development of NPC and EBV-IgA/VCA antibody was evaluated by computing odds ratios (OR) and 95% confidence intervals (CI) using logistic regression. For HLA allele and haplotype test, P values were calculated by logistic regression and then corrected by the Bonferroni, which was multiplied by the number of all detected alleles or haplotypes. Significance was considered at P<0.05 after correction.

## Supporting Information

Figure S1 Plots of principal components from the PCA for genetic matching. Plot of the first two PCs from the PCA (N = 1043 study participants; Row I in Table S3)and 206 HapMap individuals, including 57 Yoruba in Ibadan, Nigeria (YRI), 44 Japanese in Tokyo, Japan (JPT), 45 Han Chinese in Beijing, China(CHB) and 60 CEPH (Utah residents with ancestry from northern and western Europe) (CEU).
(TIF)

Figure S2 Plots of principal components from the PCA for genetic matching. Plot of the first two PCs from the PCA (N = 1043 study participants; Row I in Table S3), 44 Japanese in Tokyo, Japan (JPT), and 45 Han Chinese in Beijing, China (CHB).
(TIF)

Figure S3 Plots of the first two principal components from the PCA of 1,043 NPC study samples for genetic matching.
(TIF)

Figure S4 Plot of the first and third PCs from the PCA of 1,043 NPC study samples for genetic matching.
(TIF)

Figure S5 Plots of the second and third PCs from the PCA of 1,043 NPC study samples for genetic matching.
(TIF)

Figure S6 Quantile-quantile plot showing the distribution of observed statistic by allelic test for association of each SNP with NPC.
(TIF)

Figure S7 Schematic overview of the structure of HLA-A*11:01 in complex with SARS nucleocapsid peptide. The α1-helix is shown in blue; α2-helix is shown in purple; β-pleated sheet is shown in lightblue. The significantly associated exposed positons of the peptide in the binding groove are shown in red with label indicated. Green balls in the binding groove indicate SARS nucleocapsid peptide K[T]FPPTEP[K], notice the P2 and P9 residues with red. The crystal structure of 1X7Q [13] was download from PDB database (http://www.pdb.org/pdb/home/home.do). (a) Top view, (b) P2 residueThreonine (Thr, T) in peptide bingding groove, (c) P9 residue Lysine (Lys, K) in peptide binding groove. All figures were prepared with PyMOL [14].
(TIF)

Table S1 NPC associated classical HLA class I alleles.
(DOCX)

Table S2 Sample and SNP filtering for this study.
(DOCX)

Table S3 Summary of samples used in GWAS, replication and HLA analysis.
(DOCX)

Table S4 GWAS Results (N = 1,043 subjects) using different test combinations and genetic models for 24 SNPs with the lowest p values.
(DOCX)

Table S5 List of 16 genetic association tests.
(DOCX)

Table S6 Replication of previously reported NPC associated GWAS SNPs and candidate genes in these cohorts.
(DOCX)

Table S7 40 GWAS and validation of SNPs association data in two independent NPC cohorts.
(DOCX)

Table S8 Significant explicit and imputed haplotype NPC association analysis.
(DOCX)

Table S9 Association results for the amino acid residues in each of the classical HLA loci in all study subjects.
(DOCX)

Table S10 The five most significant NPC associated amino acid in each HLA class I locus.
(DOCX)

Table S11 Multivariate logistic regression analysis for significant variant and HLA class I alleles.
(DOCX)

Table S12 HLA-A, -B and -C allele association analysis separated into in phase I and II analyses.
(DOCX)
