# Supplementary figures and images for: The Principal Genetic Determinants for Nasopharyngeal Carcinoma in China Involve the HLA Class I Antigen Recognition Groove
Source: PLoS Genet. 2012 Nov 29;8(11):e1003103. doi: 10.1371/journal.pgen.1003103 (PMC3510037; doi:10.1371/journal.pgen.1003103)

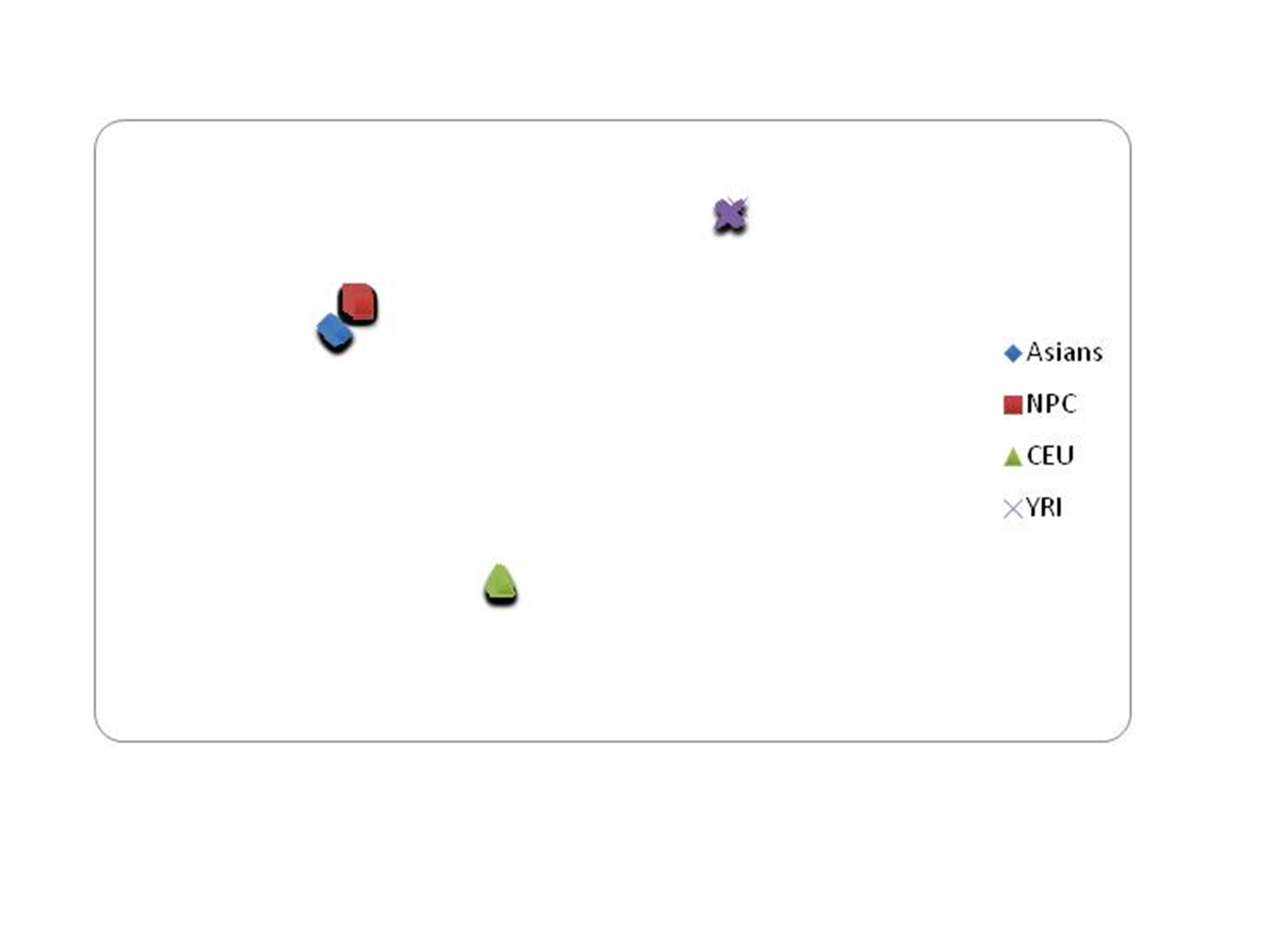

Supplement: Figure S1 — Plots of principal components from the PCA for genetic matching. Plot of the first two PCs from the PCA (N = 1043 study participants; Row I in Table S3)and 206 HapMap individuals, including 57 Yoruba in Ibadan, Nigeria (YRI), 44 Japanese in Tokyo, Japan (JPT), 45 Han Chinese in Beijing, China(CHB) and 60 CEPH (Utah residents with ancestry from northern and western Europe) (CEU). (TIF) [file pgen.1003103.s001.tif]

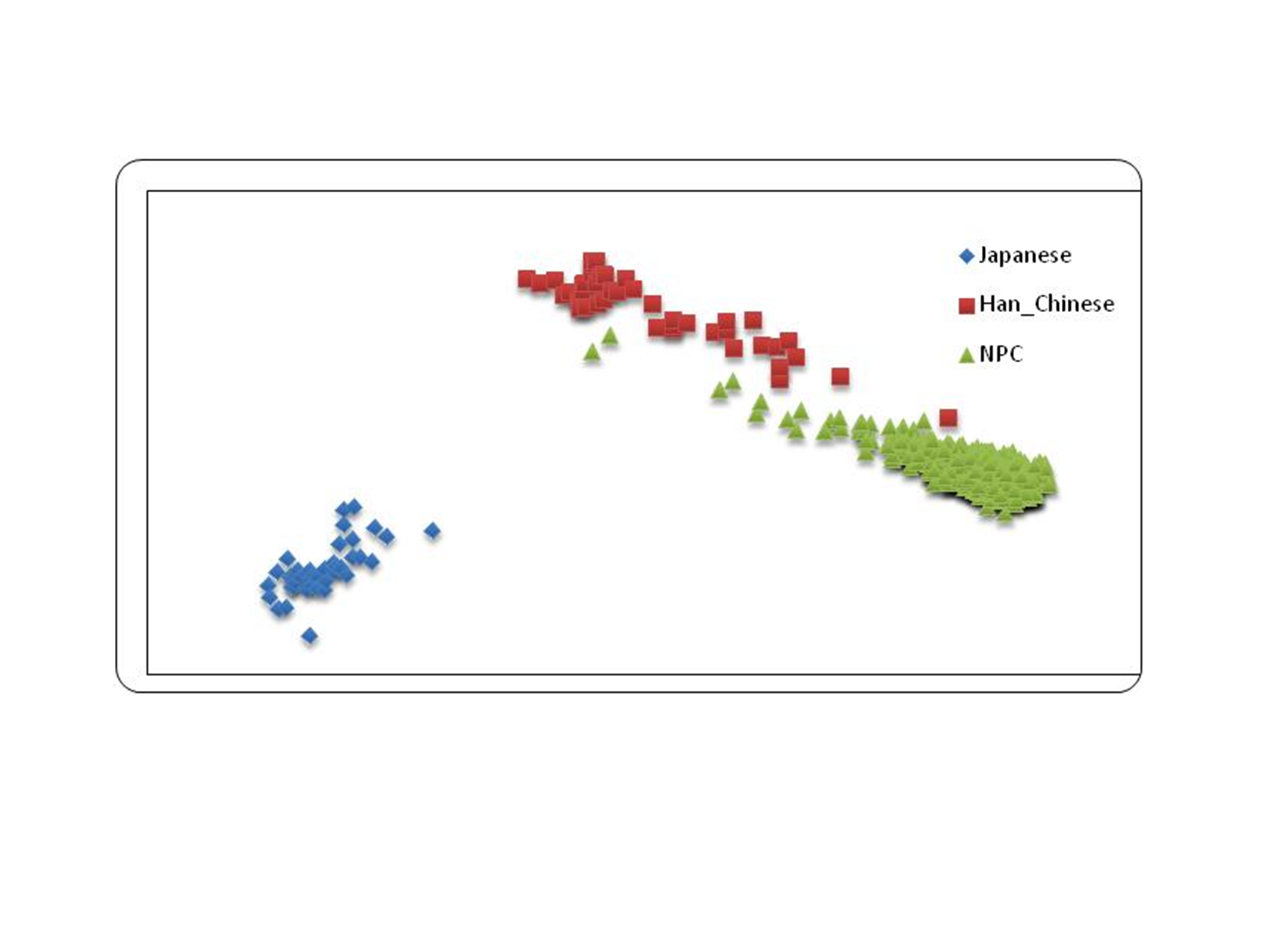

Supplement: Figure S2 — Plots of principal components from the PCA for genetic matching. Plot of the first two PCs from the PCA (N = 1043 study participants; Row I in Table S3), 44 Japanese in Tokyo, Japan (JPT), and 45 Han Chinese in Beijing, China (CHB). (TIF) [file pgen.1003103.s002.tif]

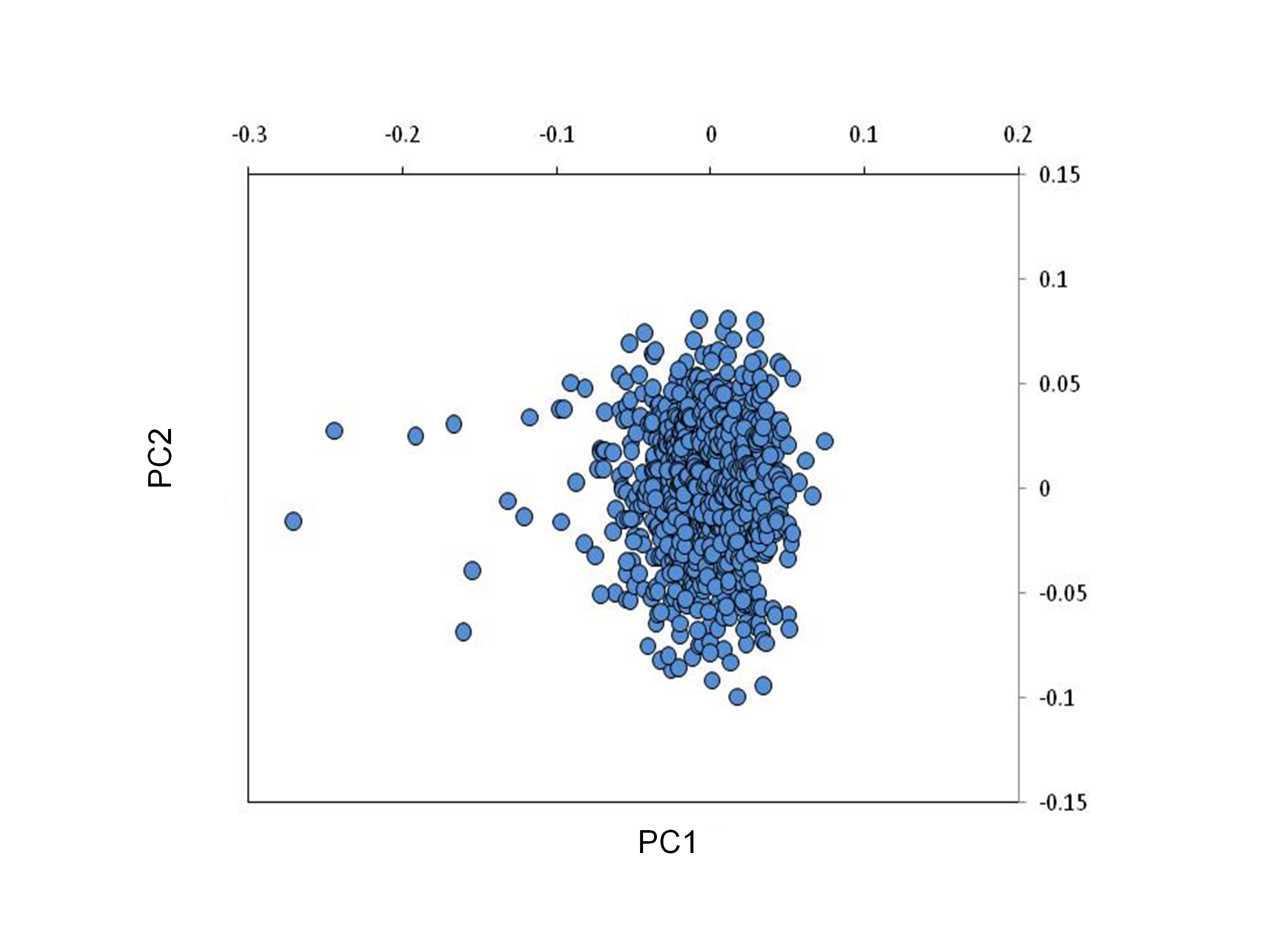

Supplement: Figure S3 — Plots of the first two principal components from the PCA of 1,043 NPC study samples for genetic matching. (TIF) [file pgen.1003103.s003.tif]

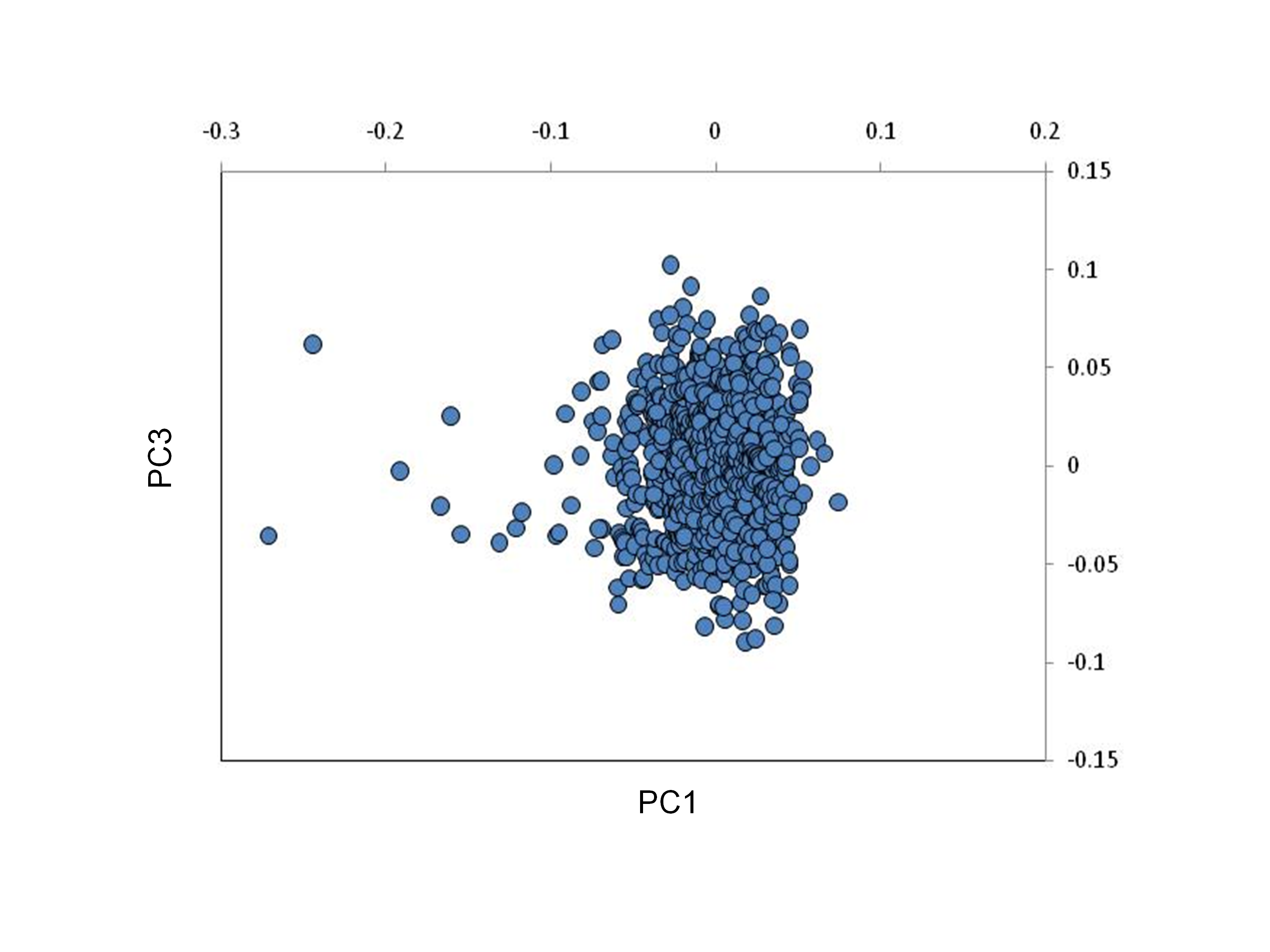

Supplement: Figure S4 — Plot of the first and third PCs from the PCA of 1,043 NPC study samples for genetic matching. (TIF) [file pgen.1003103.s004.tif]

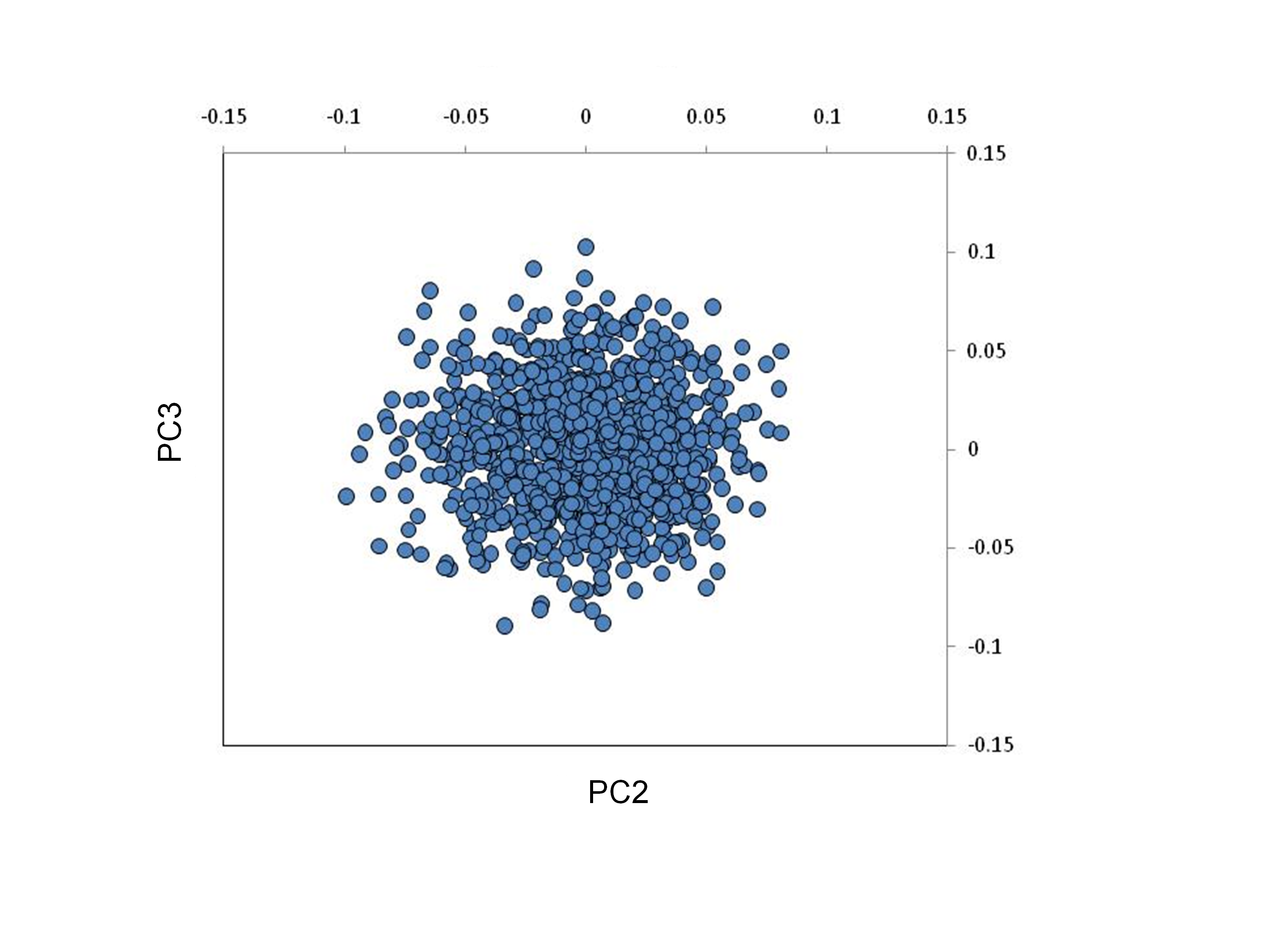

Supplement: Figure S5 — Plots of the second and third PCs from the PCA of 1,043 NPC study samples for genetic matching. (TIF) [file pgen.1003103.s005.tif]

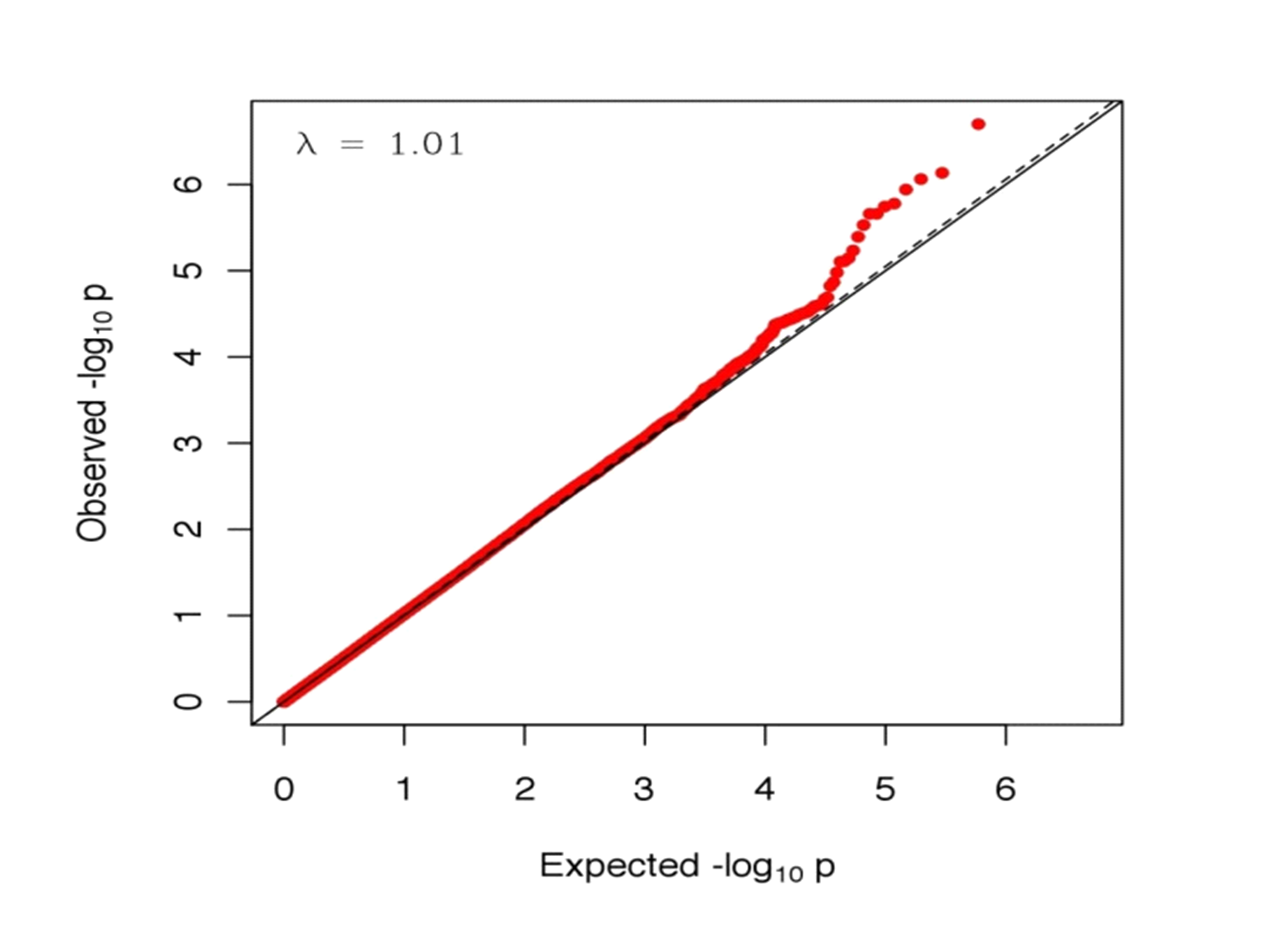

Supplement: Figure S6 — Quantile-quantile plot showing the distribution of observed statistic by allelic test for association of each SNP with NPC. (TIF) [file pgen.1003103.s006.tif]

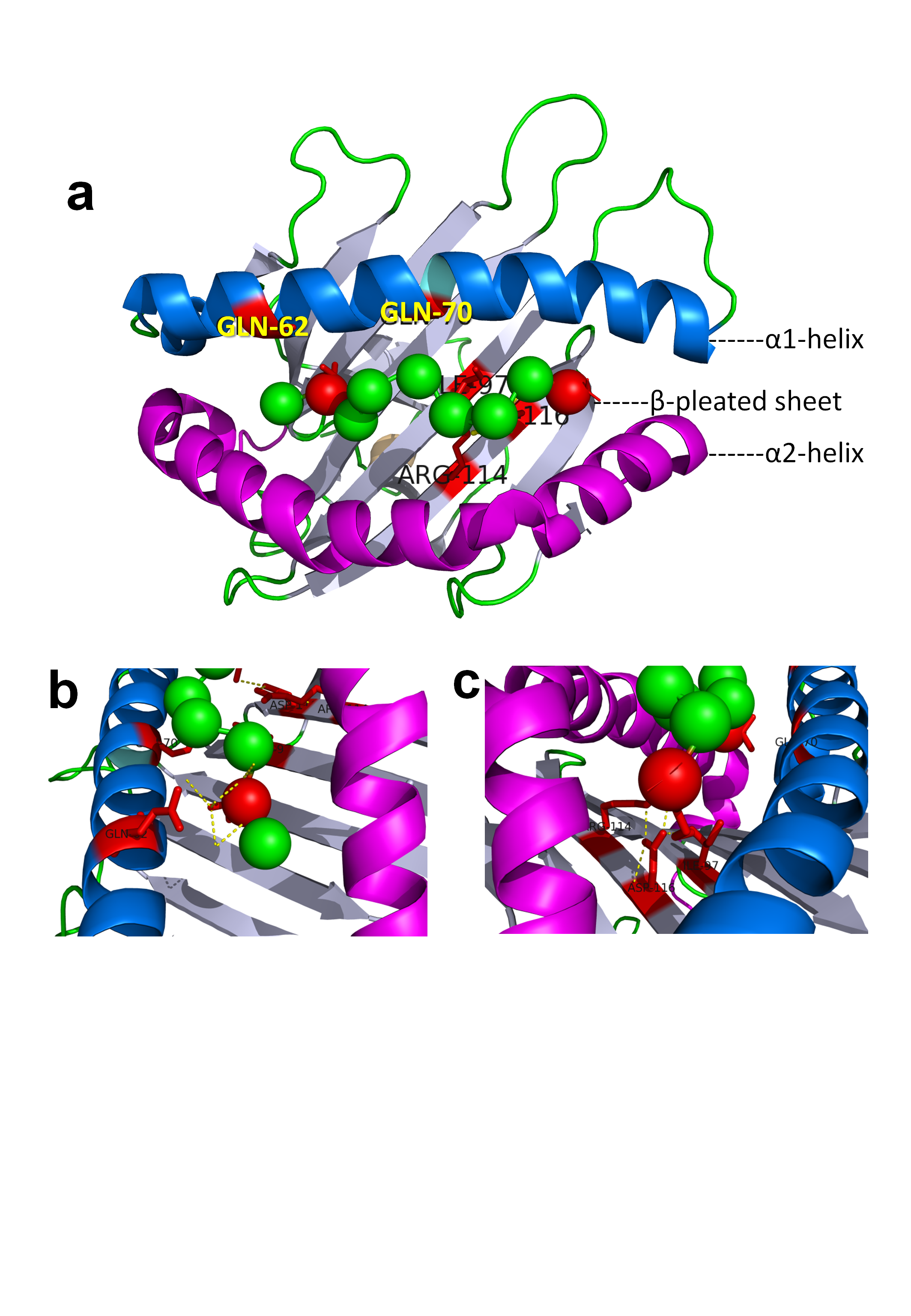

Supplement: Figure S7 — Schematic overview of the structure of HLA-A*11:01 in complex with SARS nucleocapsid peptide. The α1-helix is shown in blue; α2-helix is shown in purple; β-pleated sheet is shown in lightblue. The significantly associated exposed positons of the peptide in the binding groove are shown in red with label indicated. Green balls in the binding groove indicate SARS nucleocapsid peptide K[T]FPPTEP[K], notice the P2 and P9 residues with red. The crystal structure of 1X7Q [13] was download from PDB database (http://www.pdb.org/pdb/home/home.do). (a) Top view, (b) P2 residueThreonine (Thr, T) in peptide bingding groove, (c) P9 residue Lysine (Lys, K) in peptide binding groove. All figures were prepared with PyMOL [14]. (TIF) [file pgen.1003103.s007.tif]
