# Supplementary material for: The Principal Genetic Determinants for Nasopharyngeal Carcinoma in China Involve the HLA Class I Antigen Recognition Groove
Source: PLoS Genet. 2012 Nov 29;8(11):e1003103. doi: 10.1371/journal.pgen.1003103 (PMC3510037; doi:10.1371/journal.pgen.1003103)
Supplement: Table S1 — NPC associated classical HLA class I alleles. (DOCX) [file pgen.1003103.s008.docx]

**Table S1. NPC associated classical HLA class I alleles**

| **NPC associated alleles** | **Country of Study cohort** | **Sample size (# Cases/# Ctrls)** | **Author (year)** |
| --- | --- | --- | --- |
| (+)A*02:06, A*02:07, A*33:03, B*38:02, B*40:01, B*58:01, C*03:02  (-)A*11:01, B*13:01, B*27:04, B*55:02, B*56:01, C*04:03, C*12:02, C*12:03 | China | 356/629 | Tang (2010) [[1](#_ENREF_1)] |
| (+)A*02:07, B*38:02, B*52:01, B*58:01  (-)A*01:01, A*11:01, B*55:02, B*55:04, B*57:01 | Taiwan | 301/693 | Yu (2009) [[2](#_ENREF_2)] |
| (+)B18, B51, B57  (-)B14, Cw08 | Tunisia | 136/148 | Li (2007) [[3](#_ENREF_3)] |
| (+)Cw*0302  (-)Cw*0401 | Taiwan | 213/200 | Butsch Kovacic (2005) [[4](#_ENREF_4)] |
| (+)A2, A32, B46, B61  (-)A11, A31, B13, B27, B39, B55 | China | 247/274 | Hu (2005) [[5](#_ENREF_5)] |
| (+)A2, B38  (-)A11 | Taiwan | 67/161 | Lu (2003) [[6](#_ENREF_6)] |
| (+)A*0207, B*4601, B*5801  (-)A*1101, A*3101, B*13, B*39 | Taiwan | 366/318 | Hildesheim (2002) [[7](#_ENREF_7)] |
| (+) A28  (-) A11 Cw3 | United States | 78/137 | Burt (1996) [[8](#_ENREF_8)] |
| (+)A2, B17, B46  (-)A11, B13 | Singapore | 366/368 | Chan (1983) [[9](#_ENREF_9)] |

(+): Suscetible association

(-): Protective association

**References**

1. Tang M, Zeng Y, Poisson A, Marti D, Guan L, et al. (2010) Haplotype-dependent HLA susceptibility to nasopharyngeal carcinoma in a Southern Chinese population. Genes Immun 11: 334-342.

2. Yu KJ, Gao X, Chen CJ, Yang XR, Diehl SR, et al. (2009) Association of human leukocyte antigens with nasopharyngeal carcinoma in high-risk multiplex families in Taiwan. Hum Immunol 70: 910-914.

3. Li X, Ghandri N, Piancatelli D, Adams S, Chen D, et al. (2007) Associations between HLA class I alleles and the prevalence of nasopharyngeal carcinoma (NPC) among Tunisians. J Transl Med 5: 22.

4. Butsch Kovacic M, Martin M, Gao X, Fuksenko T, Chen CJ, et al. (2005) Variation of the killer cell immunoglobulin-like receptors and HLA-C genes in nasopharyngeal carcinoma. Cancer Epidemiol Biomarkers Prev 14: 2673-2677.

5. Hu SP, Day NE, Li DR, Luben RN, Cai KL, et al. (2005) Further evidence for an HLA-related recessive mutation in nasopharyngeal carcinoma among the Chinese. Br J Cancer 92: 967-970.

6. Lu CC, Chen JC, Jin YT, Yang HB, Chan SH, et al. (2003) Genetic susceptibility to nasopharyngeal carcinoma within the HLA-A locus in Taiwanese. Int J Cancer 103: 745-751.

7. Hildesheim A, Apple RJ, Chen CJ, Wang SS, Cheng YJ, et al. (2002) Association of HLA class I and II alleles and extended haplotypes with nasopharyngeal carcinoma in Taiwan. J Natl Cancer Inst 94: 1780-1789.

8. Burt RD, Vaughan TL, McKnight B, Davis S, Beckmann AM, et al. (1996) Associations between human leukocyte antigen type and nasopharyngeal carcinoma in Caucasians in the United States. Cancer Epidemiol Biomarkers Prev 5: 879-887.

9. Chan SH, Day NE, Kunaratnam N, Chia KB, Simons MJ (1983) HLA and nasopharyngeal carcinoma in Chinese--a further study. Int J Cancer 32: 171-176.
