# Supplementary material for: The Principal Genetic Determinants for Nasopharyngeal Carcinoma in China Involve the HLA Class I Antigen Recognition Groove
Source: PLoS Genet. 2012 Nov 29;8(11):e1003103. doi: 10.1371/journal.pgen.1003103 (PMC3510037; doi:10.1371/journal.pgen.1003103)
Supplement: Table S2 — Sample and SNP filtering for this study. (DOCX) [file pgen.1003103.s009.docx]

**Table S2. Sample and SNP filtering for this study**

|  | **Filtering** | **Dropped** | **Included** |
| --- | --- | --- | --- |
| **A)** | **Sample filtering** |  |  |
|  | Total subjects |  | 1,104 |
|  | Passed genotyping QC criteria | 28 | 1,076 |
|  | Concordant with previous genotypes | 24 | 1,052 |
|  | Concordant with clinical data (gender) | 4 | 1,048 |
|  | Cryptic relatives | 5 | 1,043 |
|  | Total used |  | 1,043 |
|  |  |  |  |
| **B)** | **SNP Filtering** |  |  |
|  | Total SNPs in SNP 6.0 |  | 934,968 |
|  | Supported SNPs | 25,346 | 909,622 |
|  | Perfect match (non-QC) | 3,022 | 906,600 |
|  | Non-redundant | 2 | 906,598 |
|  | Autosomal | 38,443 | 868,155 |
|  | <2 Mendel errors in both CEPH and NPC trios | 2,474 | 865,681 |
|  | >95% call rate | 93,597 | 772,084 |
|  | HWE P >0.001 | 9,554 | 762,530 |
|  | MAF >0.01 in NPC subject | 171,072 | 591,458 |
|  | Total used |  | 591,458 |
