# Supplementary material for: The Principal Genetic Determinants for Nasopharyngeal Carcinoma in China Involve the HLA Class I Antigen Recognition Groove
Source: PLoS Genet. 2012 Nov 29;8(11):e1003103. doi: 10.1371/journal.pgen.1003103 (PMC3510037; doi:10.1371/journal.pgen.1003103)
Supplement: Table S3 — Summary of samples used in GWAS, replication and HLA analysis. (DOCX) [file pgen.1003103.s010.docx]

**Table S3. Summary of samples used in GWAS, replication and HLA analysis**

|  |  |  |  |  |  | **EBV-IgA/VCA positive Controls** | | | **EBV-IgA/VCA negative Controls** | | | |  | |  |
| --- | --- | --- | --- | --- | --- | --- | --- | --- | --- | --- | --- | --- | --- | --- | --- |
| **Line No.** |  | **Cases** | **Mean age (s.d.)** | **Male/ female** | **Controls** | **No.** | **Mean age (s.d.)** | **Male/female** | **No.** | **Mean age (s.d.)** | **Male/female** | | **Sum** | |  |
| I | GWAS | 567 | 46.28(10.96) | 388/179 | 476 | 246 | 46.73(11.17) | 167/79 | 230 | 47.65(11.16) | | 165/65 | | 1,043 | |
| II | Replication | 356 | 50.09(10.83) | 237/119 | 629 | 287 | 45.69(8.90) | 141/146 | 342 | 46.87(10.24) | | 129/213 | | 985 | |
| III | Combined | 923 | 47.72(11.06) | 625/298 | 1,105 | 533 | 46.17(9.99) | 308/225 | 572 | 47.72(11.06) | | 294/278 | | 2,028 | |
| IV | Remaining subjects | 482 | 46.30(10.60) | 353/129 | 1545 | 755 | 45.90(11.75) | 391/364 | 790 | 46.36(11.61) | | 547/243 | | 2027 | |
| Ⅴ | HLA typing | 1,405 | 47.24(10.92) | 995/410 | 2,650 | 1,288 | 46.01(11.06) | 699/589 | 1,362 | 46.69(11.21) | | 841/521 | | 4,055 | |
