# Supplementary material for: The Principal Genetic Determinants for Nasopharyngeal Carcinoma in China Involve the HLA Class I Antigen Recognition Groove
Source: PLoS Genet. 2012 Nov 29;8(11):e1003103. doi: 10.1371/journal.pgen.1003103 (PMC3510037; doi:10.1371/journal.pgen.1003103)
Supplement: Table S4 — GWAS Results (N = 1,043 subjects) using different test combinations and genetic models for 24 SNPs with the lowest p values. (DOCX) [file pgen.1003103.s011.docx]

**Table S4. GWAS Results (*N*=1,043 subjects) using different test combinations and genetic models for 24 SNPs with the lowest p values**

| **SNP name** | **Chr.** | **bp_31** | **Gene** | **MA** | **MAF** | **Genetic Mode** | **Disease_Categories** | **P-value** | **OR(95% CI)** |
| --- | --- | --- | --- | --- | --- | --- | --- | --- | --- |
| rs12741781 | 1 | 241494775 | *SDCCAG8* | G | 0.23 | Dominant | IgA-NPC- vs. NPC+ | 4.92E-06 | 2.15(1.53-3.05) |
| rs2802723 | 1 | 241494775 | *SDCCAG8* | A | 0.28 | Dominant | [IgA+NPC- + IgA-NPC-] vs. NPC+ | 1.34E-05 | 1.73(1.34-2.23) |
| rs2694074 | 2 | 241564935 | *TSSC1* | T | 0.27 | Codominant | [IgA+NPC- + IgA-NPC-] vs. NPC+ | 1.80E-06 | 1.64(1.33-2.01) |
| rs4669243 | 2 | 3244717 | *RNF144A* | T | 0.30 | Dominant | IgA+NPC- vs. NPC+ | 1.43E-06 | 0.47(0.34-0.65) |
| rs4668564 | 2 | 8220422 | *ID2* | C | 0.30 | Dominant | IgA+NPC- vs. NPC+ | 6.23E-06 | 0.49(0.36-0.68) |
| *rs11676342* | *2* | 8226264 | *ASXL2* | *T* | *0.17* | *Dominant* | *IgA+NPC- vs. NPC+* | *4.60E-06* | *2.27(1.57-3.33)* |
| rs6774494 | 3 | 25776911 | *MECOM* | C | 0.31 | Allele | NPC+ vs. IgA+NPC- vs. IgA-NPC- | 4.39E-05 | 0.69(0.58-0.83) |
| rs6837783 | 4 | 170565327 | *C4orf33* | A | 0.34 | Recessive | NPC+ vs. IgA+NPC- vs. IgA-NPC- | 7.57E-06 | 0.46(0.33-0.65) |
| rs4713226 | 6 | 130552094 | *OR2H1* | A | 0.31 | Dominant | NPC+ vs. IgA+NPC- vs. IgA-NPC- | 1.37E-06 | 1.79(1.41-2.26) |
| rs2267633 | 6 | 29542393 | *GABBR1* | C | 0.21 | Codominant | [IgA+NPC- + IgA-NPC-] vs. NPC+ | 2.19E-06 | 0.6(0.48-0.74) |
| rs417162 | 6 | 29678820 | *HLA-A* | C | 0.31 | Codominant | [IgA+NPC- + IgA-NPC-] vs. NPC+ | 2.00E-07 | 0.6(0.49-0.73) |
| rs11977086 | 7 | 30024484 | *CACNA2D1* | C | 0.01 | Codominant | IgA+NPC- vs. NPC+ | 2.41E-06 | 0.07(0.01-0.29) |
| rs17148193 | 7 | 82141437 | *PCLO* | T | 0.21 | Allele | NPC+ vs. IgA+NPC- vs. IgA-NPC- | 2.22E-06 | 0.62(0.51-0.76) |
| rs750761 | 9 | 82727113 | *NACC2* | T | 0.38 | Dominant | IgA-NPC- vs. NPC+ | 8.40E-06 | 2.05(1.48-2.84) |
| rs9510793 | 13 | 138190468 | *TNFRSF19* | G | 0.37 | Dominant | [IgA+NPC- + IgA-NPC-] vs. NPC+ | 2.11E-05 | 1.73(1.34-2.24) |
| rs2322384 | 13 | 23111120 | *RFC3* | C | 0.17 | Dominant | IgA-NPC- vs. NPC+ | 2.64E-06 | 2.38(1.62-3.54) |
| rs4943278 | 13 | 34310895 | *RFC3* | A | 0.17 | Dominant | IgA-NPC- vs. NPC+ | 1.85E-06 | 2.41(1.64-3.58) |
| rs10146871 | 14 | 34324782 | *TMEM179* | G | 0.35 | Dominant | IgA+NPC- vs. NPC+ | 2.09E-06 | 2.09(1.52-2.87) |
| rs8049883 | 16 | 104181462 | *MT1DP* | T | 0.30 | Codominant | NPC+ vs. IgA+NPC- vs. IgA-NPC- | 2.08E-07 | 0.61(0.51-0.74) |
| rs284939 | 16 | 55230911 | *CNTNAP4* | G | 0.30 | Dominant | IgA-NPC- vs. NPC+ | 1.12E-05 | 0.49(0.36-0.69) |
| rs285001 | 16 | 75669077 | *CNTNAP4* | A | 0.30 | Dominant | IgA-NPC- vs. NPC+ | 1.11E-05 | 0.49(0.35-0.68) |
| rs10163267 | 16 | 75670065 | *MLYCD* | C | 0.36 | Allele | [IgA+NPC- + IgA-NPC-] vs. NPC+ | 5.47E-07 | 1.60(1.32-1.92) |
| rs4790142 | 17 | 82531419 | *SPATA22* | A | 0.43 | Codominant | [IgA+NPC- + IgA-NPC-] vs. NPC+ | 7.84E-06 | 1.50(1.25-1.79) |
| rs17759200 | 17 | 3318251 | *ANKFN1* | T | 0.27 | Recessive | [IgA+NPC- + IgA-NPC-] vs. NPC+ | 3.97E-06 | 3.50(1.95-6.59) |
