# Supplementary material for: The Principal Genetic Determinants for Nasopharyngeal Carcinoma in China Involve the HLA Class I Antigen Recognition Groove
Source: PLoS Genet. 2012 Nov 29;8(11):e1003103. doi: 10.1371/journal.pgen.1003103 (PMC3510037; doi:10.1371/journal.pgen.1003103)
Supplement: Table S5 — List of 16 genetic association tests. (DOCX) [file pgen.1003103.s012.docx]

**Table S5. List of 16 genetic association tests**

| **Test number** | **General method** | **Statistical test** | **Disease categories** | **Genetic mode** |
| --- | --- | --- | --- | --- |
| 1 | Categorical | FET | [IgA+NPC- + IgA-NPC-] vs. NPC+ | Codominant |
| 2 | Categorical | FET | [IgA+NPC- + IgA-NPC-] vs. NPC+ | Dominant |
| 3 | Categorical | FET | [IgA+NPC- + IgA-NPC-] vs. NPC+ | Recessive |
| 4 | Categorical | FET | IgA+NPC- vs. NPC+ | Codominant |
| 5 | Categorical | FET | IgA+NPC- vs. NPC+ | Dominant |
| 6 | Categorical | FET | IgA+NPC- vs. NPC+ | Recessive |
| 7 | Categorical | FET | IgA-NPC- vs. NPC+ | Codominant |
| 8 | Categorical | FET | IgA-NPC- vs. NPC+ | Dominant |
| 9 | Categorical | FET | IgA-NPC- vs. NPC+ | Recessive |
| 10 | Categorical | MHCST | NPC+ vs. IgA+NPC- vs. IgA-NPC- | Codominant |
| 11 | Categorical | MHCST | NPC+ vs. IgA+NPC- vs. IgA-NPC- | Dominant |
| 12 | Categorical | MHCST | NPC+ vs. IgA+NPC- vs. IgA-NPC- | Recessive |
| 13 | Categorical | FET | [IgA+NPC- + IgA-NPC-] vs. NPC+ | Allele |
| 14 | Categorical | FET | IgA+NPC- vs. NPC+ | Allele |
| 15 | Categorical | FET | IgA-NPC- vs. NPC+ | Allele |
| 16 | Categorical | MHCST | NPC+ vs. IgA+NPC- vs. IgA-NPC- | Allele |

FET: Fisher's exact test

MHCST: Mantel-Haenszel chi-square test
