# Supplementary material for: The Principal Genetic Determinants for Nasopharyngeal Carcinoma in China Involve the HLA Class I Antigen Recognition Groove
Source: PLoS Genet. 2012 Nov 29;8(11):e1003103. doi: 10.1371/journal.pgen.1003103 (PMC3510037; doi:10.1371/journal.pgen.1003103)
Supplement: Table S6 — Replication of previously reported NPC associated GWAS SNPs and candidate genes in these cohorts. (DOCX) [file pgen.1003103.s013.docx]

**Table S6. Replication of previously reported NPC associated GWAS SNPs and candidate genes in these cohorts. (*N* = 2,028 subjects line III Table S3)**

| **SNP name** | **Chr.** | **BP** | **Gene** | **MA** | **MAF** | **p- value** | **OR(95% CI)** |
| --- | --- | --- | --- | --- | --- | --- | --- |
| rs197721^a^ | 3 | 37485040 | *ITGA9* | T | 0.03/0.04 | 2.99E-01 | 0.82(1.56-1.20) |
| rs149816^a^ | 3 | 37485770 | *ITGA9* | C | 0.03/0.04 | 3.05E-01 | 0.82(1.56-1.20) |
| rs169188^a^ | 3 | 37482453 | *ITGA9* | A | 0.03/0.04 | 4.44E-01 | 0.86(0.59-1.26) |
| rs197757^a^ | 3 | 37497832 | *ITGA9* | C | 0.03/0.04 | 4.63E-01 | 0.88(0.63-1.24) |
| rs197770^a^ | 3 | 37490831 | *ITGA9* | T | 0.03/0.04 | 5.13E-01 | 0.89(0.62-1.27) |
| rs169111^a^ | 3 | 37490537 | *ITGA9* | A | 0.03/0.04 | 5.45E-01 | 0.86(0.54-1.39) |
| rs189897^a^ | 3 | 37493549 | *ITGA9* | A | 0.03/0.03 | 6.11E-01 | 0.90(0.60-1.35) |
|  |  |  |  |  |  |  |  |
| rs2517713^b^ | 6 | 30026078 | *HLA-A* | G | 0.26/0.36 | 1.92E-11 | 0.60(0.52-0.70) |
| rs9260734^b^ | 6 | 30040645 | *HCG9* | A | 0.21/0.31 | 2.63E-11 | 0.59(0.50-0.69) |
| rs5009448^b^ | 6 | 30048467 | *HCG9* | T | 0.25/0.35 | 6.40E-11 | 0.61(0.53-0.71) |
| rs29230^b^ | 6 | 29684372 | *GABBR1* | C | 0.17/0.24 | 9.48E-09 | 0.61(0.52-0.72) |
| rs3869062^b^ | 6 | 30042870 | *HCG9* | G | 0.21/0.29 | 7.03E-08 | 0.64(0.54-0.75) |
| rs29232^b^ | 6 | 29719410 | *GABBR1* | A | 0.50/0.43 | 4.35E-06 | 1.36(1.20-1.56) |
| rs16896923^b^ | 6 | 30108666 | *HCG9* | C | 0.18/0.24 | 2.57E-05 | 0.70(0.60-0.83) |
| rs3129055^b^ | 6 | 29778240 | *HLA-F* | G | 0.31/0.28 | 3.43E-02 | 1.17(1.01-1.34) |
|  |  |  |  |  |  |  |  |
| rs2517713^c,d^ | 6 | 30026078 | *HLA-A* | G | 0.26/0.36 | 1.92E-11 | 0.60(0.52-0.70) |
| rs9510793^c,e^ | 13 | 23111120 | *TNFRSF19* | C | 0.40/0.33 | 1.45E-05 | 1.34(1.17-1.53) |
| rs6774494^c,e^ | 3 | 170565327 | *MDS1-EVI1* | G | 0.28/0.34 | 5.02E-05 | 0.75(0.66-0.86) |
| rs1412829^c^ | 9 | 104181462 | *CDNK2A/2B* | G | 0.08/0.10 | 5.64E-03 | 0.72(0.57-0.91) |

a: SNPs select significant at Ng et al., 2009 [1].

b: SNPs select significant at Tse et al., 2009 [2].

c: SNPs select significant at Bei et al., 2010 [3].

d: SNPs in completely LD with rs2860580 which was reported by Bei et al., 2010 [3].

e: SNPs were included in the present GWAS SNPs array genotyping platform.

**References**

1. Ng CC, Yew PY, Puah SM, Krishnan G, Yap LF, et al. (2009) A genome-wide association study identifies ITGA9 conferring risk of nasopharyngeal carcinoma. J Hum Genet 54: 392-397.

2. Tse KP, Su WH, Chang KP, Tsang NM, Yu CJ, et al. (2009) Genome-wide association study reveals multiple nasopharyngeal carcinoma-associated loci within the HLA region at chromosome 6p21.3. Am J Hum Genet 85: 194-203.

3. Bei JX, Li Y, Jia WH, Feng BJ, Zhou G, et al. (2010) A genome-wide association study of nasopharyngeal carcinoma identifies three new susceptibility loci. Nat Genet 42: 599-603.
