# Supplementary material for: The Principal Genetic Determinants for Nasopharyngeal Carcinoma in China Involve the HLA Class I Antigen Recognition Groove
Source: PLoS Genet. 2012 Nov 29;8(11):e1003103. doi: 10.1371/journal.pgen.1003103 (PMC3510037; doi:10.1371/journal.pgen.1003103)
Supplement: Table S7 — 40 GWAS and validation of SNPs association data in two independent NPC cohorts. (DOCX) [file pgen.1003103.s014.docx]

**Table S7.** 40 GWAS and validation of SNPs association data in two independent NPC cohorts

|  |  |  |  | **GWAS (*N*=1,043)** | | | **Validation (*N*=985)** | | | **Combined (*N*=2,028)** | |
| --- | --- | --- | --- | --- | --- | --- | --- | --- | --- | --- | --- |
| **SNP name** | **Gene** | **Chr.** | **MA†** | **MAF‡** | ***P*-value** | **OR(95% CI)** | **MAF** | ***P*-value** | **OR(95% CI)** | ***P-*value** | **OR (95% CI)** |
| rs12741781 | SDCCAG8 | 1 | G | 0.27/0.19 | 6.21E-05 | 1.54(1.25-1.9) | 0.22/0.23 | 7.30E-01 | 0.96(0.76-1.21) | 2.70E-03 | 1.26(1.08-1.46) |
| rs2802723 | SDCCAG8 | 1 | T | 0.32/0.24 | 3.62E-05 | 1.52(1.24-1.85) | 0.27/0.29 | 2.89E-01 | 0.89(0.72-1.1) | 1.78E-02 | 1.18(1.03-1.36) |
| rs2694074 | TSSC1 | 2 | T | 0.31/0.21 | 2.01E-06 | 1.64(1.34-2.02) | 0.3/0.27 | 2.27E-01 | 1.14(0.92-1.4) | 3.22E-05 | 1.35(1.17-1.55) |
| rs4669243 | RNF144A | 2 | T | 0.27/0.33 | 1.16E-03 | 0.73(0.6-0.88) | 0.35/0.3 | 8.03E-03 | 1.32(1.08-1.62) | 4.89E-01 | 0.95(0.83-1.09) |
| rs4668564 | ID2 | 2 | C | 0.27/0.33 | 1.29E-03 | 0.73(0.6-0.88) | 0.35/0.3 | 1.12E-02 | 1.3(1.06-1.6) | 4.53E-01 | 0.95(0.83-1.09) |
| rs11676342 | ASXL2 | 2 | T | 0.2/0.14 | 7.29E-04 | 1.48(1.18-1.86) | 0.16/0.14 | 3.64E-01 | 1.12(0.87-1.45) | 6.02E-04 | 1.33(1.13-1.57) |
| rs169188 | ITGA9 | 3 | A | 0.03/0.03 | 7.47E-01 | 0.92(0.54-1.56) | 0.03/0.04 | 7.03E-01 | 0.9(0.52-1.56) | 4.44E-01 | 0.86(0.59-1.26) |
| rs197721 | ITGA9 | 3 | T | 0.03/0.04 | 7.89E-01 | 0.93(0.53-1.63) | 0.03/0.04 | 4.52E-01 | 0.81(0.47-1.39) | 2.99E-01 | 0.82(0.56-1.2) |
| rs149816 | ITGA9 | 3 | C | 0.03/0.04 | 3.76E-01 | 0.78(0.45-1.35) | 0.03/0.04 | 7.89E-01 | 0.93(0.54-1.6) | 3.05E-01 | 0.82(0.56-1.2) |
| rs169111 | ITGA9 | 3 | A | 0.03/0.03 | 5.08E-01 | 0.78(0.37-1.63) | 0.04/0.04 | 8.86E-01 | 1.05(0.55-1.98) | 5.45E-01 | 0.86(0.54-1.39) |
| rs197770 | ITGA9 | 3 | T | 0.04/0.04 | 8.98E-01 | 1.03(0.62-1.71) | 0.03/0.04 | 4.00E-01 | 0.79(0.46-1.37) | 5.13E-01 | 0.89(0.62-1.27) |
| rs189897 | ITGA9 | 3 | A | 0.03/0.03 | 6.22E-01 | 1.17(0.63-2.17) | 0.03/0.04 | 4.60E-01 | 0.81(0.47-1.41) | 6.11E-01 | 0.9(0.6-1.35) |
| rs197757 | ITGA9 | 3 | C | 0.03/0.03 | 8.09E-01 | 1.06(0.65-1.75) | 0.03/0.04 | 3.41E-01 | 0.79(0.48-1.29) | 4.63E-01 | 0.88(0.63-1.24) |
| rs6774494 | MECOM | 3 | G | 0.27/0.35 | 1.62E-04 | 0.7(0.58-0.84) | 0.28/0.32 | 7.73E-02 | 0.83(0.67-1.02) | 5.02E-05 | 0.75(0.66-0.86) |
| rs6837783 | C4orf33 | 4 | A | 0.3/0.39 | 5.71E-05 | 0.69(0.57-0.83) | 0.33/0.33 | 7.49E-01 | 0.97(0.79-1.18) | 2.09E-03 | 0.81(0.71-0.93) |
| rs4713226 | OR2H1 | 6 | A | 0.35/0.26 | 1.06E-05 | 1.52(1.26-1.84) | 0.35/0.3 | 3.69E-02 | 1.24(1.01-1.52) | 5.94E-06 | 1.36(1.19-1.55) |
| rs2267633 | GABBR1 | 6 | G | 0.17/0.26 | 1.43E-06 | 0.58(0.47-0.73) | 0.17/0.24 | 2.77E-04 | 0.63(0.49-0.81) | 1.89E-09 | 0.61(0.52-0.72) |
| rs29230 | GABBR1 | 6 | C | 0.17/0.25 | 2.37E-05 | 0.6(0.47-0.76) | 0.17/0.24 | 6.02E-04 | 0.65(0.5-0.83) | 9.48E-09 | 0.61(0.52-0.72) |
| rs29232 | GABBR1 | 6 | A | 0.52/0.44 | 1.71E-03 | 1.37(1.12-1.66) | 0.48/0.41 | 3.59E-03 | 1.33(1.1-1.62) | 4.35E-06 | 1.36(1.2-1.56) |
| rs3129055 | HLA-F | 6 | G | 0.35/0.3 | 3.16E-02 | 1.24(1.02-1.52) | 0.27/0.26 | 9.48E-01 | 1.01(0.81-1.26) | 3.43E-02 | 1.17(1.01-1.34) |
| rs417162 | HLA-A | 6 | C | 0.26/0.37 | 1.13E-07 | 0.58(0.48-0.71) | 0.26/0.35 | 3.75E-05 | 0.63(0.5-0.78) | 1.05E-11 | 0.61(0.52-0.7) |
| rs2517713 | HLA-A | 6 | G | 0.26/0.37 | 3.03E-07 | 0.57(0.46-0.71) | 0.26/0.35 | 2.61E-05 | 0.62(0.5-0.78) | 1.92E-11 | 0.6(0.52-0.7) |
| rs9260734 | HCG9 | 6 | A | 0.22/0.32 | 5.90E-07 | 0.57(0.45-0.71) | 0.21/0.31 | 1.32E-05 | 0.59(0.47-0.75) | 2.63E-11 | 0.59(0.5-0.69) |
| rs3869062 | HCG9 | 6 | G | 0.22/0.3 | 3.70E-05 | 0.6(0.48-0.77) | 0.2/0.28 | 1.41E-04 | 0.63(0.5-0.8) | 7.03E-08 | 0.64(0.54-0.75) |
| rs5009448 | HCG9 | 6 | T | 0.26/0.35 | 1.09E-05 | 0.63(0.51-0.77) | 0.24/0.35 | 4.43E-07 | 0.56(0.45-0.7) | 6.40E-11 | 0.61(0.53-0.71) |
| rs16896923 | HCG9 | 6 | C | 0.19/0.25 | 1.65E-03 | 0.69(0.55-0.87) | 0.17/0.23 | 9.76E-04 | 0.65(0.5-0.84) | 2.57E-05 | 0.7(0.6-0.83) |
| rs11977086 | CACNA2D1 | 7 | C | 0/0.02 | 9.28E-04 | 0.08(0.02-0.36) | 0.01/0.01 | 4.59E-01 | 1.58(0.47-5.25) | 8.58E-03 | 0.32(0.14-0.75) |
| rs17148193 | PCLO | 7 | T | 0.17/0.25 | 4.15E-05 | 0.64(0.52-0.79) | 0.19/0.21 | 8.65E-02 | 0.81(0.64-1.03) | 5.17E-05 | 0.72(0.62-0.85) |
| rs1412829 | CDNK2A/2B | 9 | G | 0.08/0.11 | 4.98E-02 | 0.73(0.53-1) | 0.07/0.1 | 3.61E-02 | 0.68(0.48-0.98) | 5.64E-03 | 0.72(0.57-0.91) |
| rs750761 | NACC2 | 9 | A | 0.41/0.34 | 3.58E-03 | 1.31(1.09-1.57) | 0.34/0.36 | 3.49E-01 | 0.91(0.75-1.11) | 7.26E-02 | 1.13(0.99-1.28) |
| rs9510793 | TNFRSF19 | 13 | C | 0.41/0.32 | 6.00E-05 | 1.45(1.21-1.73) | 0.38/0.34 | 7.75E-02 | 1.2(0.98-1.47) | 1.45E-05 | 1.34(1.17-1.53) |
| rs2322384 | RFC3 | 13 | C | 0.19/0.14 | 2.86E-03 | 1.45(1.14-1.84) | 0.15/0.18 | 1.14E-02 | 0.71(0.54-0.93) | 7.25E-01 | 1.03(0.87-1.22) |
| rs4943278 | RFC3 | 13 | T | 0.19/0.14 | 2.01E-03 | 1.46(1.15-1.86) | 0.14/0.18 | 1.28E-02 | 0.71(0.55-0.93) | 5.77E-01 | 1.05(0.89-1.24) |
| rs10146871 | TMEM179 | 14 | C | 0.39/0.31 | 7.38E-05 | 1.44(1.2-1.73) | 0.37/0.33 | 2.34E-02 | 1.27(1.03-1.55) | 1.03E-05 | 1.35(1.18-1.54) |
| rs8049883 | MT1DP | 16 | A | 0.26/0.36 | 9.49E-07 | 0.61(0.5-0.74) | 0.3/0.26 | 1.19E-02 | 1.31(1.06-1.61) | 1.04E-01 | 0.89(0.77-1.02) |
| rs284939 | CNTNAP4 | 16 | G | 0.27/0.32 | 1.61E-02 | 0.79(0.65-0.96) | 0.29/0.31 | 2.10E-01 | 0.87(0.71-1.08) | 3.29E-03 | 0.81(0.71-0.93) |
| rs285001 | CNTNAP4 | 16 | T | 0.27/0.32 | 1.31E-02 | 0.79(0.65-0.95) | 0.29/0.31 | 2.44E-01 | 0.88(0.72-1.09) | 3.52E-03 | 0.81(0.71-0.93) |
| rs10163267 | MLYCD | 16 | C | 0.41/0.3 | 1.17E-06 | 1.57(1.31-1.88) | 0.34/0.35 | 8.25E-01 | 0.98(0.79-1.21) | 5.07E-04 | 1.27(1.11-1.44) |
| rs4790142 | SPATA22 | 17 | A | 0.47/0.37 | 7.60E-06 | 1.51(1.26-1.8) | 0.45/0.48 | 2.12E-01 | 0.89(0.73-1.07) | 4.77E-02 | 1.14(1-1.29) |
| rs17759200 | ANKFN1 | 17 | A | 0.29/0.26 | 1.67E-01 | 1.15(0.94-1.4) | 0.27/0.25 | 3.29E-01 | 1.11(0.9-1.39) | 2.25E-02 | 1.18(1.02-1.36) |

†:MA, Minor allele;

‡: MAF, Minor allele frequencies
