# Supplementary material for: The Principal Genetic Determinants for Nasopharyngeal Carcinoma in China Involve the HLA Class I Antigen Recognition Groove
Source: PLoS Genet. 2012 Nov 29;8(11):e1003103. doi: 10.1371/journal.pgen.1003103 (PMC3510037; doi:10.1371/journal.pgen.1003103)
Supplement: Table S8 — Significant explicit and imputed haplotype NPC association analysis. (DOCX) [file pgen.1003103.s015.docx]

**Table S8. Significant explicit and imputed haplotype NPC association analysis (*N* = 4,055)#**

|  | **Haplotype frequencies** | | | **NPC vs. controls** | | **NPC VS. E.P. Controls** | | **NPC VS. E.N. Controls** | |
| --- | --- | --- | --- | --- | --- | --- | --- | --- | --- |
| **Haplotype** | **NPC cases**  **(*N* = 1405)** | **E.P. Controls***  **(*N* = 1288)** | **E.N. Controls†**  **(*N* = 1362)** | **OR (95% CI)** | ***p*-value** | **OR (95% CI)** | ***p*-value** | **OR (95% CI)** | ***p*-value** |
| Susceptible effect |  |  |  |  |  |  |  |  |  |
| *A*02:03-B*38:02-C*07:02* | 17.30(243) | 8.23(106) | 12.56(171) | 1.63(1.39-1.92) | 3.33E-09 | 2.04(1.64-2.54) | 1.46E-10 |  | Ns |
| *A*02:03-B*38:02* | 17.51(246) | 8.23(106) | 12.63(172) | 1.74(1.46-2.08) | 9.63E-10 | 2.19(1.74-2.77) | 4.71E-11 |  | Ns |
| *A*02:03- C*07:02* | 21.07(296) | 11.49(148) | 16.59(226) | 1.58(1.35-1.86) | 1.59E-08 | 1.90(1.55-2.33) | 6.26E-10 |  | Ns |
| *B*38:02-C*07:02* | 22.49(316) | 12.73(164) | 15.79(215) | 1.67(1.43-1.95) | 1.18E-10 | 1.88(1.54-2.29) | 3.45E-10 | 1.51(1.26-1.81) | 8.12E-06 |
| *A*02:07-B*46:01-C*01:02* | 23.99(337) | 20.11(259) | 14.98(204) | 1.40(1.22-1.61) | 1.40E-06 |  | Ns | 1.63(1.34-1.93) | 1.44E-08 |
| *A*02:07-B*46:01* | 24.70(347) | 20.42(263) | 16.01(218) | 1.45(1.26-1.68) | 3.58E-07 |  | Ns | 1.64(1.38-1.95) | 2.32E-08 |
| *A*02:07- C*01:02* | 25.20(354) | 20.96(270) | 16.15(220) | 1.46(1.27-1.68) | 1.67E-07 |  | Ns | 1.66(1.40-1.96) | 7.52E-09 |
| *B*46:01-C*01:02* | 33.31(468) | 32.22(415) | 24.67(336) |  | Ns |  | Ns | 1.44(1.28-1.66) | 6.84E-07 |
| *A*33:03-B*58:01-C*03:02* | 24.77(348) | 23.14(298) | 16.81(229) |  | Ns |  | Ns | 1.57(1.31-1.87) | 7.20E-07 |
| *A*33:03-B*58:01* | 27.33(384) | 24.61(317) | 18.65(254) | 1.32(1.15-1.51) | 1.08E-04 |  | Ns | 1.59(1.34-1.89) | 1.07E-07 |
| *A*33:03- C*03:02* | 27.54(387) | 24.61(317) | 18.80(256) | 1.32(1.15-1.52) | 1.04E-04 |  | Ns | 1.58(1.33-1.88) | 1.48E-07 |
| *B*58:01-C*03:02* | 30.53(429) | 27.95(360) | 22.32(304) |  |  |  | Ns | 1.47(1.25-1.72) | 2.03E-06 |
|  |  |  |  |  |  |  |  |  |  |
| Protective effect |  |  |  |  |  |  |  |  |  |
| *A*11:01-B*13:01-C*03:04* | 7.54(106) | 14.75(190) | 11.23(153) | 0.55(0.44-0.67) | 9.28E-08 | 0.47(0.37-0.61) | 2.66E-09 |  | Ns |
| *A*11:01-B*13:01* | 7.90(111) | 14.91(192) | 12.19(166) | 0.54(0.43-0.68) | 5.62E-08 | 0.48(0.37-0.61) | 5.34E-09 |  | Ns |
| *A*11:01-C*03:04* | 11.46(161) | 18.01(232) | 16.67(227) | 0.61(0.50-0.74) | 2.87E-07 | 0.57(0.46-0.71) | 3.50E-07 | 0.65(0.52-0.80) | 6.34E-05 |
| *B*13:01-C*03:04* | 14.59(205) | 22.20(286) | 19.46(265) | 0.66(0.56-0.78) | 8.97E-07 | 0.61(0.50-0.73) | 2.19E-07 |  | Ns |
| *A*11:01-B*46:01-C*01:02* | 2.28(32) | 5.20(67) | 4.33(59) |  | Ns | 0.42(0.28-0.64) | 5.05E-05 |  | Ns |
| *A*11:01-B*46:01* | 2.49(35) | 6.13(79) | 5.43(74) | 0.42(0.29-0.61) | 4.36E-06 | 0.39(0.26-0.59) | 5.06E-06 |  | Ns |
| *A*11:01-C*01:02* | 3.77(53) | 8.54(110) | 7.64(104) | 0.47(0.35-0.64) | 1.06E-06 | 0.44(0.31-0.61) | 8.70E-07 | 0.52(0.37-0.72) | 1.06E-04 |
| *A*02:03-B*55:02* | 0.85(12) | 2.95(38) | 3.01(41) | 0.28(0.15-0.51) | 4.09E-05 |  | Ns | 0.27(0.14-0.52) | 9.18E-05 |
| *B*55:02-C*12:03* | 0.50(7) | 2.79(36) | 2.13(29) | 0.20(0.09-0.44) | 6.27E-05 | 0.18(0.08-0.41) | 3.96E-05 |  | Ns |
| *B*27:04-C*12:02* | 0.85(12) | 1.48(19) | 2.94(40) |  | Ns |  | Ns | 0.28(0.15-0.54) | 1.51E-04 |

*: E. P. controls, the controls of NPC free and EBV IgA/VCA antibody positive

†: E. N. controls, the controls of NPC free and EBV IgA/VCA antibody negative

# See Materials and Methods
