# Supplementary material for: The Principal Genetic Determinants for Nasopharyngeal Carcinoma in China Involve the HLA Class I Antigen Recognition Groove
Source: PLoS Genet. 2012 Nov 29;8(11):e1003103. doi: 10.1371/journal.pgen.1003103 (PMC3510037; doi:10.1371/journal.pgen.1003103)
Supplement: Table S9 — Association results for the amino acid residues in each of the classical HLA loci in all study subjects. (DOCX) [file pgen.1003103.s016.docx]

**Table S9. Association results for the amino acid residues in each of the classical HLA loci in all study subjects**

| **Locus** | **Amino acid position** | **A1*** | **A2** | **Frequency in controls** | **Frequency in cases** | **OR** | **P-value** | **Omnibus P-value** | **Represent Alleles** |
| --- | --- | --- | --- | --- | --- | --- | --- | --- | --- |
| A | -22 | I | V | 0.0011 | 0.0004 | 0.31 | 2.58E-01 |  | *A*34:01* |
| A | -15 | L | V | 0.5227 | 0.4404 | 0.72 | 1.91E-12 |  | *A*01:01, A*03:01, A*03:02, A*11:01, A*11:02, A*11:04, A*29:01, A*29:02, A*30:01, A*30:02, A*31:01, A*32:01, A*33:03, A*74:02* |
| A | -11 | L | S | 0.1624 | 0.1808 | 1.14 | 3.52E-02 |  | *A*01:01, A*02:01, A*02:03, A*02:05, A*02:06, A*02:07, A*02:10, A*02:11, A*03:01, A*03:02, A*11:01, A*11:02, A*11:04, A*23:01, A*24:02, A*24:03, A*24:04, A*24:07, A*25:01, A*26:01, A*30:01, A*30:02, A*34:01, A*66:01, A*68:01, A*68:02, A*69:01* |
| A | 3 | Q | H | 0.0004 | 0.0000 | 0.00 | 3.03E-01 |  | *A*24:20* |
| A | 9 | F | - | 0.3132 | 0.3658 | 1.27 | 1.66E-06 | 7.50E-11 | *A*01:01, A*01:03, A*02:01, A*02:03, A*02:07, A*02:11, A*03:01, A*03:02, A*32:01, A*32:03, A*74:02* |
| A | 9 | S | - | 0.1545 | 0.1491 | 0.96 | 5.19E-01 |  | *A*23:01, A*24:02, A*24:03, A*24:04, A*24:05, A*24:07, A*24:10, A*24:20, A*24:21, A*24:64, A*24:68, A*30:01, A*30:02* |
| A | 9 | Y | - | 0.3777 | 0.3053 | 0.72 | 8.40E-11 |  | *A*02:05, A*02:06, A*02:10, A*11:01, A*11:02, A*11:04, A*25:01, A*26:01, A*34:01, A*66:01, A*68:01, A*68:02, A*69:01* |
| A | 9 | T | - | 0.1545 | 0.1797 | 1.20 | 3.50E-03 |  | *A*29:01, A*29:02, A*31:01, A*33:03* |
| A | 12 | M | V | 0.0004 | 0.0000 | 0.00 | 3.03E-01 |  | *A*68:02* |
| A | 17 | S | R | 0.0072 | 0.0043 | 0.59 | 1.13E-01 |  | *A*30:01, A*30:02* |
| A | 19 | K | E | 0.0408 | 0.0445 | 1.10 | 4.26E-01 |  | *A*11:02* |
| A | 43 | R | Q | 0.0000 | 0.0004 | NA | 1.70E-01 |  | *A*02:05* |
| A | 44 | K | R | 0.0066 | 0.0039 | 0.59 | 1.25E-01 |  | *A*01:01, A*01:03* |
| A | 56 | R | G | 0.0145 | 0.0064 | 0.44 | 1.22E-03 |  | *A*30:01, A*30:02, A*31:01* |
| A | 62 | Q | - | 0.3753 | 0.2623 | 0.59 | 1.17E-24 | 7.38E-29 | *A*01:01, A*01:03, A*03:01, A*03:02, A*11:01, A*11:02, A*11:04, A*30:01, A*30:02, A*31:01, A*32:01, A*32:03, A*74:02* |
| A | 62 | G | - | 0.3157 | 0.3993 | 1.44 | 4.68E-14 |  | *A*02:01, A*02:03, A*02:05, A*02:06, A*02:07, A*02:10, A*02:11* |
| A | 62 | E | - | 0.1474 | 0.1448 | 0.98 | 7.60E-01 |  | *A*23:01, A*24:02, A*24:03, A*24:04, A*24:05, A*24:07, A*24:10, A*24:20, A*24:21, A*24:64, A*24:68* |
| A | 62 | R | - | 0.1547 | 0.1929 | 1.31 | 1.21E-05 |  | *A*25:01, A*26:01, A*33:03, A*34:01, A*66:01, A*68:01, A*68:02, A*69:01* |
| A | 62 | L | - | 0.0070 | 0.0007 | 0.10 | 1.03E-04 |  | *A*29:01, A*29:02* |
| A | 63 | E | - | 0.1617 | 0.1936 | 1.25 | 3.00E-04 | 6.19E-08 | *A*01:01, A*01:03, A*02:01, A*02:03, A*02:05, A*02:06, A*02:07, A*02:10, A*02:11, A*03:01, A*03:02, A*11:01, A*11:02, A*11:04, A*23:01, A*24:02, A*24:03, A*24:04, A*24:05, A*24:07, A*24:10, A*24:20, A*24:21, A*24:64, A*24:68, A*30:01, A*30:02, A*31:01, A*32:01, A*32:03, A*74:02* |
| A | 63 | N | - | 0.1547 | 0.1929 | 1.31 | 1.21E-05 |  | *A*25:01, A*26:01, A*33:03, A*34:01, A*66:01, A*68:01, A*68:02, A*69:01* |
| A | 63 | Q | - | 0.0070 | 0.0007 | 0.10 | 1.03E-04 |  | *A*29:01, A*29:02* |
| A | 65 | G | R | 0.1474 | 0.1448 | 0.98 | 7.60E-01 |  | *A*23:01, A*24:02, A*24:03, A*24:04, A*24:05, A*24:07, A*24:10, A*24:20, A*24:21, A*24:64, A*24:68* |
| A | 66 | K | N | 0.4642 | 0.5445 | 1.38 | 5.74E-12 |  | *A*02:01, A*02:03, A*02:05, A*02:06, A*02:07, A*02:10, A*02:11, A*23:01, A*24:02, A*24:03, A*24:04, A*24:05, A*24:07, A*24:10, A*24:20, A*24:21, A*24:64, A*24:68, A*34:01* |
| A | 67 | M | V | 0.0066 | 0.0039 | 0.59 | 1.25E-01 |  | *A*01:01, A*01:03* |
| A | 70 | Q | H | 0.3670 | 0.2623 | 0.61 | 1.47E-21 |  | *A*03:01, A*03:02, A*11:01, A*11:02, A*11:04, A*24:07, A*29:01, A*29:02, A*30:01, A*34:01, A*66:01, A*68:01, A*68:02, A*69:01* |
| A | 73 | I | T | 0.1477 | 0.1790 | 1.26 | 2.44E-04 |  | *A*02:11, A*31:01, A*33:03* |
| A | 74 | H | D | 0.3155 | 0.3993 | 1.44 | 4.08E-14 |  | *A*02:01, A*02:03, A*02:05, A*02:06, A*02:07, A*02:10* |
| A | 76 | A | - | 0.0255 | 0.0199 | 0.78 | 1.17E-01 | 2.72E-01 | *A*01:01, A*01:03, A*24:04, A*26:01, A*29:01, A*29:02* |
| A | 76 | E | - | 0.1483 | 0.1459 | 0.98 | 7.72E-01 |  | *A*23:01, A*24:02, A*24:03, A*24:05, A*24:07, A*24:10, A*24:20, A*24:21, A*24:64, A*24:68, A*25:01, A*30:02, A*32:01, A*32:03* |
| A | 76 | V | - | 0.1738 | 0.1658 | 0.95 | 3.66E-01 |  | *A*02:01, A*02:03, A*02:05, A*02:06, A*02:07, A*02:10, A*02:11, A*03:01, A*03:02, A*11:01, A*11:02, A*11:04, A*30:01, A*31:01, A*33:03, A*34:01, A*66:01, A*68:01, A*68:02, A*69:01, A*74:02* |
| A | 77 | N | - | 0.1730 | 0.1648 | 0.94 | 3.47E-01 | 5.82E-01 | *A*01:01, A*01:03, A*23:01, A*24:02, A*24:03, A*24:04, A*24:05, A*24:07, A*24:10, A*24:20, A*24:21, A*24:64, A*24:68, A*26:01, A*29:01, A*29:02, A*30:02, A*32:03* |
| A | 77 | D | - | 0.1738 | 0.1658 | 0.95 | 3.66E-01 |  | *A*02:01, A*02:03, A*02:05, A*02:06, A*02:07, A*02:10, A*02:11, A*03:01, A*03:02, A*11:01, A*11:02, A*11:04, A*30:01, A*31:01, A*33:03, A*34:01, A*66:01, A*68:01, A*68:02, A*69:01, A*74:02* |
| A | 77 | S | - | 0.0008 | 0.0011 | 1.42 | 6.48E-01 |  | *A*25:01, A*32:01* |
| A | 79 | R | G | 0.1481 | 0.1459 | 0.98 | 7.90E-01 |  | *A*23:01, A*24:02, A*24:03, A*24:05, A*24:07, A*24:10, A*24:20, A*24:21, A*24:64, A*24:68, A*25:01, A*32:01, A*32:03* |
| A | 80 | I | T | 0.1481 | 0.1459 | 0.98 | 7.90E-01 |  | *A*23:01, A*24:02, A*24:03, A*24:05, A*24:07, A*24:10, A*24:20, A*24:21, A*24:64, A*24:68, A*25:01, A*32:01, A*32:03* |
| A | 81 | A | L | 0.1481 | 0.1459 | 0.98 | 7.90E-01 |  | *A*23:01, A*24:02, A*24:03, A*24:05, A*24:07, A*24:10, A*24:20, A*24:21, A*24:64, A*24:68, A*25:01, A*32:01, A*32:03* |
| A | 82 | L | R | 0.1481 | 0.1459 | 0.98 | 7.90E-01 |  | *A*23:01, A*24:02, A*24:03, A*24:05, A*24:07, A*24:10, A*24:20, A*24:21, A*24:64, A*24:68, A*25:01, A*32:01, A*32:03* |
| A | 83 | R | G | 0.1481 | 0.1459 | 0.98 | 7.90E-01 |  | *A*23:01, A*24:02, A*24:03, A*24:05, A*24:07, A*24:10, A*24:20, A*24:21, A*24:64, A*24:68, A*25:01, A*32:01, A*32:03* |
| A | 90 | D | A | 0.3564 | 0.2669 | 0.66 | 2.68E-16 |  | *A*01:01, A*01:03, A*11:01, A*11:02, A*11:04, A*25:01, A*26:01, A*34:01, A*66:01* |
| A | 170 | S | R | 0.0000 | 0.0004 | NA | 1.70E-01 |  | *A*24:68* |
| A | 95 | I | - | 0.4632 | 0.5441 | 1.38 | 4.02E-12 | 7.38E-14 | *A*01:01, A*01:03, A*03:01, A*03:02, A*11:01, A*11:02, A*11:04, A*25:01, A*26:01, A*29:01, A*29:02, A*30:01, A*30:02, A*31:01, A*32:01, A*32:03, A*33:03, A*34:01, A*66:01, A*68:01, A*68:02, A*74:02* |
| A | 95 | V | - | 0.3158 | 0.3989 | 1.44 | 6.81E-14 |  | *A*02:01, A*02:03, A*02:06, A*02:07, A*02:10, A*02:11, A*69:01* |
| A | 95 | L | - | 0.1474 | 0.1452 | 0.98 | 7.93E-01 |  | *A*02:05, A*23:01, A*24:02, A*24:03, A*24:04, A*24:05, A*24:07, A*24:10, A*24:20, A*24:21, A*24:64, A*24:68* |
| A | 97 | I | - | 0.3598 | 0.2591 | 0.62 | 3.05E-20 | 3.59E-21 | *A*01:01, A*03:01, A*03:02, A*11:01, A*11:02, A*11:04, A*30:01, A*30:02* |
| A | 97 | M | - | 0.3109 | 0.3253 | 1.07 | 1.87E-01 |  | *A*01:03, A*23:01, A*24:02, A*24:03, A*24:04, A*24:05, A*24:07, A*24:10, A*24:20, A*24:21, A*24:68, A*29:01, A*29:02, A*31:01, A*32:01, A*32:03, A*33:03, A*68:01, A*74:02* |
| A | 97 | R | - | 0.3292 | 0.4153 | 1.45 | 1.50E-14 |  | *A*02:01, A*02:03, A*02:05, A*02:06, A*02:07, A*02:10, A*02:11, A*25:01, A*26:01, A*34:01, A*66:01, A*68:02, A*69:01* |
| A | 97 | W | - | 0.0000 | 0.0004 | NA | 1.70E-01 |  | *A*24:64* |
| A | 99 | Y | - | 0.2717 | 0.3096 | 1.20 | 3.19E-04 | 4.38E-06 | *A*01:01, A*01:03, A*02:01, A*02:03, A*02:05, A*02:06, A*02:11, A*03:01, A*03:02, A*11:01, A*11:02, A*11:04, A*25:01, A*26:01, A*29:01, A*29:02, A*30:01, A*30:02, A*31:01, A*32:01, A*32:03, A*33:03, A*34:01, A*66:01, A*68:01, A*68:02, A*69:01, A*74:02* |
| A | 99 | C | - | 0.1242 | 0.1648 | 1.39 | 4.58E-07 |  | *A*02:07* |
| A | 99 | F | - | 0.1475 | 0.1445 | 0.98 | 7.11E-01 |  | *A*02:10, A*23:01, A*24:02, A*24:03, A*24:04, A*24:05, A*24:07, A*24:10, A*24:20, A*24:21, A*24:68* |
| A | 99 | I | - | 0.0000 | 0.0004 | NA | 1.70E-01 |  | *A*24:64* |
| A | 102 | H | D | 0.0066 | 0.0007 | 0.11 | 1.79E-04 |  | *A*29:01* |
| A | 105 | P | S | 0.3647 | 0.2680 | 0.64 | 1.26E-18 |  | *A*01:01, A*01:03, A*11:01, A*11:02, A*11:04, A*25:01, A*26:01, A*32:01, A*32:03, A*34:01, A*66:01, A*68:02, A*74:02* |
| A | 107 | W | G | 0.3157 | 0.3993 | 1.44 | 4.68E-14 |  | *A*02:01, A*02:03, A*02:05, A*02:06, A*02:07, A*02:11, A*69:01* |
| A | 109 | L | F | 0.0079 | 0.0011 | 0.13 | 7.63E-05 |  | *A*32:01, A*32:03, A*74:02* |
| A | 114 | R | - | 0.3608 | 0.2555 | 0.61 | 5.74E-22 | 5.88E-21 | *A*01:01, A*01:03, A*03:01, A*03:02, A*11:01, A*11:02, A*11:04, A*29:01, A*29:02, A*68:01* |
| A | 114 | H | - | 0.4636 | 0.5441 | 1.38 | 5.06E-12 |  | *A*02:01, A*02:03, A*02:05, A*02:06, A*02:07, A*02:10, A*02:11, A*23:01, A*24:02, A*24:03, A*24:04, A*24:05, A*24:07, A*24:10, A*24:20, A*24:21, A*24:64, A*24:68, A*68:02, A*69:01* |
| A | 114 | Q | - | 0.1685 | 0.1961 | 1.20 | 1.99E-03 |  | *A*25:01, A*26:01, A*31:01, A*32:01, A*32:03, A*33:03, A*34:01, A*66:01, A*74:02* |
| A | 114 | E | - | 0.0072 | 0.0043 | 0.59 | 1.13E-01 |  | *A*30:01, A*30:02* |
| A | 116 | D | - | 0.4708 | 0.5484 | 1.37 | 2.84E-11 | 2.56E-11 | *A*01:01, A*01:03, A*03:01, A*03:02, A*11:01, A*11:02, A*11:04, A*25:01, A*26:01, A*29:01, A*29:02, A*31:01, A*32:01, A*32:03, A*33:03, A*34:01, A*66:01, A*68:01, A*74:02* |
| A | 116 | Y | - | 0.4636 | 0.5441 | 1.38 | 5.06E-12 |  | *A*02:01, A*02:03, A*02:05, A*02:06, A*02:07, A*02:10, A*02:11, A*23:01, A*24:02, A*24:03, A*24:04, A*24:05, A*24:07, A*24:10, A*24:20, A*24:21, A*24:64, A*24:68, A*68:02, A*69:01* |
| A | 116 | H | - | 0.0072 | 0.0043 | 0.59 | 1.13E-01 |  | *A*30:01, A*30:02* |
| A | 127 | K | N | 0.4643 | 0.5441 | 1.38 | 7.96E-12 |  | *A*02:01, A*02:03, A*02:05, A*02:06, A*02:07, A*02:10, A*02:11, A*23:01, A*24:02, A*24:03, A*24:04, A*24:05, A*24:07, A*24:10, A*24:20, A*24:64, A*24:68, A*68:01, A*68:02, A*69:01* |
| A | 142 | T | I | 0.3172 | 0.3993 | 1.43 | 1.37E-13 |  | *A*02:01, A*02:03, A*02:05, A*02:06, A*02:07, A*02:10, A*02:11, A*68:01, A*68:02, A*69:01* |
| A | 144 | Q | K | 0.1834 | 0.2025 | 1.13 | 3.70E-02 |  | *A*23:01, A*24:05, A*25:01, A*26:01, A*29:01, A*29:02, A*30:01, A*30:02, A*31:01, A*32:01, A*32:03, A*33:03, A*34:01, A*66:01, A*74:02* |
| A | 145 | H | R | 0.3172 | 0.3993 | 1.43 | 1.37E-13 |  | *A*02:01, A*02:03, A*02:05, A*02:06, A*02:07, A*02:10, A*02:11, A*68:01, A*68:02, A*69:01* |
| A | 149 | T | A | 0.1430 | 0.1737 | 1.26 | 2.71E-04 |  | *A*02:03, A*25:01, A*26:01, A*34:01, A*66:01* |
| A | 150 | V | A | 0.0066 | 0.0039 | 0.59 | 1.25E-01 |  | *A*01:01, A*01:03* |
| A | 151 | R | H | 0.1704 | 0.1861 | 1.11 | 7.64E-02 |  | *A*23:01, A*29:01, A*29:02, A*30:01, A*30:02, A*31:01, A*32:01, A*32:03, A*33:03, A*74:02* |
| A | 152 | A | - | 0.3434 | 0.2509 | 0.64 | 1.12E-17 | 4.98E-16 | *A*01:01, A*01:03, A*11:01, A*11:02, A*11:04* |
| A | 152 | R | - | 0.0002 | 0.0000 | 0.00 | 4.67E-01 |  | *A*30:02* |
| A | 152 | V | - | 0.5028 | 0.4327 | 0.75 | 1.83E-09 |  | *A*02:01, A*02:05, A*02:06, A*02:07, A*02:10, A*02:11, A*03:02, A*23:01, A*24:02, A*24:03, A*24:04, A*24:05, A*24:07, A*24:10, A*24:20, A*24:21, A*24:64, A*24:68, A*29:01, A*29:02, A*31:01, A*32:01, A*32:03, A*33:03, A*68:01, A*68:02, A*69:01, A*74:02* |
| A | 152 | E | - | 0.1523 | 0.1776 | 1.20 | 3.16E-03 |  | *A*02:03, A*03:01, A*25:01, A*26:01, A*34:01, A*66:01* |
| A | 152 | W | - | 0.0070 | 0.0043 | 0.61 | 1.34E-01 |  | *A*30:01* |
| A | 156 | R | - | 0.0066 | 0.0039 | 0.59 | 1.25E-01 | 9.53E-15 | *A*01:01, A*01:03* |
| A | 156 | L | - | 0.3655 | 0.4313 | 1.32 | 7.05E-09 |  | *A*02:01, A*02:06, A*02:07, A*02:10, A*02:11, A*03:01, A*23:01, A*29:01, A*29:02, A*30:01, A*30:02, A*31:01, A*32:01, A*32:03, A*33:03, A*69:01, A*74:02* |
| A | 156 | Q | - | 0.4836 | 0.3907 | 0.68 | 1.30E-15 |  | *A*03:02, A*11:01, A*11:02, A*11:04, A*24:02, A*24:03, A*24:04, A*24:05, A*24:07, A*24:10, A*24:20, A*24:21, A*24:64, A*24:68* |
| A | 156 | W | - | 0.1443 | 0.1740 | 1.25 | 4.35E-04 |  | *A*02:03, A*02:05, A*25:01, A*26:01, A*34:01, A*66:01, A*68:01, A*68:02* |
| A | 158 | V | A | 0.0066 | 0.0039 | 0.59 | 1.25E-01 |  | *A*01:01, A*01:03* |
| A | 161 | D | E | 0.0094 | 0.0039 | 0.41 | 6.19E-03 |  | *A*03:01, A*03:02* |
| A | 163 | R | T | 0.3560 | 0.2683 | 0.66 | 1.05E-15 |  | *A*01:01, A*01:03, A*11:01, A*11:02, A*24:10, A*25:01, A*26:01, A*66:01* |
| A | 166 | D | E | 0.1504 | 0.1466 | 0.97 | 6.51E-01 |  | *A*01:01, A*01:03, A*23:01, A*24:02, A*24:04, A*24:05, A*24:07, A*24:20, A*24:21, A*24:64, A*24:68* |
| A | 167 | G | W | 0.1504 | 0.1466 | 0.97 | 6.51E-01 |  | *A*01:01, A*01:03, A*23:01, A*24:02, A*24:04, A*24:05, A*24:07, A*24:20, A*24:21, A*24:64, A*24:68* |
| A | 184 | A | P | 0.3452 | 0.4173 | 1.36 | 1.56E-10 |  | *A*02:01, A*02:03, A*02:05, A*02:06, A*02:07, A*02:10, A*02:11, A*25:01, A*26:01, A*29:01, A*29:02, A*32:01, A*34:01, A*66:01, A*68:01, A*68:02, A*69:01* |
| A | 193 | P | A | 0.5072 | 0.4034 | 0.66 | 5.50E-19 |  | *A*01:01, A*03:01, A*03:02, A*11:01, A*11:02, A*11:04, A*23:01, A*24:02, A*24:03, A*24:04, A*24:07, A*24:10, A*24:20, A*30:01, A*30:02* |
| A | 194 | I | V | 0.5072 | 0.4034 | 0.66 | 5.50E-19 |  | *A*01:01, A*03:01, A*03:02, A*11:01, A*11:02, A*11:04, A*23:01, A*24:02, A*24:03, A*24:04, A*24:07, A*24:10, A*24:20, A*30:01, A*30:02* |
| A | 207 | G | S | 0.5072 | 0.4034 | 0.66 | 5.50E-19 |  | *A*01:01, A*03:01, A*03:02, A*11:01, A*11:02, A*11:04, A*23:01, A*24:02, A*24:03, A*24:04, A*24:07, A*24:10, A*24:20, A*30:01, A*30:02* |
| A | 245 | V | A | 0.0013 | 0.0000 | 0.00 | 5.40E-02 |  | *A*68:01, A*68:02* |
| A | 246 | S | A | 0.1754 | 0.1971 | 1.15 | 1.64E-02 |  | *A*25:01, A*26:01, A*29:01, A*29:02, A*31:01, A*32:01, A*33:03, A*34:01, A*66:01* |
| A | 253 | E | Q | 0.5072 | 0.4034 | 0.66 | 5.50E-19 |  | *A*01:01, A*03:01, A*03:02, A*11:01, A*11:02, A*11:04, A*23:01, A*24:02, A*24:03, A*24:04, A*24:07, A*24:10, A*24:20, A*30:01, A*30:02* |
| A | 276 | L | P | 0.3195 | 0.2150 | 0.58 | 3.29E-23 |  | *A*01:01, A*03:01, A*03:02, A*11:01, A*11:04, A*30:01, A*30:02* |
| A | 282 | V | I | 0.1469 | 0.1437 | 0.97 | 7.00E-01 |  | *A*23:01, A*24:02, A*24:03, A*24:04, A*24:07* |
| A | 283 | H | P | 0.0008 | 0.0011 | 1.42 | 6.47E-01 |  | *A*23:01* |
| A | 288 | L | I | 0.0011 | 0.0004 | 0.31 | 2.58E-01 |  | *A*34:01* |
| A | 294 | L | F | 0.5072 | 0.4034 | 0.66 | 5.31E-19 |  | *A*01:01, A*03:01, A*03:02, A*11:01, A*11:04, A*23:01, A*24:02, A*24:03, A*24:04, A*24:07, A*30:01, A*30:02* |
| A | 297 | M | V | 0.0077 | 0.0011 | 0.14 | 1.02E-04 |  | *A*32:01* |
| A | 298 | F | I | 0.1624 | 0.1808 | 1.14 | 3.52E-02 |  | *A*29:01, A*29:02, A*31:01, A*32:01, A*33:03* |
| A | 299 | A | T | 0.1754 | 0.1969 | 1.15 | 1.75E-02 |  | *A*25:01, A*26:01, A*29:01, A*29:02, A*31:01, A*32:01, A*33:03, A*34:01, A*66:01* |
| A | 307 | R | M | 0.1624 | 0.1808 | 1.14 | 3.52E-02 |  | *A*29:01, A*29:02, A*31:01, A*32:01, A*33:03* |
| A | 311 | N | K | 0.1469 | 0.1437 | 0.97 | 7.00E-01 |  | *A*23:01, A*24:02, A*24:03, A*24:04, A*24:07* |
| A | 321 | T | S | 0.3603 | 0.2596 | 0.62 | 3.67E-20 |  | *A*01:01, A*03:01, A*03:02, A*11:01, A*11:04, A*30:01, A*30:02* |
| A | 334 | M | V | 0.1754 | 0.1969 | 1.15 | 1.75E-02 |  | *A*25:01, A*26:01, A*29:01, A*29:02, A*31:01, A*32:01, A*33:03, A*34:01, A*66:01* |
| B | -23 | L | R | 0.1285 | 0.1573 | 1.27 | 3.72E-04 |  | *B*07:02, B*07:05, B*08:01, B*38:01, B*38:02, B*39:01, B*39:05, B*39:09, B*48:01, B*67:01* |
| B | -21 | M | T | 0.1285 | 0.1573 | 1.27 | 3.72E-04 |  | *B*07:02, B*07:05, B*08:01, B*38:01, B*38:02, B*39:01, B*39:05, B*39:09, B*48:01, B*67:01* |
| B | -16 | L | V | 0.2439 | 0.1726 | 0.65 | 1.70E-13 |  | *B*13:01, B*13:02, B*18:01, B*18:02, B*27:03, B*27:04, B*27:05, B*27:06, B*37:01, B*40:02, B*40:03, B*40:06, B*44:02, B*44:03, B*54:01, B*55:01, B*55:02, B*56:01, B*59:01* |
| B | -11 | W | S | 0.4594 | 0.4064 | 0.81 | 4.85E-06 |  | *B*13:01, B*13:02, B*18:01, B*18:02, B*27:03, B*27:04, B*27:05, B*27:06, B*35:01, B*35:02, B*35:03, B*35:05, B*35:08, B*37:01, B*40:02, B*40:03, B*40:06, B*44:02, B*44:03, B*51:01, B*51:02, B*52:01, B*54:01, B*55:01, B*55:02, B*56:01, B*57:01, B*58:01, B*59:01* |
| B | -10 | A | G | 0.2561 | 0.2924 | 1.20 | 4.64E-04 |  | *B*07:02, B*07:05, B*08:01, B*38:01, B*38:02, B*39:01, B*39:05, B*39:09, B*40:01, B*45:01, B*48:01, B*49:01, B*50:01, B*67:01* |
| B | -8 | V | L | 0.3793 | 0.3549 | 0.90 | 3.10E-02 |  | *B*13:01, B*13:02, B*18:01, B*18:02, B*27:03, B*27:04, B*27:05, B*27:06, B*35:01, B*35:02, B*35:03, B*35:05, B*35:08, B*37:01, B*40:02, B*40:03, B*40:06, B*44:02, B*44:03, B*51:01, B*51:02, B*52:01, B*57:01, B*58:01* |
| B | 9 | H | - | 0.1634 | 0.1658 | 1.02 | 7.78E-01 | 3.35E-02 | *B*18:01, B*18:02, B*27:03, B*27:04, B*27:05, B*27:06, B*27:07, B*37:01, B*40:01, B*40:02, B*40:03, B*40:06, B*45:01, B*49:01, B*50:01* |
| B | 9 | D | - | 0.0032 | 0.0004 | 0.11 | 9.41E-03 |  | *B*08:01* |
| B | 9 | Y | - | 0.1666 | 0.1662 | 1.00 | 9.62E-01 |  | *B*07:02, B*07:05, B*13:01, B*13:02, B*15:01, B*15:02, B*15:03, B*15:07, B*15:08, B*15:10, B*15:11, B*15:12, B*15:13, B*15:18, B*15:21, B*15:25, B*15:27, B*15:32, B*35:01, B*35:02, B*35:03, B*35:05, B*35:08, B*35:30, B*38:01, B*38:02, B*39:01, B*39:05, B*39:09, B*39:15, B*44:02, B*44:03, B*46:01, B*48:01, B*48:03, B*51:01, B*51:02, B*51:06, B*52:01, B*54:01, B*55:01, B*55:02, B*55:03, B*55:04, B*55:07, B*56:01, B*56:04, B*56:10, B*57:01, B*58:01, B*59:01, B*67:01* |
| B | 11 | S | A | 0.1619 | 0.1875 | 1.20 | 3.48E-03 |  | *B*07:02, B*07:05, B*18:01, B*18:02, B*27:03, B*27:04, B*27:05, B*27:06, B*27:07, B*37:01, B*38:01, B*38:02, B*39:01, B*39:05, B*39:09, B*39:15, B*40:02, B*40:03, B*40:06, B*48:01, B*48:03, B*67:01* |
| B | 12 | V | M | 0.1619 | 0.1875 | 1.20 | 3.48E-03 |  | *B*07:02, B*07:05, B*18:01, B*18:02, B*27:03, B*27:04, B*27:05, B*27:06, B*27:07, B*37:01, B*38:01, B*38:02, B*39:01, B*39:05, B*39:09, B*39:15, B*40:02, B*40:03, B*40:06, B*48:01, B*48:03, B*67:01* |
| B | 24 | T | - | 0.2836 | 0.2491 | 0.84 | 8.96E-04 | 1.13E-04 | *B*13:01, B*13:02, B*27:03, B*27:04, B*27:05, B*27:06, B*27:07, B*40:01, B*40:02, B*40:03, B*40:06, B*44:02, B*44:03, B*45:01, B*49:01, B*50:01* |
| B | 24 | A | - | 0.4208 | 0.4146 | 0.98 | 5.92E-01 |  | *B*15:01, B*15:02, B*15:07, B*15:08, B*15:11, B*15:12, B*15:13, B*15:21, B*15:25, B*15:27, B*15:32, B*35:01, B*35:02, B*35:03, B*35:05, B*35:08, B*35:30, B*46:01, B*51:01, B*51:02, B*51:06, B*52:01, B*54:01, B*55:01, B*55:02, B*55:03, B*55:04, B*55:07, B*56:01, B*56:04, B*56:10, B*57:01, B*58:01, B*59:01* |
| B | 24 | S | - | 0.1372 | 0.1655 | 1.25 | 6.12E-04 |  | *B*07:02, B*07:05, B*08:01, B*15:03, B*15:10, B*15:18, B*18:01, B*18:02, B*37:01, B*38:01, B*38:02, B*39:01, B*39:05, B*39:09, B*39:15, B*48:01, B*48:03, B*67:01* |
| B | 30 | G | D | 0.0028 | 0.0036 | 1.26 | 5.73E-01 |  | *B*18:01, B*18:02* |
| B | 32 | L | Q | 0.1608 | 0.1626 | 1.01 | 8.27E-01 |  | *B*27:03, B*27:04, B*27:05, B*27:06, B*27:07, B*40:01, B*40:02, B*40:03, B*40:06, B*44:02, B*44:03, B*45:01, B*49:01, B*50:01* |
| B | 41 | T | A | 0.2674 | 0.2434 | 0.88 | 1.92E-02 |  | *B*13:01, B*13:02, B*40:01, B*40:02, B*40:03, B*40:06, B*44:02, B*44:03, B*45:01, B*49:01, B*50:01* |
| B | 45 | K | - | 0.1445 | 0.1569 | 1.10 | 1.35E-01 | 2.00E-02 | *B*40:01, B*40:02, B*40:03, B*40:06, B*44:02, B*44:03, B*45:01, B*49:01, B*50:01* |
| B | 45 | M | - | 0.4068 | 0.3851 | 0.91 | 5.72E-02 |  | *B*13:01, B*13:02, B*15:01, B*15:02, B*15:07, B*15:08, B*15:11, B*15:12, B*15:13, B*15:21, B*15:25, B*15:27, B*15:32, B*46:01, B*57:01* |
| B | 45 | T | - | 0.2198 | 0.2406 | 1.12 | 3.37E-02 |  | *B*18:01, B*18:02, B*35:01, B*35:02, B*35:03, B*35:05, B*35:08, B*35:30, B*37:01, B*51:01, B*51:02, B*51:06, B*52:01, B*58:01* |
| B | 45 | G | - | 0.0226 | 0.0274 | 1.22 | 1.85E-01 |  | *B*54:01, B*55:07* |
| B | 45 | E | - | 0.2062 | 0.1900 | 0.90 | 8.32E-02 |  | *B*07:02, B*07:05, B*08:01, B*15:03, B*15:10, B*15:18, B*27:03, B*27:04, B*27:05, B*27:06, B*27:07, B*38:01, B*38:02, B*39:01, B*39:05, B*39:09, B*39:15, B*48:01, B*48:03, B*55:01, B*55:02, B*55:03, B*55:04, B*56:01, B*56:04, B*56:10, B*59:01, B*67:01* |
| B | 46 | A | E | 0.4068 | 0.3851 | 0.91 | 5.72E-02 |  | *B*13:01, B*13:02, B*15:01, B*15:02, B*15:07, B*15:08, B*15:11, B*15:12, B*15:13, B*15:21, B*15:25, B*15:27, B*15:32, B*46:01, B*57:01* |
| B | 52 | V | I | 0.0221 | 0.0274 | 1.25 | 1.35E-01 |  | *B*54:01* |
| B | 59 | H | Y | 0.0002 | 0.0000 | 0.00 | 4.67E-01 |  | *B*27:03* |
| B | 62 | G | R | 0.1400 | 0.1665 | 1.23 | 1.41E-03 |  | *B*57:01, B*58:01* |
| B | 63 | N | E | 0.3547 | 0.3445 | 0.96 | 3.58E-01 |  | *B*07:02, B*07:05, B*08:01, B*15:02, B*15:08, B*15:10, B*15:11, B*15:13, B*15:18, B*15:21, B*18:01, B*18:02, B*35:01, B*35:02, B*35:03, B*35:05, B*35:08, B*35:30, B*38:01, B*38:02, B*39:01, B*39:05, B*39:09, B*39:15, B*51:01, B*51:02, B*51:06, B*54:01, B*55:01, B*55:02, B*55:03, B*55:04, B*55:07, B*56:01, B*56:04, B*56:10, B*59:01, B*67:01* |
| B | 65 | R | Q | 0.1400 | 0.1665 | 1.23 | 1.41E-03 |  | *B*57:01, B*58:01* |
| B | 66 | N | - | 0.1400 | 0.1665 | 1.23 | 1.41E-03 | 1.34E-06 | *B*57:01, B*58:01* |
| B | 66 | K | - | 0.1617 | 0.1918 | 1.23 | 6.29E-04 |  | *B*46:01* |
| B | 66 | I | - | 0.3017 | 0.3584 | 1.29 | 2.00E-07 |  | *B*07:02, B*07:05, B*08:01, B*13:01, B*13:02, B*15:01, B*15:02, B*15:03, B*15:07, B*15:08, B*15:10, B*15:11, B*15:12, B*15:13, B*15:18, B*15:21, B*15:25, B*15:27, B*15:32, B*18:01, B*18:02, B*27:03, B*27:04, B*27:05, B*27:06, B*27:07, B*35:01, B*35:02, B*35:03, B*35:05, B*35:08, B*35:30, B*37:01, B*38:01, B*38:02, B*39:01, B*39:05, B*39:09, B*39:15, B*40:01, B*40:02, B*40:03, B*40:06, B*44:02, B*44:03, B*45:01, B*48:01, B*48:03, B*49:01, B*50:01, B*51:01, B*51:02, B*51:06, B*52:01, B*54:01, B*55:01, B*55:02, B*55:03, B*55:04, B*55:07, B*56:01, B*56:04, B*56:10, B*59:01, B*67:01* |
| B | 67 | Y | - | 0.2558 | 0.2527 | 0.98 | 7.55E-01 | 1.46E-05 | *B*07:02, B*07:05, B*15:11, B*46:01, B*54:01, B*55:01, B*55:02, B*55:03, B*55:04, B*55:07, B*56:01, B*56:04, B*56:10, B*67:01* |
| B | 67 | F | - | 0.0728 | 0.0655 | 0.89 | 2.18E-01 |  | *B*08:01, B*15:08, B*35:01, B*35:02, B*35:03, B*35:05, B*35:08, B*35:30, B*51:01, B*51:02, B*51:06, B*59:01* |
| B | 67 | C | - | 0.1117 | 0.1370 | 1.26 | 8.61E-04 |  | *B*15:10, B*15:18, B*15:21, B*27:03, B*27:04, B*27:05, B*27:06, B*27:07, B*38:01, B*38:02, B*39:01, B*39:05, B*39:09, B*39:15* |
| B | 67 | S | - | 0.4196 | 0.3783 | 0.84 | 3.09E-04 |  | *B*13:01, B*13:02, B*15:01, B*15:02, B*15:03, B*15:07, B*15:12, B*15:13, B*15:25, B*15:27, B*15:32, B*18:01, B*18:02, B*37:01, B*40:01, B*40:02, B*40:03, B*40:06, B*44:02, B*44:03, B*45:01, B*48:01, B*48:03, B*49:01, B*50:01, B*52:01* |
| B | 67 | M | - | 0.1400 | 0.1665 | 1.23 | 1.41E-03 |  | *B*57:01, B*58:01* |
| B | 69 | R | - | 0.1617 | 0.1918 | 1.23 | 6.29E-04 | 1.90E-03 | *B*46:01* |
| B | 69 | T | - | 0.4104 | 0.4228 | 1.05 | 2.81E-01 |  | *B*08:01, B*13:01, B*13:02, B*15:01, B*15:02, B*15:03, B*15:07, B*15:08, B*15:10, B*15:11, B*15:12, B*15:13, B*15:18, B*15:21, B*15:25, B*15:27, B*15:32, B*18:01, B*18:02, B*35:01, B*35:02, B*35:03, B*35:05, B*35:08, B*35:30, B*37:01, B*38:01, B*38:02, B*39:01, B*39:05, B*39:09, B*39:15, B*40:01, B*40:02, B*40:03, B*40:06, B*44:02, B*44:03, B*45:01, B*48:01, B*48:03, B*49:01, B*50:01, B*51:01, B*51:02, B*51:06, B*52:01, B*59:01* |
| B | 69 | A | - | 0.2487 | 0.2310 | 0.91 | 7.65E-02 |  | *B*07:02, B*07:05, B*27:03, B*27:04, B*27:05, B*27:06, B*27:07, B*54:01, B*55:01, B*55:02, B*55:03, B*55:04, B*55:07, B*56:01, B*56:04, B*56:10, B*57:01, B*58:01, B*67:01* |
| B | 70 | S | - | 0.1400 | 0.1665 | 1.23 | 1.41E-03 | 1.24E-05 | *B*57:01, B*58:01* |
| B | 70 | N | - | 0.4104 | 0.4228 | 1.05 | 2.81E-01 |  | *B*08:01, B*13:01, B*13:02, B*15:01, B*15:02, B*15:03, B*15:07, B*15:08, B*15:10, B*15:11, B*15:12, B*15:13, B*15:18, B*15:21, B*15:25, B*15:27, B*15:32, B*18:01, B*18:02, B*35:01, B*35:02, B*35:03, B*35:05, B*35:08, B*35:30, B*37:01, B*38:01, B*38:02, B*39:01, B*39:05, B*39:09, B*39:15, B*40:01, B*40:02, B*40:03, B*40:06, B*44:02, B*44:03, B*45:01, B*48:01, B*48:03, B*49:01, B*50:01, B*51:01, B*51:02, B*51:06, B*52:01, B*59:01* |
| B | 70 | Q | - | 0.2542 | 0.2505 | 0.98 | 7.21E-01 |  | *B*07:02, B*07:05, B*46:01, B*54:01, B*55:01, B*55:02, B*55:03, B*55:04, B*55:07, B*56:01, B*56:04, B*56:10, B*67:01* |
| B | 70 | K | - | 0.0162 | 0.0057 | 0.35 | 5.12E-05 |  | *B*27:03, B*27:04, B*27:05, B*27:06, B*27:07* |
| B | 71 | A | T | 0.4104 | 0.4228 | 1.05 | 2.81E-01 |  | *B*07:02, B*07:05, B*27:03, B*27:04, B*27:05, B*27:06, B*27:07, B*46:01, B*54:01, B*55:01, B*55:02, B*55:03, B*55:04, B*55:07, B*56:01, B*56:04, B*56:10, B*57:01, B*58:01, B*67:01* |
| B | 74 | D | Y | 0.2855 | 0.2623 | 0.89 | 2.64E-02 |  | *B*07:02, B*07:05, B*08:01, B*27:03, B*27:04, B*27:05, B*27:06, B*27:07, B*39:01, B*39:09, B*39:15, B*46:01, B*54:01, B*55:01, B*55:02, B*55:03, B*55:04, B*55:07, B*56:01, B*56:04, B*56:10, B*67:01* |
| B | 76 | V | E | 0.1619 | 0.1918 | 1.23 | 6.83E-04 |  | *B*46:01, B*55:03* |
| B | 77 | D | - | 0.0055 | 0.0032 | 0.58 | 1.55E-01 | 7.10E-02 | *B*27:03, B*27:05, B*27:07, B*37:01* |
| B | 77 | N | - | 0.4015 | 0.4231 | 1.09 | 5.95E-02 |  | *B*13:01, B*13:02, B*15:13, B*38:01, B*38:02, B*44:02, B*44:03, B*49:01, B*51:01, B*51:02, B*51:06, B*52:01, B*57:01, B*58:01, B*59:01* |
| B | 77 | S | - | 0.4070 | 0.4263 | 1.08 | 9.22E-02 |  | *B*07:02, B*07:05, B*08:01, B*15:01, B*15:02, B*15:03, B*15:07, B*15:08, B*15:10, B*15:11, B*15:12, B*15:18, B*15:21, B*15:25, B*15:27, B*15:32, B*18:01, B*18:02, B*27:04, B*27:06, B*35:01, B*35:02, B*35:03, B*35:05, B*35:08, B*35:30, B*39:01, B*39:05, B*39:09, B*39:15, B*40:01, B*40:02, B*40:03, B*40:06, B*45:01, B*46:01, B*48:01, B*48:03, B*50:01, B*54:01, B*55:01, B*55:02, B*55:03, B*55:04, B*55:07, B*56:01, B*56:04, B*56:10, B*67:01* |
| B | 80 | I | - | 0.1979 | 0.2114 | 1.09 | 1.51E-01 | 3.56E-01 | *B*15:13, B*38:01, B*49:01, B*51:01, B*51:02, B*51:06, B*52:01, B*57:01, B*58:01, B*59:01* |
| B | 80 | T | - | 0.2236 | 0.2203 | 0.98 | 7.34E-01 |  | *B*13:01, B*13:02, B*27:03, B*27:04, B*27:05, B*27:06, B*27:07, B*37:01, B*38:02, B*44:02, B*44:03* |
| B | 80 | N | - | 0.4215 | 0.4317 | 1.04 | 3.78E-01 |  | *B*07:02, B*07:05, B*08:01, B*15:01, B*15:02, B*15:03, B*15:07, B*15:08, B*15:10, B*15:11, B*15:12, B*15:18, B*15:21, B*15:25, B*15:27, B*15:32, B*18:01, B*18:02, B*35:01, B*35:02, B*35:03, B*35:05, B*35:08, B*35:30, B*39:01, B*39:05, B*39:09, B*39:15, B*40:01, B*40:02, B*40:03, B*40:06, B*45:01, B*46:01, B*48:01, B*48:03, B*50:01, B*54:01, B*55:01, B*55:02, B*55:03, B*55:04, B*55:07, B*56:01, B*56:04, B*56:10, B*67:01* |
| B | 81 | A | L | 0.4015 | 0.4231 | 1.09 | 5.95E-02 |  | *B*13:01, B*13:02, B*15:13, B*38:01, B*38:02, B*44:02, B*44:03, B*49:01, B*51:01, B*51:02, B*51:06, B*52:01, B*57:01, B*58:01, B*59:01* |
| B | 82 | L | R | 0.4215 | 0.4317 | 1.04 | 3.78E-01 |  | *B*13:01, B*13:02, B*15:13, B*27:03, B*27:04, B*27:05, B*27:06, B*27:07, B*37:01, B*38:01, B*38:02, B*44:02, B*44:03, B*49:01, B*51:01, B*51:02, B*51:06, B*52:01, B*57:01, B*58:01, B*59:01* |
| B | 83 | R | G | 0.4215 | 0.4317 | 1.04 | 3.78E-01 |  | *B*13:01, B*13:02, B*15:13, B*27:03, B*27:04, B*27:05, B*27:06, B*27:07, B*37:01, B*38:01, B*38:02, B*44:02, B*44:03, B*49:01, B*51:01, B*51:02, B*51:06, B*52:01, B*57:01, B*58:01, B*59:01* |
| B | 94 | I | T | 0.3691 | 0.3541 | 0.94 | 1.83E-01 |  | *B*13:01, B*15:02, B*15:13, B*15:21, B*15:25, B*35:01, B*35:02, B*35:03, B*35:08, B*35:30, B*44:02, B*44:03, B*57:01, B*58:01* |
| B | 95 | W | - | 0.1421 | 0.1025 | 0.69 | 3.95E-07 | 2.66E-08 | *B*13:02, B*40:06, B*45:01, B*49:01, B*50:01, B*51:01, B*51:02, B*52:01, B*54:01, B*55:01, B*55:02, B*55:03, B*55:07, B*56:01, B*59:01* |
| B | 95 | I | - | 0.3728 | 0.3569 | 0.93 | 1.58E-01 |  | *B*13:01, B*15:02, B*15:13, B*15:21, B*15:25, B*35:01, B*35:02, B*35:03, B*35:08, B*35:30, B*37:01, B*44:02, B*44:03, B*57:01, B*58:01* |
| B | 95 | L | - | 0.5149 | 0.4594 | 0.80 | 1.99E-06 |  | *B*07:02, B*07:05, B*08:01, B*15:01, B*15:03, B*15:07, B*15:08, B*15:10, B*15:11, B*15:12, B*15:18, B*15:27, B*15:32, B*18:01, B*18:02, B*27:03, B*27:04, B*27:05, B*27:06, B*27:07, B*35:05, B*38:01, B*38:02, B*39:01, B*39:05, B*39:09, B*39:15, B*40:01, B*40:02, B*40:03, B*46:01, B*48:01, B*48:03, B*51:06, B*55:04, B*56:04, B*56:10, B*67:01* |
| B | 97 | V | - | 0.0019 | 0.0000 | 0.00 | 2.12E-02 | 6.86E-11 | *B*57:01* |
| B | 97 | N | - | 0.0176 | 0.0068 | 0.38 | 7.48E-05 |  | *B*18:02, B*27:03, B*27:04, B*27:05, B*27:06* |
| B | 97 | T | - | 0.1400 | 0.1011 | 0.69 | 5.20E-07 |  | *B*13:02, B*40:06, B*51:01, B*51:02, B*52:01, B*54:01, B*55:01, B*55:02, B*55:03, B*55:07, B*56:01, B*59:01* |
| B | 97 | R | - | 0.2036 | 0.1441 | 0.66 | 4.41E-11 |  | *B*13:01, B*15:01, B*15:02, B*15:03, B*15:08, B*15:10, B*15:11, B*15:12, B*15:13, B*15:18, B*15:21, B*15:25, B*15:27, B*15:32, B*18:01, B*35:01, B*35:02, B*35:03, B*35:08, B*37:01, B*38:01, B*38:02, B*39:01, B*39:05, B*39:09, B*39:15, B*40:01, B*44:02, B*44:03, B*45:01, B*46:01, B*48:03, B*49:01, B*50:01, B*51:06, B*56:04, B*56:10, B*58:01, B*67:01* |
| B | 97 | S | - | 0.0442 | 0.0363 | 0.82 | 9.13E-02 |  | *B*07:02, B*07:05, B*08:01, B*15:07, B*27:07, B*35:05, B*35:30, B*40:02, B*40:03, B*48:01, B*55:04* |
| B | 99 | S | - | 0.0047 | 0.0039 | 0.83 | 6.05E-01 | 8.41E-01 | *B*15:32, B*37:01, B*39:09* |
| B | 99 | F | - | 0.0036 | 0.0032 | 0.89 | 7.80E-01 |  | *B*15:27* |
| B | 99 | Y | - | 0.0083 | 0.0071 | 0.86 | 5.66E-01 |  | *B*07:02, B*07:05, B*08:01, B*13:01, B*13:02, B*15:01, B*15:02, B*15:03, B*15:07, B*15:08, B*15:10, B*15:11, B*15:12, B*15:13, B*15:18, B*15:21, B*15:25, B*18:01, B*18:02, B*27:03, B*27:04, B*27:05, B*27:06, B*27:07, B*35:01, B*35:02, B*35:03, B*35:05, B*35:08, B*35:30, B*38:01, B*38:02, B*39:01, B*39:05, B*39:15, B*40:01, B*40:02, B*40:03, B*40:06, B*44:02, B*44:03, B*45:01, B*46:01, B*48:01, B*48:03, B*49:01, B*50:01, B*51:01, B*51:02, B*51:06, B*52:01, B*54:01, B*55:01, B*55:02, B*55:03, B*55:04, B*55:07, B*56:01, B*56:04, B*56:10, B*57:01, B*58:01, B*59:01, B*67:01* |
| B | 103 | L | V | 0.3609 | 0.3274 | 0.86 | 2.57E-03 |  | *B*13:01, B*13:02, B*35:01, B*35:02, B*35:03, B*35:05, B*35:08, B*35:30, B*45:01, B*49:01, B*50:01, B*54:01, B*55:01, B*55:02, B*55:03, B*55:07, B*56:01, B*56:10, B*58:01, B*59:01* |
| B | 109 | F | L | 0.0006 | 0.0000 | 0.00 | 2.07E-01 |  | *B*35:02* |
| B | 113 | Y | H | 0.1230 | 0.1007 | 0.80 | 2.76E-03 |  | *B*15:02, B*15:13, B*15:21, B*15:25, B*27:03, B*27:04, B*27:05, B*27:06, B*37:01, B*44:02, B*44:03, B*45:01, B*49:01, B*50:01* |
| B | 114 | H | - | 0.0151 | 0.0053 | 0.35 | 1.02E-04 | 1.24E-06 | *B*27:03, B*27:04, B*27:05* |
| B | 114 | N | - | 0.4694 | 0.5046 | 1.15 | 2.54E-03 |  | *B*07:05, B*08:01, B*13:01, B*13:02, B*27:07, B*35:02, B*37:01, B*38:01, B*38:02, B*39:01, B*39:05, B*39:09, B*40:01, B*40:02, B*40:06, B*45:01, B*48:01, B*48:03, B*49:01, B*50:01, B*51:01, B*51:02, B*51:06, B*52:01, B*54:01, B*55:01, B*55:02, B*55:03, B*55:04, B*55:07, B*56:01, B*56:04, B*56:10, B*59:01, B*67:01* |
| B | 114 | D | - | 0.4543 | 0.4993 | 1.20 | 1.14E-04 |  | *B*07:02, B*15:01, B*15:02, B*15:03, B*15:07, B*15:08, B*15:10, B*15:11, B*15:12, B*15:13, B*15:18, B*15:21, B*15:25, B*15:27, B*15:32, B*18:01, B*18:02, B*27:06, B*35:01, B*35:03, B*35:05, B*35:08, B*35:30, B*39:15, B*40:03, B*44:02, B*44:03, B*46:01, B*57:01, B*58:01* |
| B | 116 | F | - | 0.1025 | 0.1416 | 1.45 | 1.58E-07 | 6.51E-19 | *B*35:03, B*37:01, B*38:01, B*38:02, B*39:01, B*39:05, B*39:09, B*39:15, B*67:01* |
| B | 116 | L | - | 0.2068 | 0.1406 | 0.63 | 2.37E-13 |  | *B*13:01, B*13:02, B*45:01, B*49:01, B*50:01, B*54:01, B*55:01, B*55:02, B*55:03, B*55:07, B*56:01, B*56:04, B*56:10, B*59:01* |
| B | 116 | D | - | 0.0191 | 0.0085 | 0.44 | 2.54E-04 |  | *B*27:03, B*27:04, B*27:05, B*44:02, B*44:03* |
| B | 116 | S | - | 0.4426 | 0.4907 | 1.21 | 3.52E-05 |  | *B*15:01, B*15:02, B*15:03, B*15:07, B*15:08, B*15:11, B*15:12, B*15:13, B*15:18, B*15:21, B*15:25, B*15:27, B*15:32, B*18:01, B*18:02, B*35:01, B*35:05, B*35:08, B*35:30, B*40:03, B*46:01, B*57:01, B*58:01* |
| B | 116 | Y | - | 0.2291 | 0.2185 | 0.94 | 2.79E-01 |  | *B*07:02, B*07:05, B*08:01, B*15:10, B*27:06, B*27:07, B*35:02, B*40:01, B*40:02, B*40:06, B*48:01, B*48:03, B*51:01, B*51:02, B*51:06, B*52:01, B*55:04* |
| B | 131 | R | S | 0.1728 | 0.1758 | 1.02 | 7.37E-01 |  | *B*07:02, B*07:05, B*08:01, B*27:07, B*40:01, B*40:02, B*40:03, B*40:06, B*48:01, B*48:03, B*55:04* |
| B | 143 | S | T | 0.1472 | 0.1548 | 1.06 | 3.59E-01 |  | *B*40:01, B*48:01, B*48:03* |
| B | 145 | L | R | 0.1228 | 0.0865 | 0.68 | 6.55E-07 |  | *B*13:01, B*13:02* |
| B | 147 | L | W | 0.1472 | 0.1548 | 1.06 | 3.59E-01 |  | *B*40:01, B*48:01, B*48:03* |
| B | 152 | E | V | 0.3640 | 0.3523 | 0.95 | 2.98E-01 |  | *B*07:02, B*07:05, B*15:01, B*15:02, B*15:03, B*15:07, B*15:08, B*15:10, B*15:11, B*15:12, B*15:13, B*15:18, B*15:21, B*15:25, B*15:27, B*15:32, B*27:04, B*27:06, B*46:01, B*49:01, B*50:01, B*51:01, B*51:02, B*51:06, B*52:01, B*55:01, B*55:03* |
| B | 156 | W | - | 0.1851 | 0.2107 | 1.18 | 5.56E-03 | 4.95E-06 | *B*15:01, B*15:07, B*15:08, B*15:11, B*15:12, B*15:27, B*15:32, B*46:01* |
| B | 156 | L | - | 0.2019 | 0.2160 | 1.09 | 1.35E-01 |  | *B*13:01, B*13:02, B*15:02, B*15:03, B*15:10, B*15:13, B*15:18, B*15:21, B*15:25, B*18:01, B*18:02, B*27:03, B*27:04, B*27:05, B*27:06, B*27:07, B*35:01, B*35:02, B*35:03, B*35:05, B*35:30, B*38:01, B*38:02, B*39:01, B*39:05, B*39:09, B*39:15, B*40:01, B*40:02, B*40:03, B*40:06, B*44:03, B*48:01, B*48:03, B*49:01, B*50:01, B*51:01, B*51:02, B*51:06, B*52:01, B*54:01, B*55:01, B*55:02, B*55:03, B*55:04, B*55:07, B*56:01, B*56:04, B*56:10, B*57:01, B*58:01, B*59:01, B*67:01* |
| B | 156 | D | - | 0.0079 | 0.0039 | 0.49 | 3.30E-02 |  | *B*08:01, B*37:01, B*44:02, B*45:01* |
| B | 156 | R | - | 0.0089 | 0.0014 | 0.16 | 5.45E-05 |  | *B*07:02, B*07:05, B*35:08* |
| B | 158 | T | A | 0.0945 | 0.1338 | 1.48 | 5.96E-08 |  | *B*38:01, B*38:02, B*39:01, B*39:05, B*39:09, B*39:15, B*67:01* |
| B | 163 | L | - | 0.4732 | 0.4473 | 0.90 | 2.62E-02 | 4.41E-04 | *B*15:01, B*15:02, B*15:03, B*15:07, B*15:08, B*15:10, B*15:11, B*15:12, B*15:13, B*15:18, B*15:21, B*15:25, B*15:27, B*15:32, B*35:01, B*35:02, B*35:03, B*35:05, B*35:08, B*35:30, B*44:02, B*44:03, B*45:01, B*46:01, B*49:01, B*50:01, B*51:01, B*51:02, B*51:06, B*52:01, B*56:01, B*56:04, B*57:01, B*58:01* |
| B | 163 | T | - | 0.1647 | 0.1801 | 1.11 | 7.99E-02 |  | *B*08:01, B*18:01, B*18:02, B*37:01, B*38:01, B*38:02, B*39:01, B*39:05, B*39:09, B*39:15, B*54:01, B*55:01, B*55:02, B*55:03, B*55:04, B*55:07, B*56:10, B*59:01, B*67:01* |
| B | 163 | E | - | 0.3085 | 0.2673 | 0.82 | 1.06E-04 |  | *B*07:02, B*07:05, B*13:01, B*13:02, B*27:03, B*27:04, B*27:05, B*27:06, B*27:07, B*40:01, B*40:02, B*40:03, B*40:06, B*48:01, B*48:03* |
| B | 166 | D | E | 0.0060 | 0.0039 | 0.65 | 2.10E-01 |  | *B*15:12* |
| B | 167 | G | - | 0.0060 | 0.0039 | 0.65 | 2.10E-01 | 3.70E-01 | *B*15:12* |
| B | 167 | S | - | 0.0045 | 0.0036 | 0.79 | 5.20E-01 |  | *B*44:02, B*44:03, B*45:01* |
| B | 167 | W | - | 0.0106 | 0.0075 | 0.71 | 1.72E-01 |  | *B*07:02, B*07:05, B*08:01, B*13:01, B*13:02, B*15:01, B*15:02, B*15:03, B*15:07, B*15:08, B*15:10, B*15:11, B*15:13, B*15:18, B*15:21, B*15:25, B*15:27, B*15:32, B*18:01, B*18:02, B*27:03, B*27:04, B*27:05, B*27:06, B*27:07, B*35:01, B*35:02, B*35:03, B*35:05, B*35:08, B*35:30, B*37:01, B*38:01, B*38:02, B*39:01, B*39:05, B*39:09, B*39:15, B*40:01, B*40:02, B*40:03, B*40:06, B*46:01, B*48:01, B*48:03, B*49:01, B*50:01, B*51:01, B*51:02, B*51:06, B*52:01, B*54:01, B*55:01, B*55:02, B*55:03, B*55:04, B*55:07, B*56:01, B*56:04, B*56:10, B*57:01, B*58:01, B*59:01, B*67:01* |
| B | 171 | H | Y | 0.0517 | 0.0445 | 0.85 | 1.53E-01 |  | *B*18:01, B*18:02, B*51:01, B*51:06, B*52:01* |
| B | 177 | D | E | 0.1591 | 0.1566 | 0.98 | 7.72E-01 |  | *B*07:02, B*07:05, B*08:01, B*40:01, B*48:01, B*48:03* |
| B | 178 | K | T | 0.1558 | 0.1562 | 1.00 | 9.64E-01 |  | *B*07:02, B*07:05, B*40:01, B*48:01, B*48:03* |
| B | 180 | E | Q | 0.1591 | 0.1566 | 0.98 | 7.72E-01 |  | *B*07:02, B*07:05, B*08:01, B*40:01, B*48:01, B*48:03* |
| B | 194 | V | I | 0.2134 | 0.2340 | 1.13 | 3.38E-02 |  | *B*35:01, B*35:02, B*35:03, B*35:05, B*35:08, B*51:01, B*51:02, B*51:06, B*52:01, B*58:01* |
| B | 199 | V | A | 0.0040 | 0.0032 | 0.81 | 5.90E-01 |  | *B*44:02, B*44:03* |
| B | 211 | G | A | 0.0146 | 0.0053 | 0.36 | 1.96E-04 |  | *B*27:04, B*27:06* |
| B | 245 | T | A | 0.0223 | 0.0214 | 0.96 | 7.84E-01 |  | *B*48:01, B*48:03* |
| B | 282 | V | I | 0.4058 | 0.4046 | 0.99 | 9.15E-01 |  | *B*07:02, B*08:01, B*13:01, B*13:02, B*27:03, B*27:04, B*27:05, B*27:06, B*27:07, B*38:01, B*38:02, B*39:01, B*39:05, B*39:09, B*40:01, B*40:02, B*40:03, B*40:06, B*44:02, B*44:03, B*48:01, B*57:01, B*67:01* |
| B | 305 | A | T | 0.4130 | 0.4056 | 0.97 | 5.23E-01 |  | *B*07:02, B*07:05, B*08:01, B*13:01, B*13:02, B*27:03, B*27:04, B*27:05, B*27:06, B*27:07, B*38:01, B*38:02, B*39:01, B*39:05, B*39:09, B*40:01, B*40:02, B*40:03, B*40:06, B*44:02, B*44:03, B*48:01, B*57:01, B*67:01* |
| B | 325 | C | S | 0.3184 | 0.2716 | 0.80 | 1.31E-05 |  | *B*07:02, B*07:05, B*08:01, B*13:01, B*13:02, B*27:03, B*27:04, B*27:05, B*27:06, B*27:07, B*40:01, B*40:02, B*40:03, B*40:06, B*44:02, B*44:03, B*48:01, B*57:01* |
| C | -17 | A | T | 0.1884 | 0.2244 | 1.25 | 1.18E-04 |  | *C*07:01, C*07:02, C*07:04* |
| C | -15 | L | I | 0.2170 | 0.2427 | 1.16 | 8.55E-03 |  | *C*02:02, C*07:01, C*07:02, C*07:04, C*15:02, C*15:04, C*15:05, C*15:17* |
| C | -9 | G | A | 0.1884 | 0.2244 | 1.25 | 1.18E-04 |  | *C*07:01, C*07:02, C*07:04* |
| C | 1 | G | C | 0.3754 | 0.3620 | 0.94 | 2.36E-01 |  | *C*03:02, C*03:03, C*03:04, C*04:01, C*04:03* |
| C | 6 | K | R | 0.1998 | 0.2217 | 1.14 | 2.07E-02 |  | *C*01:02, C*01:03, C*01:06* |
| C | 9 | S | - | 0.0677 | 0.0605 | 0.89 | 2.09E-01 | 7.56E-05 | *C*04:01, C*14:02, C*14:03* |
| C | 9 | D | - | 0.2032 | 0.2345 | 1.20 | 1.07E-03 |  | *C*06:02, C*07:01, C*07:02, C*07:04, C*07:43* |
| C | 9 | Y | - | 0.4708 | 0.5167 | 1.20 | 8.10E-05 |  | *C*02:02, C*03:02, C*03:03, C*03:04, C*03:17, C*03:36, C*04:03, C*05:01, C*08:01, C*12:02, C*12:03, C*15:02, C*15:04, C*15:05, C*15:17, C*16:02, C*16:04* |
| C | 9 | F | - | 0.1998 | 0.2217 | 1.14 | 2.07E-02 |  | *C*01:02, C*01:03, C*01:06* |
| C | 11 | S | A | 0.2675 | 0.2822 | 1.08 | 1.58E-01 |  | *C*01:02, C*01:03, C*01:06, C*04:01, C*14:02, C*14:03* |
| C | 14 | W | R | 0.0276 | 0.0274 | 0.99 | 9.70E-01 |  | *C*04:01* |
| C | 16 | S | G | 0.0249 | 0.0160 | 0.64 | 9.11E-03 |  | *C*02:02, C*04:03* |
| C | 21 | H | R | 0.3775 | 0.3537 | 0.90 | 3.45E-02 |  | *C*02:02, C*03:02, C*03:03, C*03:04, C*03:17, C*03:36, C*04:03, C*14:03, C*15:02, C*15:04, C*15:05, C*15:17* |
| C | 24 | S | A | 0.4030 | 0.4562 | 1.24 | 3.88E-06 |  | *C*01:02, C*01:03, C*01:06, C*06:02, C*07:01, C*07:02, C*07:04, C*07:43* |
| C | 35 | Q | R | 0.1096 | 0.1117 | 1.02 | 7.72E-01 |  | *C*05:01, C*08:01* |
| C | 49 | E | A | 0.0276 | 0.0274 | 0.99 | 9.70E-01 |  | *C*04:01* |
| C | 66 | N | K | 0.0300 | 0.0199 | 0.66 | 7.22E-03 |  | *C*07:01, C*15:02, C*15:04, C*15:05* |
| C | 73 | A | T | 0.2953 | 0.2943 | 1.00 | 9.27E-01 |  | *C*04:01, C*04:03, C*06:02, C*07:01, C*07:02, C*07:04, C*07:43, C*12:02, C*12:03* |
| C | 77 | N | S | 0.0943 | 0.0722 | 0.75 | 7.64E-04 |  | *C*02:02, C*04:01, C*04:03, C*05:01, C*06:02, C*15:02, C*15:04, C*15:05, C*15:17, C*16:02* |
| C | 80 | K | N | 0.0943 | 0.0722 | 0.75 | 7.64E-04 |  | *C*02:02, C*04:01, C*04:03, C*05:01, C*06:02, C*15:02, C*15:04, C*15:05, C*15:17, C*16:02* |
| C | 90 | D | A | 0.2536 | 0.2772 | 1.13 | 2.12E-02 |  | *C*04:01, C*04:03, C*06:02, C*07:01, C*07:02, C*07:04, C*07:43* |
| C | 91 | R | G | 0.0255 | 0.0196 | 0.76 | 9.47E-02 |  | *C*03:03* |
| C | 94 | I | T | 0.3511 | 0.3363 | 0.94 | 1.82E-01 |  | *C*03:02, C*03:03, C*03:04, C*03:36, C*07:43, C*15:02, C*15:04, C*15:05, C*15:17* |
| C | 95 | F | - | 0.0028 | 0.0050 | 1.76 | 1.22E-01 | 9.98E-06 | *C*07:04* |
| C | 95 | I | - | 0.2155 | 0.1726 | 0.76 | 4.48E-06 |  | *C*03:03, C*03:04, C*07:43, C*15:02, C*15:04, C*15:05, C*15:17* |
| C | 95 | L | - | 0.2183 | 0.1776 | 0.77 | 1.50E-05 |  | *C*01:02, C*01:03, C*01:06, C*02:02, C*03:02, C*03:17, C*03:36, C*04:01, C*04:03, C*05:01, C*06:02, C*07:01, C*07:02, C*08:01, C*12:02, C*12:03, C*14:02, C*14:03, C*16:02, C*16:04* |
| C | 97 | W | R | 0.2760 | 0.2754 | 1.00 | 9.55E-01 |  | *C*01:02, C*01:03, C*01:06, C*03:17, C*06:02, C*12:03, C*14:02, C*14:03, C*16:02, C*16:04* |
| C | 99 | F | - | 0.0906 | 0.0758 | 0.82 | 2.36E-02 | 7.22E-06 | *C*04:01, C*04:03, C*14:02, C*14:03* |
| C | 99 | Y | - | 0.4721 | 0.5139 | 1.18 | 3.38E-04 |  | *C*02:02, C*03:02, C*03:03, C*03:04, C*03:17, C*03:36, C*05:01, C*06:02, C*07:01, C*07:04, C*07:43, C*08:01, C*12:02, C*12:03, C*15:02, C*15:04, C*15:05, C*15:17, C*16:02, C*16:04* |
| C | 99 | S | - | 0.1817 | 0.2164 | 1.24 | 1.70E-04 |  | *C*07:02* |
| C | 99 | C | - | 0.1998 | 0.2217 | 1.14 | 2.07E-02 |  | *C*01:02, C*01:03, C*01:06* |
| C | 103 | V | L | 0.3255 | 0.3203 | 0.98 | 6.35E-01 |  | *C*03:02, C*03:03, C*03:04, C*03:17, C*03:36* |
| C | 113 | H | Y | 0.0264 | 0.0167 | 0.63 | 5.67E-03 |  | *C*15:02, C*15:05, C*15:17* |
| C | 114 | N | D | 0.1634 | 0.1559 | 0.95 | 3.80E-01 |  | *C*01:03, C*04:01, C*04:03, C*05:01, C*08:01* |
| C | 116 | L | - | 0.0200 | 0.0160 | 0.80 | 2.06E-01 | 1.18E-01 | *C*15:02, C*15:17* |
| C | 116 | S | - | 0.4211 | 0.4456 | 1.11 | 3.45E-02 |  | *C*02:02, C*03:02, C*03:36, C*06:02, C*07:01, C*07:02, C*07:43, C*12:02, C*12:03, C*14:02, C*14:03, C*15:04, C*16:02, C*16:04* |
| C | 116 | F | - | 0.1726 | 0.1616 | 0.92 | 2.05E-01 |  | *C*01:03, C*04:01, C*04:03, C*05:01, C*07:04, C*08:01, C*15:05* |
| C | 116 | Y | - | 0.3862 | 0.3769 | 0.96 | 4.09E-01 |  | *C*01:02, C*01:06, C*03:03, C*03:04, C*03:17* |
| C | 138 | K | T | 0.0002 | 0.0004 | 1.89 | 6.48E-01 |  | *C*05:01* |
| C | 147 | L | W | 0.1889 | 0.2242 | 1.24 | 1.59E-04 |  | *C*07:01, C*07:02, C*07:04, C*07:43* |
| C | 152 | T | - | 0.1094 | 0.1114 | 1.02 | 7.89E-01 | 1.27E-03 | *C*08:01* |
| C | 152 | A | - | 0.1900 | 0.2249 | 1.24 | 1.95E-04 |  | *C*07:01, C*07:02, C*07:04, C*07:43, C*16:02, C*16:04* |
| C | 152 | V | - | 0.0004 | 0.0000 | 0.00 | 3.03E-01 |  | *C*01:06, C*03:36* |
| C | 152 | E | - | 0.2998 | 0.3363 | 1.18 | 7.42E-04 |  | *C*01:02, C*01:03, C*02:02, C*03:02, C*03:03, C*03:04, C*03:17, C*04:01, C*04:03, C*05:01, C*06:02, C*12:02, C*12:03, C*14:02, C*14:03, C*15:02, C*15:04, C*15:05, C*15:17* |
| C | 156 | D | - | 0.0028 | 0.0050 | 1.76 | 1.22E-01 | 6.84E-08 | *C*07:04* |
| C | 156 | Q | - | 0.0009 | 0.0007 | 0.75 | 7.35E-01 |  | *C*16:02* |
| C | 156 | L | - | 0.3526 | 0.3317 | 0.91 | 5.88E-02 |  | *C*03:02, C*03:03, C*03:04, C*03:17, C*03:36, C*07:01, C*07:02, C*07:43, C*08:01, C*15:02, C*15:04, C*15:05, C*15:17* |
| C | 156 | W | - | 0.0583 | 0.0281 | 0.47 | 1.35E-09 |  | *C*02:02, C*06:02, C*12:02, C*12:03, C*16:04* |
| C | 156 | R | - | 0.2906 | 0.2979 | 1.04 | 4.92E-01 |  | *C*01:02, C*01:03, C*01:06, C*04:01, C*04:03, C*05:01, C*14:02, C*14:03* |
| C | 163 | L | - | 0.3255 | 0.3203 | 0.98 | 6.35E-01 | 3.01E-01 | *C*03:02, C*03:03, C*03:04, C*03:17, C*03:36* |
| C | 163 | E | - | 0.0021 | 0.0007 | 0.34 | 1.44E-01 |  | *C*02:02* |
| C | 163 | T | - | 0.3275 | 0.3210 | 0.97 | 5.49E-01 |  | *C*01:02, C*01:03, C*01:06, C*04:01, C*04:03, C*05:01, C*06:02, C*07:01, C*07:02, C*07:04, C*07:43, C*08:01, C*12:02, C*12:03, C*14:02, C*14:03, C*15:02, C*15:04, C*15:05, C*15:17, C*16:02, C*16:04* |
| C | 173 | K | E | 0.3255 | 0.3203 | 0.98 | 6.35E-01 |  | *C*03:02, C*03:03, C*03:04, C*03:17, C*03:36* |
| C | 177 | K | E | 0.1125 | 0.1167 | 1.04 | 5.64E-01 |  | *C*05:01, C*07:04, C*08:01* |
| C | 184 | P | H | 0.1884 | 0.2244 | 1.25 | 1.18E-04 |  | *C*07:01, C*07:02, C*07:04* |
| C | 193 | L | P | 0.0011 | 0.0007 | 0.63 | 5.67E-01 |  | *C*16:02, C*16:04* |
| C | 194 | L | V | 0.1884 | 0.2244 | 1.25 | 1.18E-04 |  | *C*07:01, C*07:02, C*07:04* |
| C | 211 | T | A | 0.0021 | 0.0007 | 0.34 | 1.44E-01 |  | *C*02:02* |
| C | 219 | R | W | 0.3841 | 0.3824 | 0.99 | 8.80E-01 |  | *C*02:02, C*05:01, C*06:02, C*07:01, C*07:02, C*07:04, C*08:01, C*12:02, C*12:03, C*15:02, C*15:04, C*15:05, C*15:17, C*16:02, C*16:04* |
| C | 248 | M | R | 0.2001 | 0.2223 | 1.14 | 1.93E-02 |  | *C*01:02, C*01:03* |
| C | 253 | Q | E | 0.1884 | 0.2244 | 1.25 | 1.18E-04 |  | *C*07:01, C*07:02, C*07:04* |
| C | 261 | M | V | 0.1884 | 0.2244 | 1.25 | 1.18E-04 |  | *C*07:01, C*07:02, C*07:04* |
| C | 267 | Q | P | 0.1884 | 0.2244 | 1.25 | 1.18E-04 |  | *C*07:01, C*07:02, C*07:04* |
| C | 273 | S | R | 0.1884 | 0.2244 | 1.25 | 1.18E-04 |  | *C*07:01, C*07:02, C*07:04* |
| C | 275 | G | - | 0.1100 | 0.1115 | 1.02 | 8.37E-01 | 4.60E-01 | *C*05:01, C*08:01* |
| C | 275 | K | - | 0.0506 | 0.0429 | 0.84 | 1.25E-01 |  | *C*04:01, C*04:03* |
| C | 275 | E | - | 0.1605 | 0.1544 | 0.95 | 4.71E-01 |  | *C*01:02, C*01:03, C*02:02, C*03:02, C*03:03, C*03:04, C*06:02, C*07:01, C*07:02, C*07:04, C*12:02, C*12:03, C*14:02, C*14:03, C*15:02, C*15:04, C*15:05, C*15:17, C*16:02, C*16:04* |
| C | 285 | M | V | 0.1884 | 0.2244 | 1.25 | 1.18E-04 |  | *C*07:01, C*07:02, C*07:04* |
| C | 295 | V | A | 0.1884 | 0.2244 | 1.25 | 1.18E-04 |  | *C*07:01, C*07:02, C*07:04* |
| C | 303 | M | V | 0.0276 | 0.0275 | 1.00 | 9.75E-01 |  | *C*04:01* |
| C | 304 | M | V | 0.1925 | 0.1565 | 0.78 | 6.15E-05 |  | *C*05:01, C*06:02, C*08:01, C*12:02, C*12:03, C*15:02, C*15:04, C*15:05, C*15:17* |
| C | 305 | T | A | 0.1884 | 0.2244 | 1.25 | 1.18E-04 |  | *C*07:01, C*07:02, C*07:04* |
| C | 306 | A | V | 0.1884 | 0.2244 | 1.25 | 1.18E-04 |  | *C*07:01, C*07:02, C*07:04* |
| C | 307 | M | V | 0.1884 | 0.2244 | 1.25 | 1.18E-04 |  | *C*07:01, C*07:02, C*07:04* |
| C | 326 | C | S | 0.1884 | 0.2244 | 1.25 | 1.18E-04 |  | *C*07:01, C*07:02, C*07:04* |
| C | 339 | T | A | 0.1884 | 0.2244 | 1.25 | 1.18E-04 |  | *C*07:01, C*07:02, C*07:04* |

*:The frequency and odds ratio are given for allele A1.For amino acid positions with more than two alleles, *P*-value for the omnibus test that tests all amino acid alleles simultaneously (with >1 degrees of freedom) for association to control.( *N* = 4,055 )
