# Supplementary material for: The Principal Genetic Determinants for Nasopharyngeal Carcinoma in China Involve the HLA Class I Antigen Recognition Groove
Source: PLoS Genet. 2012 Nov 29;8(11):e1003103. doi: 10.1371/journal.pgen.1003103 (PMC3510037; doi:10.1371/journal.pgen.1003103)
Supplement: Table S10 — The five most significant NPC associated amino acid in each HLA class I locus. (DOCX) [file pgen.1003103.s017.docx]

**Table S10. The five most significant NPC associated amino acid and the most significant NPC associated allele in each HLA class I locus (*N* = 4,055).**

| **Locus** | **Amino acid position** | **residue** | **Frequency in cases** | **Frequency in controls** | **OR** | ***P*-value** | **Represent Alleles** |
| --- | --- | --- | --- | --- | --- | --- | --- |
| HLA-A | 62 | Q | 0.2623 | 0.3753 | 0.59 | 1.17E-24 | *A*01:01, A*01:03, A*03:01, A*03:02, A*11:01, A*11:02, A*11:04, A*30:01, A*30:02, A*31:01, A*32:01, A*32:03, A*74:02* |
| HLA-A | 276 | L | 0.2150 | 0.3195 | 0.58 | 3.29E-23 | *A*01:01, A*01:03, A*03:01, A*03:02, A*11:01, A*11:04, A*30:01, A*30:02* |
| HLA-A | 114 | R | 0.2555 | 0.3608 | 0.61 | 5.74E-22 | *A*01:01, A*01:03, A*03:01, A*03:02, A*11:01, A*11:02, A*11:04, A*29:01, A*29:02, A*68:01* |
| HLA-A | 70 | Q | 0.2623 | 0.3670 | 0.61 | 1.47E-21 | *A*03:01, A*03:02, A*11:01, A*11:02, A*11:04, A*24:07, A*29:01, A*29:02, A*30:01, A*34:01, A*66:01, A*68:01, A*68:02, A*69:01* |
| HLA-A | 97 | I | 0.2591 | 0.3598 | 0.62 | 3.05E-20 | *A*01:01, A*03:01, A*03:02, A*11:01, A*11:02, A*11:04, A*30:01, A*30:02* |
| HLA-A | 11:01 |  | 0.2025 | 0.2958 | 0.59 | 1.72E-19 |  |
|  |  |  |  |  |  |  |  |
| HLA-B | -16 | L | 0.1726 | 0.2439 | 0.65 | 1.70E-13 | *B*13:01, B*13:02, B*18:01, B*18:02, B*27:03, B*27:04, B*27:05, B*27:06, B*37:01, B*40:02, B*40:03, B*40:06, B*44:02, B*44:03, B*54:01, B*55:01, B*55:02, B*56:01, B*59:01* |
| HLA-B | 116 | L | 0.1406 | 0.2068 | 0.63 | 2.37E-13 | *B*13:01, B*13:02, B*45:01, B*49:01, B*50:01, B*54:01, B*55:01, B*55:02, B*55:03, B*55:07, B*56:01, B*56:04, B*56:10, B*59:01* |
| HLA-B | 97 | R | 0.1441 | 0.2036 | 0.66 | 4.41E-11 | *B*13:01, B*15:01, B*15:02, B*15:03, B*15:08, B*15:10, B*15:11, B*15:12, B*15:13, B*15:18, B*15:21, B*15:25, B*15:27, B*15:32, B*18:01, B*35:01, B*35:02, B*35:03, B*35:08, B*37:01, B*38:01, B*38:02, B*39:01, B*39:05, B*39:09, *39:15, B*40:01, B*44:02, B*44:03, B*45:01, B*46:01, B*48:03, B*49:01, B*50:01, B*51:06, B*56:04, B*56:10, B*58:01, B*67:01* |
| HLA-B | 158 | T | 0.1338 | 0.0945 | 1.48 | 5.96E-08 | *B*38:01, B*38:02, B*39:01, B*39:05, B*39:09, B*39:15, B*67:01* |
| HLA-B | 116 | F | 0.1416 | 0.1025 | 1.45 | 1.58E-07 | *B*35:03, B*37:01, B*38:01, B*38:02, B*39:01, B*39:05, B*39:09, B*39:15, B*67:01* |
| HLA-B | 55:02 |  | 0.0099 | 0.0028 | 0.27 | 1.57E-10 |  |
|  |  |  |  |  |  |  |  |
| HLA-C | 156 | W | 0.0281 | 0.0583 | 0.47 | 1.35E-09 | *C*02:02, C*06:02, C*12:02, C*12:03, C*16:04* |
| HLA-C | 24 | S | 0.4562 | 0.4030 | 1.24 | 3.88E-06 | *C*01:02, C*01:03, C*01:06, C*06:02, C*07:01, C*07:02, C*07:04, C*07:43* |
| HLA-C | 95 | I | 0.1726 | 0.2155 | 0.76 | 4.48E-06 | *C*03:03, C*03:04, C*07:43, C*15:02, C*15:04, C*15:05, C*15:17* |
| HLA-C | 95 | L | 0.1776 | 0.2183 | 0.77 | 1.50E-05 | *C*01:02, C*01:03, C*01:06, C*02:02, C*03:02, C*03:17, C*03:36, C*04:01, C*04:03, C*05:01, C*06:02, C*07:01, C*07:02, C*08:01, C*12:02, C*12:03, C*14:02, C*14:03, C*16:02, C*16:04* |
| HLA-C | 304 | M | 0.1565 | 0.1925 | 0.78 | 6.15E-05 | *C*05:01, C*06:02, C*08:01, C*12:02, C*12:03, C*15:02, C*15:04, C*15:05, C*15:17* |
| HLA-C | 12:02 |  | 0.0093 | 0.0041 | 0.41 | 4.28E-05 |  |
