# Supplementary material for: The Principal Genetic Determinants for Nasopharyngeal Carcinoma in China Involve the HLA Class I Antigen Recognition Groove
Source: PLoS Genet. 2012 Nov 29;8(11):e1003103. doi: 10.1371/journal.pgen.1003103 (PMC3510037; doi:10.1371/journal.pgen.1003103)
Supplement: Table S11 — Multivariate logistic regression analysis for significant variant and HLA class I alleles. (DOCX) [file pgen.1003103.s018.docx]

**Table S11. Multivariate logistic regression analysis for significant variant and HLA class I alleles (*N*=2,028; row III in eTable 1)**

*: We used PLINK to examine the residual effect of index variant 1 while using variant 2 as a covariate, and we adjusted the results for age and gender. The color code from red to green is represented significant power from strong to weak.
