# Supplementary material for: The Principal Genetic Determinants for Nasopharyngeal Carcinoma in China Involve the HLA Class I Antigen Recognition Groove
Source: PLoS Genet. 2012 Nov 29;8(11):e1003103. doi: 10.1371/journal.pgen.1003103 (PMC3510037; doi:10.1371/journal.pgen.1003103)
Supplement: Table S12 — HLA-A, -B and -C allele association analysis separated into in phase I and II analyses. (DOCX) [file pgen.1003103.s019.docx]

**Table S12. HLA-A, -B and -C allele association analysis separated into in phase I and II analyses.**

|  | Allele frequencies | | | NPC vs. Controls | | NPC VS. E.P. Controls | | NPC VS. E.N. Controls | | E.P. Controls vs. E.N. Controls | |
| --- | --- | --- | --- | --- | --- | --- | --- | --- | --- | --- | --- |
| Allele name | NPC cases | E.P. Controls* | E.N. Controls^†^ | OR (95% CI) | *P*-value | OR (95% CI) | *P*-value | OR (95% CI) | *P*-value | OR (95% CI) | *P*-value |
| Phase I^§^ | N = 356 | N = 287 | N = 342 |  |  |  |  |  |  |  |  |
| *HLA-A* |  |  |  |  |  |  |  |  |  |  |  |
| *02:03* | 15.45(110) | 13.76(79) | 16.52(113) |  | Ns |  | Ns |  | Ns |  | Ns |
| *02:06* | 5.20(37) | 2.09(12) | 2.49(17) |  | Ns |  | Ns |  | Ns |  | Ns |
| *02:07* | 14.89(106) | 11.15(64) | 11.40(78) |  | Ns |  | Ns |  | Ns |  | Ns |
| *11:01* | 19.52(139) | 28.57(164) | 29.53(202) | 0.58(0.45-0.73) | 5.99E-06 | 0.59(0.45-0.79) | 3.08E-04 | 0.57(0.43-0.74) | 3.67E-05 |  | Ns |
| *33:03* | 20.51(146) | 14.63(84) | 14.04(96) | 1.64(1.26-2.12) | 1.92E-04 |  | Ns |  | Ns |  | Ns |
| *HLA-B* |  |  |  |  |  |  |  |  |  |  |  |
| *13:01* | 7.72(55) | 12.89(74) | 11.40(78) |  | Ns |  | Ns |  | Ns |  | Ns |
| *27:04* | 0(0) | 1.39(8) | 1.17(8) |  | Ns |  | Ns |  | Ns |  | Ns |
| *38:02* | 12.94(90) | 8.19(47) | 10.23(70) |  | Ns |  | Ns |  | Ns |  | Ns |
| *46:01* | 17.13(122) | 17.25(99) | 14.04(96) |  | Ns |  | Ns |  | Ns |  | Ns |
| *55:02* | 0.56(4) | 3.83(22) | 3.51(24) | 0.15(0.05-0.42) | 3.56E-04 | 0.13(0.04-0.40) | 3.48E-04 | 0.16(0.06-0.39) | 9.50E-03 |  | Ns |
| *58:01* | 19.10(136) | 13.07(75) | 14.91(102) |  | Ns |  | Ns |  | Ns |  | Ns |
| *HLA-C* |  |  |  |  |  |  |  |  |  |  |  |
| *01:02* | 18.96(135) | 21.95(126) | 17.54(120) |  | Ns |  | Ns |  | Ns |  | Ns |
| *03:02* | 18.12(129) | 12.20(70) | 14.47(99) |  | Ns |  | Ns |  | Ns |  | Ns |
| *07:02* | 21.49(153) | 17.25(99) | 19.15(131) |  | ns |  | Ns |  | Ns |  | Ns |
| *12:02* | 0.56(4) | 2.96(17) | 1.61(11) |  | Ns |  | Ns |  | Ns |  | Ns |
| *12:03* | 0.56(4) | 2.44(14) | 1.75(12) |  | Ns |  | Ns |  | Ns |  | Ns |
| Phase II | N = 1,049 | N = 1,001 | N = 1,020 |  |  |  |  |  |  |  |  |
| *HLA-A* |  |  |  |  |  |  |  |  |  |  |  |
| *02:03* | 15.87(333) | 10.04(201) | 14.51(296) | 1.33(1.15-1.55) | 1.85E-04 | 1.67(1.38-2.02) | 1.31E-07 |  | Ns | 0.66(0.54-0.80) | 1.73E-05 |
| *02:06* | 3.86(81) | 2.25(45) | 3.19(65) |  | Ns |  | Ns |  | Ns |  | Ns |
| *02:07* | 17.02(357) | 13.94(279) | 11.62(237) | 1.40(1.21-1.63) | 6.51E-06 |  | Ns | 1.56(1.31-1.86) | 9.02E-07 |  | Ns |
| *11:01* | 20.50(430) | 30.52(611) | 28.97(591) | 0.60(0.52-0.68) | 6.90E-15 | 0.56(0.48-0.66) | 1.06E-13 | 0.61(0.53-0.71) | 1.37E-10 |  | Ns |
| *33:03* | 16.73(351) | 16.08(322) | 11.81(241) |  | Ns |  | Ns | 1.54(1.28-1.85) | 3.58E-06 | 1.43(1.19-1.71) | 1.44E-04 |
| *HLA-B* |  |  |  |  |  |  |  |  |  |  |  |
| *13:01* | 8.15(171) | 12.09(242) | 10.69(218) | 0.69(0.57-0.83) | 7.18E-05 | 0.64(0.52-0.79) | 3.67E-05 |  | Ns |  | Ns |
| *27:04* | 0.71(15) | 0.85(17) | 1.86(38) |  | Ns |  | Ns |  | Ns |  | Ns |
| *38:02* | 12.06(253) | 6.44(129) | 7.89(161) | 1.75(1.47-2.10) | 7.59E-10 | 1.97(1.57-2.47) | 4.33E-09 | 1.59(1.29-1.95) | 1.42E-05 |  | Ns |
| *46:01* | 19.88(417) | 18.18(364) | 14.61(298) |  | Ns |  | Ns | 1.43(1.22-1.68) | 1.05E-05 |  | Ns |
| *55:02* | 1.14(24) | 3.25(65) | 3.73(76) | 0.31(0.20-0.48) | 1.70E-07 | 0.32(0.20-0.52) | 3.74E-06 | 0.29(0.18-0.47) | 2.69E-07 |  | Ns |
| *58:01* | 15.82(332) | 16.18(324) | 11.32(231) |  | Ns |  | Ns | 1.49(1.24-1.79) | 2.17E-05 | 1.49(1.24-1.79) | 2.14E-05 |
| *HLA-C* |  |  |  |  |  |  |  |  |  |  |  |
| *01:02* | 23.07(484) | 21.18(424) | 18.14(370) |  | Ns |  | Ns | 1.34(1.16-1.57) | 1.09E-04 |  | Ns |
| *03:02* | 15.78(331) | 16.08(322) | 11.13(227) |  | Ns |  | Ns | 1.52(1.26-1.83) | 1.01E-05 | 1.51(1.26-1.82) | 1.23E-05 |
| *07:02* | 21.69(455) | 16.78(336) | 19.46(397) |  | Ns | 1.37(1.16-1.61) | 1.67E-04 |  | Ns |  | Ns |
| *12:02* | 1.05(22) | 1.20(24) | 3.24(66) |  | Ns |  | Ns | 0.32(0.20-0.52) | 4.37E-06 | 0.39(0.25-0.63) | 9.01E-05 |
| *12:03* | 0.86(18) | 2.05(41) | 1.76(36) |  | Ns |  | Ns |  | Ns |  | Ns |

*: E. P. controls: EBV IgA/VCA positive controls

†: E. N. controls: EBV IgA/VCA negative controls

§: A preliminary analysis of HLA association in phase I was previously reported[1].

1. Tang M, Zeng Y, Poisson A, Marti D, Guan L, et al. (2010) Haplotype-dependent HLA susceptibility to nasopharyngeal carcinoma in a Southern Chinese population. Genes Immun 11: 334-342.
